# From Nanoparticles to Single Crystals of Al‐MOFs: Synergistic Coordination and pH Modulation, and Rapid Sorption Kinetics Assessment by Optical Calorimetry

**DOI:** 10.1002/chem.202501110

**Published:** 2025-06-10

**Authors:** Bastian Achenbach, Christoph Meier, Norbert Stock

**Affiliations:** ^1^ Institute of Inorganic Chemistry Kiel University Max‐Eyth‐Str. 2 24118 Kiel Germany; ^2^ Kiel Nano Surface and Interface Science KiNSIS Kiel University Christian‐Albrechts‐Platz 4 24118 Kiel Germany

**Keywords:** adsorption, aluminium carboxylates, crystal growth, metal‐organic frameworks, nanoparticles

## Abstract

The properties of metal‐organic frameworks (MOFs), such as sorption kinetics or mechanical and chemical stability, not only depend on their composition and chemical structure, but also on their crystal size and morphology. However, the tunability of the crystallite size of aluminum‐based MOFs (Al‐MOFs) is still a long‐standing challenge. In this study, we present systematic high‐throughput investigations elucidating the synergistic effects of different mono‐ and dicarboxylic acids (acetic acid, malonic acid, and oxalic acid) as coordination modulators and NaOH as a pH modulator on the crystal size of various Al‐MOFs, with a focus on Al‐MIL‐53‐NO_2_. By varying the type and amount of coordination modulators, we successfully extended the range of achievable particle sizes to nanoparticles as well as large crystals (*d*_max_ ≈ 100 nm – 800 µm) compared to traditional synthesis methods using only coordination modulators. Thus, large crystals as well as nanoparticles of different Al‐MOFs could be obtained by simply varying the molar ratio of the different modulators. Additionally, we explored the influence of particle size on CO_2_ sorption properties using InfraSORP technology (optical calorimetry), revealing the increase in adsorption rates with decreasing particle size.

## Graphical abstract

High‐throughput studies were carried out to elucidate the synergistic effects of coordination modulation and pH modulation on the crystal size of different Al‐MOFs, resulting in nanoparticles as well as large crystals (*d*_max_ ≈ 0.1 µm – 800 µm). In addition, the influence of particle size on CO_2_ sorption properties was investigated, showing increasing adsorption and desorption rates with decreasing particle size.

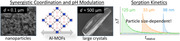

## 1 Introduction

Metal‐organic frameworks (MOFs) are a class of potentially porous materials composed of inorganic building units (IBUs) and organic ligands, representing a subclass of coordination polymers.^[^ ^1^, ^2^ ^]^ The modular structure of MOFs, resulting in high structural diversity and tunability, coupled with their host‐guest chemistry, make them promising candidates for applications such as gas separation,^[^ ^3^, ^4^ ^]^ catalysis,^[^ ^5^ ^]^ sensing,^[^ ^6^, ^7^ ^]^ and drug delivery.^[^ ^8^ ^]^ Among the MOFs based on trivalent metal ions, aluminum‐based MOFs (Al‐MOFs) have attracted particular interest in recent years due to their high chemical, thermal and hydrolytic stability,^[^ ^9^, ^10^, ^11^, ^12^, ^13^ ^]^ the high availability and low cost of starting materials,^[^ ^14^, ^15^ ^]^ and the light weight of Al‐MOFs combined with permanent porosity, enabling potential industrial applications in areas such as gas storage and separation,^[^ ^4^, ^16^, ^17^ ^]^ and heat exchange processes.^[^ ^14^, ^18^, ^19^, ^20^ ^]^

The properties of MOFs not only depend on their composition and chemical structure, but also on their crystal size and morphology.^[^ ^21^, ^22^ ^]^ Decreasing the particle size leads to shorter diffusion paths, which are important for applications in catalysis or gas storage and separation. On the other hand, the synthesis of MOF single crystals enables the detailed description of their structural properties by single‐crystal X‐ray diffraction (SCXRD) as well as the investigation of functional properties, such as proton or electronic conductivity^[^ ^23^, ^24^, ^25^, ^26^, ^27^ ^]^ along different crystal axis and mechanical response.^[^ ^28^, ^29^, ^30^ ^]^ Single crystals are therefore highly valuable for establishing structure‐property relationships. In addition, the decisive role of the crystal size affecting the breathing kinetics of structurally flexible MOFs, such as Al‐MIL‐53 type materials, with respect to different crystallite size regimes has been demonstrated by Bon et al.^[^ ^31^ ^]^ Therefore, a number of different approaches have been taken to control the size and morphology of MOF crystals. However, especially the synthesis of large single crystals of Al‐MOFs is still a long‐standing challenge, as most of these can only be obtained as nano‐ or microcrystalline powders.^[^ ^22^, ^32^, ^33^ ^]^

An approach that has been considered to increase the crystal‐size of Al‐MOFs is the use of hydrofluoric acid (HF) as a mineralizer/modulator.^[^ ^22^, ^34^, ^35^ ^]^ However, the use of HF as a modulator raises concerns on safety due to its toxicity^[^ ^36^ ^]^ and low synthesis yields.^[^ ^37^, ^38^ ^]^ Furthermore, fluoride ions were often incorporated into the network or secondary phases were formed during the preparation of Al‐MOFs using HF as a synthesis modulator.^[^ ^39^ ^]^ As an alternative to HF for the synthesis of highly crystalline Al‐MOFs, the use of so‐called coordination modulators,^[^ ^40^, ^41^, ^42^ ^]^ such as formic acid and acetic acid, competing with the available linker molecules for coordination to the metal center, were investigated. The coordination of the modulator to the metal center can influence crystal growth,^[^ ^43^, ^44^ ^]^ defect formation,^[^ ^45^ ^]^ surface properties,^[^ ^46^ ^]^ or the preferred formation of a particular phase.^[^ ^47^ ^]^ Canossa et al. presented a method based on the use of oxalic acid (ethanedicarboxylic acid; H_2_Ox; H_2_C_2_O_4_)^[^ ^39^ ^]^ as a coordination modulator to increase the crystal size of Al‐MOFs up to 200 µm without affecting the MOFs composition.^[^ ^39^, ^48^ ^]^ Oxalic acid is a naturally occurring, inexpensive, and readily available dicarboxylic acid and Canossa et al. were able to show that the addition of oxalic acid to the aqueous reaction mixture can strongly influence the crystallinity, crystal size, and morphology of Al‐MOFs.

Here we present the synergistic effect on the influence of different coordination modulators in combination with NaOH as a pH modulator. High‐throughput (HT) methods were used to screen the influence of modulator concentrations and synthesis parameters, such as reaction temperature and overall concentrations on the crystal size and morphology of eight Al‐MOFs, mainly with MIL‐53 type structure. Large crystals (up to *d*_max_ ≈ 800 µm) of various Al‐MOFs as well as nanoparticles (≤100 nm) of MIL‐53‐NO_2_ could be obtained. Additionally, the particle size‐dependent CO_2_ sorption properties (adsorption capacity and sorption kinetics) of Al‐MIL‐53‐NO_2_ are reported.

## 2 Results and Discussion

Systematic HT studies were used to demonstrate synergistic effects of different coordination modulators and NaOH as the pH modulator on the crystal size and morphology of different Al‐MOFs. Al‐MIL‐53‐NO₂ with nitroterepthalic acid (H_2_BDC‐NO_2_) as the linker was selected for a detailed study to investigate the effects of varying reaction temperatures and molar ratios of different modulators, since initial results indicated significant variation in accessible crystal sizes. Additionally, the residual linker can be easily removed for Al‐MIL‐53‐NO_2_ while maintaining the size and stability of the crystals. The results of the initial HT optimization for Al‐MIL‐53‐NO_2_ were subsequently used to synthesize various Al‐MOFs including Al‐MIL‐53‐X (with X = ‐H,^[^ ^49^ ^]^ ‐OH, ‐Br,^[^ ^11^ ^]^ ‐COOH, ‐(COOH)_2_ (MIL‐121)^[^ ^50^ ^]^), Al‐MIL‐96,^[^ ^51^ ^]^ Al‐MIL‐118A,^[^ ^50^ ^]^ and Al(OH) (1,4‐NDC)^[^ ^52^ ^]^ as large crystals. The Al‐MOFs obtained under the optimized synthesis conditions were characterized by powder X‐ray diffraction (PXRD) and infrared (IR) spectroscopy, ^1^H and ^13^C‐NMR spectroscopy, and thermogravimetric measurements. Phase purity was confirmed by Le Bail fits against the PXRD data and the crystal structure of Al‐MIL‐53‐COOH containing water molecules was exemplarily elucidated by single crystal X‐ray diffraction (SCXRD). Given the potential for producing Al‐MIL‐53‐NO₂ in a large range of particle sizes, and the relatively high affinity of this Al‐MOF toward CO₂,^[^ ^11^ ^]^ we conducted preliminary tests on the particle size–dependent sorption properties of using InfraSORP technology.

### 2.1 Synthesis

#### 2.1.1 Screening of Different Coordination Modulators

**Figure 1:**
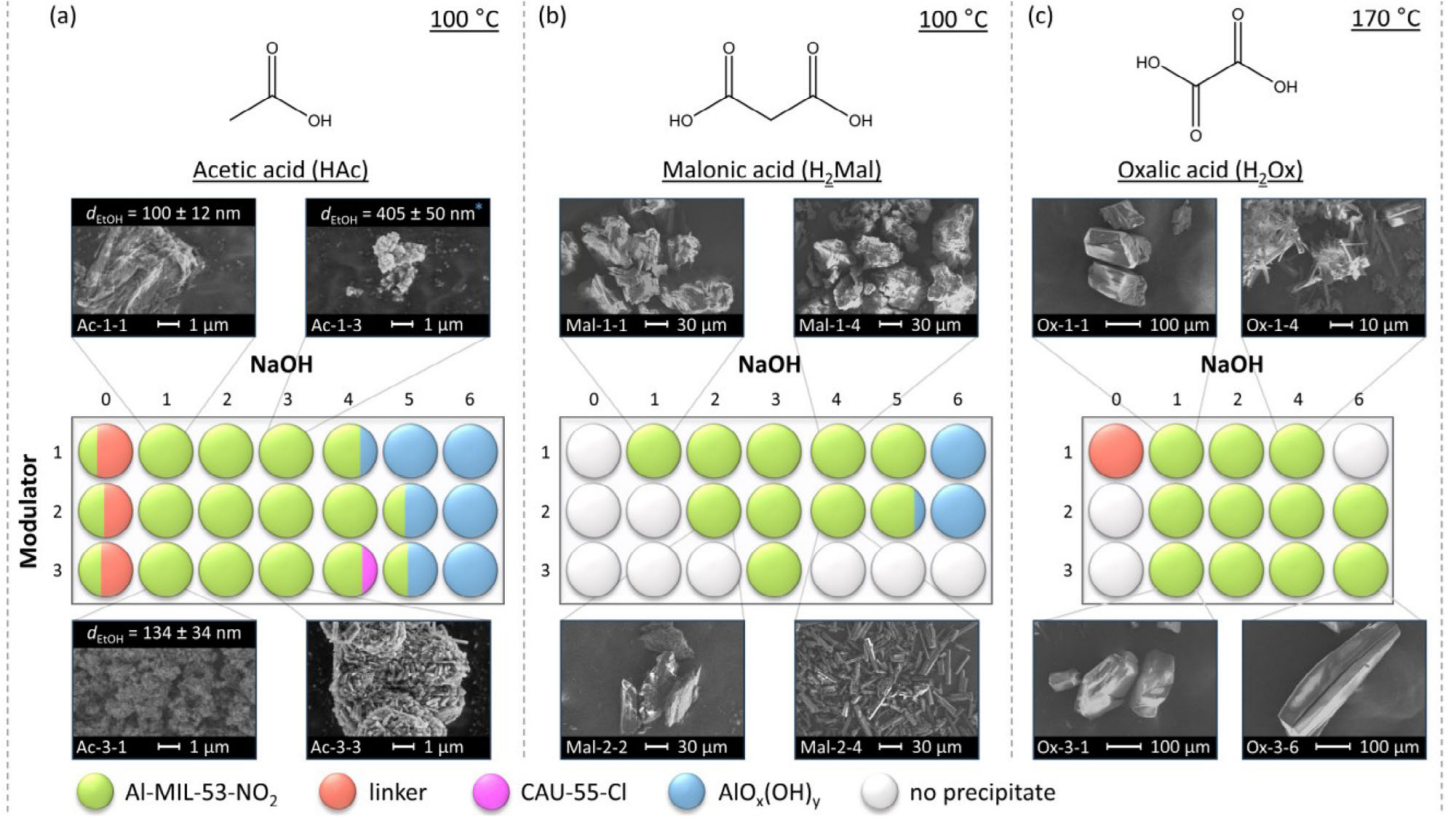
(**a–c**) Crystallization diagrams of crystalline phases observed in the chemical system AlCl_3_ / H_2_BDC‐NO_2_ / coordination modulator / NaOH in water for reactions carried out employing acetic acid (**a**), malonic acid (**b**) and oxalic acid (**c**) as the coordination modulator. The molar ratios of coordination modulator and pH modulator were varied between 1 and 3 and 0 and 6 equivalents, while keeping the AlCl_3_ and H_2_BDC‐NO_2_ concentration constant with a molar ratio of 1 : 1. The crystalline phases obtained are color‐coded and grey circles represent parameter spaces where clear solutions were obtained. For some reaction, different polymorphs of AlO_x_(OH)_y_ (blue circles) or crystalline linker (red circles) were obtained as a crystalline side‐product and in the reaction Ac‐3–4, CAU‐55‐Cl, a porous salt of composition [Al_24_(OH)_56_(O_2_CCH_3_)_12_]Cl_4_,^[^ ^59^ ^]^ was found. SEM micrographs (scale bar = 1, 10, 30, 100 µm) of Al‐MIL‐53‐NO_2_ crystals obtained with the different mono‐ and ditopic synthesis modulators are shown for some reaction products. For the samples with particle sizes < 1 µm suitable for dynamic light scattering (DLS), the corresponding particle sizes (based on the number distribution) are given. *For the sample Ac‐1–3, an additional peak in the particle size distribution was obtained in the DLS measurements at > 3000 nm. The samples are labelled according to the molar ratios of the coordination modulator and pH modulator used in the synthesis. For example, sample Ac‐1–3 describes a sample that was obtained with one equivalent of acetic acid as the coordination modulator and three equivalents of NaOH as the pH modulator.

Systematic high‐throughput (HT) studies of the chemical system AlCl_3_/nitroterephthalic acid (H_2_BDC‐NO_2_)/coordination modulator/NaOH in water were used to investigate the effects of three different coordination modulators on the crystal size and morphology of Al‐MIL‐53‐NO_2_. Acetic acid (HAc) was used as a monotopic and malonic and oxalic acid (H_2_Mal and H_2_Ox, respectively) as ditopic coordination modulators, and the molar ratios of coordination modulator and pH modulator were varied between 1 and 3 and 0 and 6 equivalents, while keeping the AlCl_3_ and H_2_BDC‐NO_2_ concentration constant with a molar ratio of 1:1 (Figure 1). The samples are labelled according to the molar ratios of the coordination modulator and pH modulator used in the synthesis (type of coordination modulator, amount of coordination modulator, and amount of pH modulator). For example, sample Ac‐1–3 describes a sample that was obtained using the molar ratios Al^3+^ : H_2_BDC‐NO_2_ : HAc : NaOH = 1:1:1:3, that is, with one equivalent of acetic acid as the coordination modulator and three equivalents of NaOH as the pH modulator. The reactions were carried out at a reaction temperature of 100 °C for HAc and H_2_Mal modulated syntheses, whereas higher reaction temperatures (*T* = 170 °C) were required to obtain crystalline products when H_2_Ox was used as the modulator. Section S2 of the Supporting Information provides further details regarding the synthesis conditions and PXRD data obtained for all samples used to visualize the results of the HT investigations (Figure 1). The following trends can be extracted from the data. At high NaOH and low modulator concentrations, different polymorphs of AlO_x_(OH)_y_^[^ ^53^ ^]^ (blue circles) or clear solutions with no precipitate (gray circles) were obtained, for example, Ac‐1–6 and Ox‐1–6.

- The largest crystals of Al‐MIL‐53‐NO_2_ were observed when high amounts of coordination modulator and pH modulator are used, for example, Ac‐3–3, Mal‐2–4, and Ox‐3–6. This corresponds to low pH values of the reaction mixture (pH ≈ 0 – 1).
- Nano‐ or microcrystalline powders are found at lower modulator concentration and an excess of NaOH (pH ≈ 4), for example, Ac‐1–3, Mal‐1–4 , and Ox‐1–4.
- In general, crystal sizes depend on the type of modulator and increases in the order HAc < H_2_Mal < H_2_Ox, for example, Ac‐1–1 < Mal‐1–1 < Ox‐1–1.

Thus, the smallest particles of Al‐MIL‐53‐NO_2_ were obtained when a low concentration of acetic acid (HAc) and a high concentration of NaOH were used (Ac‐1–3), while the use of malonic acid (H_2_Mal) as the modulator led to the formation of microcrystalline powders or larger crystals of up to 100 µm in length (Mal‐2–4). With oxalic acid (H_2_Ox), Al‐MIL‐53‐NO_2_ was obtained in high crystallinity with crystal sizes between 10 and 500 µm depending on the molar ratio of oxalic acid: NaOH (Ox‐1–4 and Ox‐3–6). This variation in the influence of the different coordination modulators on the crystal size of the Al‐MOFs can be explained by the formation of stable chelate complexes of oxalate and malonate ions with the Al^3+^ ions in contrast to the monotopic coordination modulator acetic acid.^[^ ^54^, ^55^ ^]^ For instance, the formation of Al‐oxalate and ‐malonate complexes with oxalate and malonate ions acting as a chelate ligand and the high stability of the resulting five‐membered ring is well‐known in the literature.^[^ ^39^, ^54^, ^55^, ^56^ ^]^ Thus, high temperatures were necessary for reactions using oxalic acid as the coordination modulator and correlates with the different decomposition temperatures of oxalic acid species (*T*_decomposition_ ≥ 160 °C)^[^ ^57^ ^]^ compared to malonic acid (*T*_decomposition_ ≥ 120 °C).^[^ ^58^ ^]^ In addition to the formation of stable Al‐carboxylate complexes of Al^3+^ ions and the coordination modulator, the pH decrease due to the presence of the further disfavors the deprotonation of the linker and thus limiting the formation of the MOF.^[^ ^39^, ^40^ ^]^ In experiments conducted by Canossa et al. using oxalic acid as a coordination modulator, a decrease in crystallinity or an absence of crystalline product formation was often observed at high modulator concentrations. This can presumably be attributed to the low pH value at these modulator concentrations, preventing the deprotonation of the linker molecules. However, the use of a pH modulator (NaOH) in our study led to crystalline products and an increase in accessible particle sizes, particularly at high modulator concentrations.

#### 2.1.2 Optimization of the Synthesis of Large Crystals

**Figure 2:**
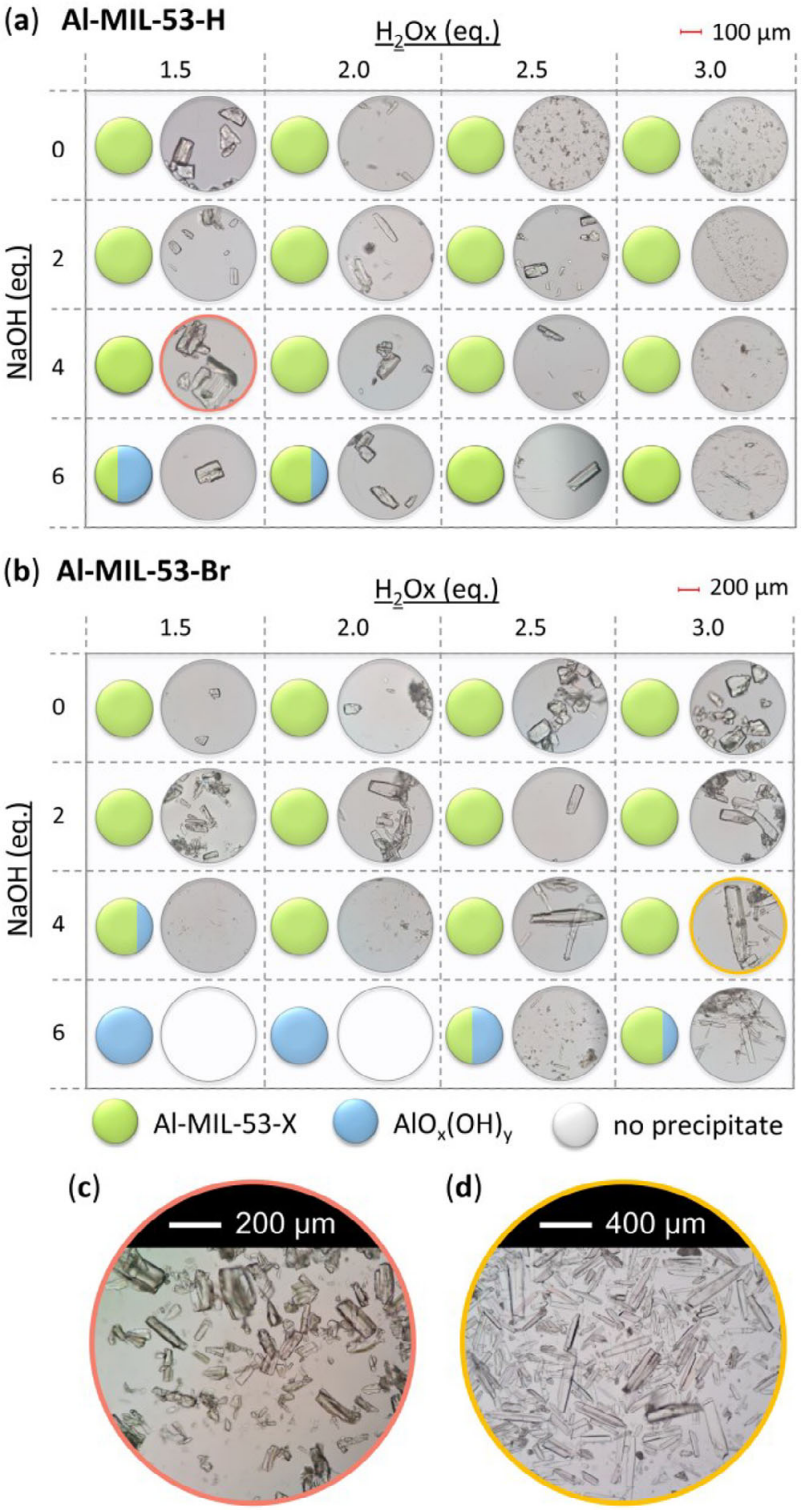
Crystallization diagram of the crystalline phases observed in the chemical systems AlCl_3_ / Linker / oxalic acid / NaOH, with Linker = H_2_BDC (**a**) and H_2_BDC‐Br (**b**) in H_2_O for reactions carried out at 200 °C (**a**) and 210 °C (**b**), keeping the concentration of AlCl_3_ and H_2_BDC constant (molar ratio Al^3+^: Linker: H_2_Ox: NaOH = 1: 1: 1.5 – 3.0: 0 – 6). The crystalline phases observed are color‐coded and for each reaction that led to Al‐MIL‐53‐H (**a**) or Al‐MIL‐53‐Br (**b**), an optical micrograph of the crystals is shown. In addition, for the optimized reaction conditions (red and yellow circle), an optical micrograph with a higher resolution and a larger number of crystals is shown. The PXRD data used to prepare the crystallization diagrams are shown in the supporting information (Figure S2.5–S2.9).

The results of the HT study with nitroterephthalic acid (H_2_BDC‐NO_2_) as a linker were used to synthesize large crystals of Al‐MOFs with different topologies, that is, Al‐MIL‐53‐X (with X = ‐H,^[^ ^49^ ^]^ ‐OH, ‐Br,^[^ ^11^ ^]^ ‐COOH,^[^ ^10^ ^]^ ‐(COOH)_2_ (MIL‐121)^[^ ^50^ ^]^), Al‐MIL‐96,^[^ ^51^ ^]^ Al‐MIL‐118A,^[^ ^50^ ^]^ and [Al(OH) (1,4‐NDC)].^[^ ^52^ ^]^ Synergistic effects of coordination and pH modulator were observed and crystals with a maximum size of *d*_max_ ≈ 50 to 800 µm could be obtained employing oxalic acid as the coordination modulator and NaOH as the pH modulator. The synthesis procedures reported for the given MOFs^[^ ^10^, ^11^, ^49^, ^50^, ^51^, ^52^ ^]^ were taken into account, that is, overall concentration, molar ratio of Al^3+^ to linker and reaction temperature were used as reported and the amounts of the modulators (H_2_Ox to NaOH) were varied. The results of this study are presented in Figure 2 for Al‐MIL‐53‐H and Al‐MIL‐53‐Br and Figures S2.6–S2.18 for the other Al‐MOFs. Very similar trends as found for Al‐MIL‐53‐NO_2_ are observed for Al‐MIL‐53‐X, with X = ‐OH, ‐Br, ‐COOH and [Al(OH) (1,4‐NDC)]. In contrast, the amount of NaOH plays a decisive role on product formation where linker deprotonation is important, that is, when terephthalic acid (Figure 2a), trimesic acid (Figure S2.15–S2.16), or 1, 2, 4, 5‐benzenetetracarboxylic acid (Figure S2.13–S2.14) are used. High amounts of NaOH were crucial for the synthesis of highly crystalline MIL‐53‐H, presumably due to the low solubility of terephthalic acid in water, while a low concentration of NaOH resulted in the largest crystals of MIL‐121 and MIL‐96. Furthermore, the addition of NaOH played a crucial role in the synthesis of the three Al‐MOFs that can be obtained with 1,2,4,5‐benzenetetracarboxylic acid (H_4_BTEC) as the linker molecule: MIL‐121, with H_2_BTEC^2−^ ions as the linker, was obtained only in the absence of NaOH, while and MIL‐118A and MIL‐120 containing BTEC^4−^ ions and were observed preferentially with increasing NaOH concentration (Figure S2.14). The optimized synthesis conditions (reaction temperature, molar ratios of AlCl_3_: Linker: Oxalic acid: NaOH) for the different Al‐MOFs leading to the largest crystals using oxalic acid modulated synthesis are summarized in Table 1.

**Table 1:** Optimized synthesis conditions (reaction temperature, molar ratios of Al^3+^: Linker: H_2_Ox: NaOH, overall concentration^[^ ^[Link]^, ^[Link]^ ^]^) leading to the largest crystals using oxalic acid as the coordination modulator and NaOH as a pH modulator under hydrothermal reaction conditions for the synthesis of various Al‐MOFs. For each reaction product, optical micrographs of the crystals obtained are shown. A larger‐scale optical micrograph of each sample is shown in the Supporting Information. The average crystal size and the corresponding standard deviation was determined by measuring the diameter of ten crystals per sample (excluding crystal fragments) using optical microscopy.

MOF + Crystal Size	Linker	*T* / °C	Al^3+^: Linker: H_2_Ox: NaOH	Optical Microscopy
Al‐MIL‐53‐H (*d* = 145 ± 30 nm)	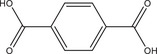	200^[a]^	1.0: 1.0: 1.5: 4.0^[b^ ^]^	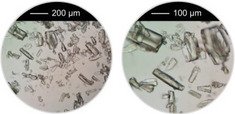
Al‐MIL‐53‐OH (*d* = 95 ± 14 nm)	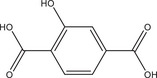	170^[^ ^a]^	1.0: 1.0: 3.0: 2.0^[b]^	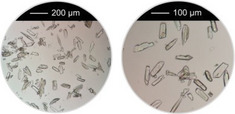
Al‐MIL‐53‐NO_2_ (*d* = 430 ± 95 nm)	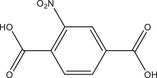	170^[a]^	1.0: 1.0: 3.0: 6.0^[c]^	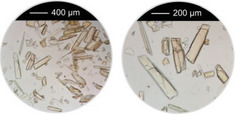
Al‐MIL‐53‐Br (*d* = 290 ± 105 nm)	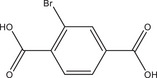	210^[a]^	1.0: 1.0: 3.0: 4.0^[b]^	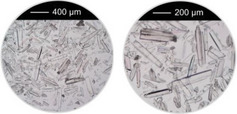
Al‐MIL‐53‐COOH (*d* = 420 ± 85 nm)	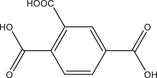	170^[a^ ^]^	1.0: 1.0: 2.5: 2.0^[b]^	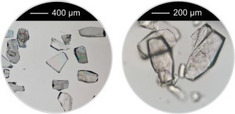
Al‐MIL‐121 (*d* = 125 ± 30 nm)	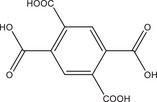	200^[a]^	1.0: 0.5: 3.0: 0.0^[d]^	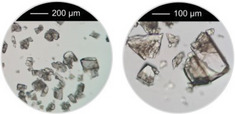
Al‐MIL‐96 (*d* = 50 ± 9 nm)	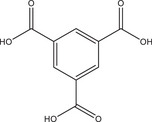	200^[a]^	1.0: 0.5: 1.5: 0.0^[b]^	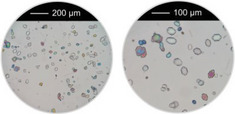
[Al(OH) (1,4‐NDC)] (*d* = 110 ± 45 nm)	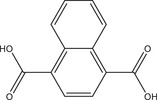	170^[a]^	1.0: 1.0: 3.0: 2.0^[c]^	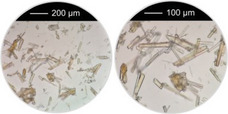

^[a]^ *t*_1_ – *t*_2_ – *t*_3_ = 6 hours – 30 hours – 6 hours. [b]–[d] *c*(1 eq) =

^[b]^ 0.18 mol/L

^[c]^ 0.09 mol/L

^[d]^ 0.64 mol/L

It is noteworthy that, in contrast to other modulated MOF syntheses,^[^ ^46^, ^60^ ^]^ only minor variations in the crystal morphology of the Al‐MOFs were observed as a function of the molar ratios of the starting materials. For example, only for Al‐MIL‐53‐Br, needle‐shaped crystals were preferentially obtained at higher NaOH concentrations, whereas block‐shaped crystals were formed in the absence of NaOH (Figure 2b).

#### 2.1.3 Synthesis of Nanoparticles in Glass Vials

**Figure 3:**
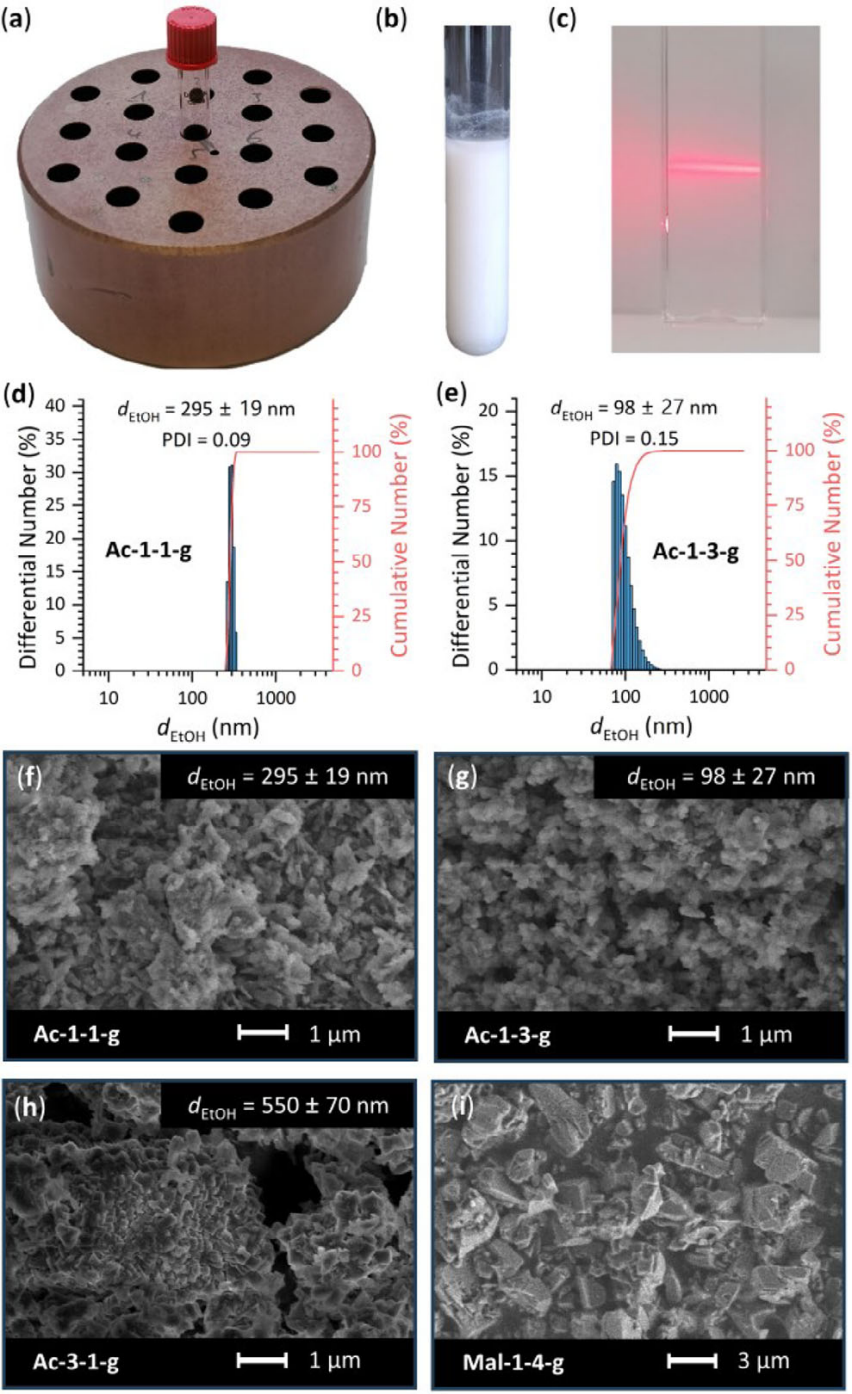
(**a**) Glass reaction vials (*V*_max_ *= *14 mL), placed in a preheated (100 °C) aluminum block. (**b**) Dispersion of nanoparticles (*d*_EtOH_ = 98 ± 27 nm) of Al‐MIL‐53‐NO_2_ (Ac‐1–3‐*g*) in a glass reactor after heating the reaction solution for 20 hours at 100 °C under stirring. (**c**) Diluted dispersion of Al‐MIL‐53‐NO_2_ nanoparticles (Ac‐1–3‐*g*) in water showing the Tyndall effect. (**d**) , (**e**) Particle size distribution (number distribution), obtained using dynamic light scattering (DLS) of Al‐MIL‐53‐NO_2_ (Ac‐1–1‐*g* (**d**), Ac‐1–3‐*g* (**e**)). No particle size distribution is shown for Mal‐1–4‐G as the particles are > 1 µm in size and therefore not suitable for DLS analysis. (**f**) – (**i**) SEM micrographs of Al‐MIL‐53‐NO_2_ (Ac‐1–1‐*g* (**d**), Ac‐1–3‐*g* (**e**), Ac‐3–1‐*g* (**f**), Mal‐1–4‐*g* (**g**)) obtained with the different mono‐ and ditopic coordination modulators (HAc, H_2_Mal) in glass reactors. The names of the products obtained describe the molar ratios of the coordination modulator and pH modulator used in the synthesis (type of coordination modulator – amount of coordination modulator – amount of pH modulator – type of reactor (“*g*” for glass reactors)).

Further investigations were carried out in 10 ml glass vials to determine whether the results and trends obtained in previous HT studies using steel autoclaves could also be applied to stirred reactions in glass reactors with larger reaction volumes. The optimized reaction conditions that led to the formation of both nanoparticles and microcrystalline powders were employed, using acetic acid and malonic acid as the coordination modulators (Ac‐1–1‐*g*, Ac‐1–3‐*g*, Ac‐3–1‐*g*, Mal‐1–4‐*g*, where “*g*” denotes a glass reactor). To ensure high heating rates and continuous stirring, the reaction vials were placed in a preheated aluminum block (100 °C (Figure 3a). Stable dispersions of Al‐MIL‐53‐NO₂ nanoparticles (*d*_EtOH_ ≤ 100 nm) with narrow particle size distributions were obtained using high NaOH:modulator molar ratios, as well as high heating rates, low reaction temperatures (100 °C) and short reaction times (≤20 C). Additionally, larger overall sample quantities were accessible using glass vials, and a decrease in the polydispersity indices obtained by dynamic light scattering (DLS) measurements (Figure S5.1) was observed compared to samples obtained in autoclaves. Furthermore, additional particle size distributions with particle sizes > 1 µm were found in the DLS data for Ac‐1–3 (see Figure S5.1e), which further indicates the presence of larger particles or agglomerates that were not observed in the corresponding reaction performed in a glass vial under stirring.

### 2.2 Characterization

**Figure 4:**
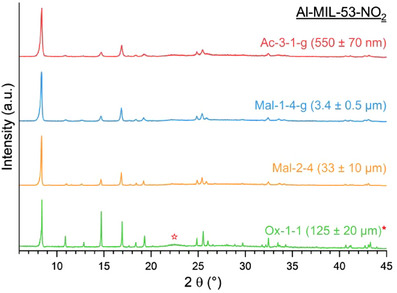
Measured PXRD patterns of four different Al‐MIL‐53‐NO_2_ samples with different particle sizes (*d* ≈ 550 nm – 125 µm) obtained using mono‐ and ditopic synthesis modulators (acetic acid (HAc), malonic acid (H_2_Mal), oxalic acid (H_2_Ox)), and NaOH as the pH modulator. To increase the reproducibility, all samples were soaked with water prior to measurement to obtain the water‐rich *large‐pore* form of Al‐MIL‐53‐NO_2__*lp*_H_2_O. *The sample was ground prior to measurement. ^☆^The broad band at 21 – 24 °2θ is related to the presence of filtration paper used to prepare the samples.

The Al‐MOFs obtained under the optimized synthesis conditions using oxalic acid as the modulator (Table 1, Section S2) were characterized by powder X‐ray diffraction (PXRD, Section S3), infrared (IR) spectroscopy (Section S4.1), NMR spectroscopy (Section S4.2), and thermogravimetric methods (Section S4.3) to confirm phase purity, the integrity of the linker molecules and the absence of modulators in the MOFs. Prior to the PXRD measurements, all samples were ground and soaked in water to reproducibly obtain the water‐rich *large*‐*pore* form of the MIL‐53 type compounds. Figure 4 shows the PXRD data of four different samples of Al‐MIL‐53‐NO_2_ with four different particle sizes (d ≈ 550 nm – 125 µm), which were used in the physisorption study (see next section). Le Bail fits against the PXRD data (Figure S3.3–S3.10) were carried out for all compounds to confirm the phase purity and to determine and compare the lattice parameters with literature data (Table S3.3–S3.10). Al‐MIL‐53‐X (X = ‐OH, ‐Br, ‐COOH, ‐NO_2_), Al‐MIL‐121, Al‐MIL‐118A, and Al‐MIL‐96 were obtained phase‐pure, while Al‐MIL‐53‐H could not be obtained phase‐pure as indicated by additional reflections in the PXRD data which can be attributed to recrystallized linker (H_2_BDC). The crystal structures of Al‐MIL‐53‐NO₂, Al‐MIL‐53‐Br, and Al‐MIL‐53‐COOH were not yet known from literature. Thus, SCXRD measurements were carried out for crystals of these compounds. While the crystals of Al‐MIL‐53‐NO_2_ and Al‐MIL‐53‐Br were suitable for SCXRD, both compounds exhibited significant disorder, as previously observed for Al‐MIL‐53‐NO_2_ and other MIL‐53‐type compounds.^[^ ^39^, ^61^ ^]^ Due to the high degree of disorder and the low occupancy of the ‐NO₂ and ‐Br groups, it was not possible to determine the crystal structures of Al‐MIL‐53‐NO₂ and Al‐MIL‐53‐Br sufficiently (see Section S3 for further details). In contrast, a lower degree of disorder of the phenyl ring and the free carboxylic acids, presumably due to the formation of hydrogen (Figure S3.2d), was found for Al‐MIL‐53‐COOH. Thus, the crystal structure of the water‐rich *large*‐*pore* form of Al‐MIL‐53‐COOH containing water molecules (*lp*‐H_2_O) was elucidated by SCXRD, and phase purity of the bulk material was confirmed by Rietveld refinement against PXRD data (Section S3.1). Prior to the measurement, the sample was humidified with water to obtain the water‐rich *large‐pore* form of the Al‐MOF with MIL‐53 topology (Al‐MIL‐53‐COOH_*lp*_H_2_O). The structural data for Al‐MIL‐53‐COOH_*lp*‐H_2_O obtained from Rietveld refinement against PXRD data and from SCXRD data have been deposited with the Cambridge Crystallographic Data Center (CCDC‐number 2432421–2432422). The water‐rich *large‐pore* form contains four water molecules per formula unit ([Al(OH) (C_9_H_4_O_6_)] · 4 H_2_O) and the free carboxylic acid group of the linker can be found disordered in four equivalent positions with an occupancy of ¼.

Infrared (IR) spectroscopy confirms the presence of coordinating carboxylate groups and bridging *μ*‐OH groups in the different Al‐MOFs, as indicated by their characteristic vibrational bands (Section S4). For some Al‐MOFs, additional characteristic vibrational bands are observed, indicating the presence of noncoordinating − COOH (*ν* ≈ 1700 cm^−1^) groups attributed to linker molecules in the pores, as known for the *as*‐*synthesized*‐form (*as*‐form) of MIL‐53 type compounds.^[^ ^49^ ^]^ For instance, vibrational bands corresponding to noncoordinating − COOH groups attributed to linker molecules in the pores were found for Al‐MIL‐53‐NO₂ (Ox‐1–1) synthesized using oxalic acid as the coordination modulator (Figure S4.2). In addition, digestion ^1^H‐ and ^13^C‐NMR spectra (Figure S4.3–S4.5) for samples synthesized with different ditopic and monotopic coordination modulators were recorded to confirm the integrity of the linker molecule and the absence of the coordination modulators in the final product. The bands in the NMR spectra were assigned to the linker molecule and residual solvents, using literature data confirming the absence of acetic acid, malonic acid, or oxalic acid in the final product.^[^ ^62^, ^63^ ^]^ Furthermore, the spectroscopic data obtained were used to calculate the sum formulas of the MOFs. The high degree of agreement between the calculated and measured decomposition steps in the thermogravimetric data (see Section S4.3) also confirm the absence of modulator species in the framework or missing linker defects.

#### 2.2.1 Particle Size Dependent Sorption Properties

**Figure 5:**
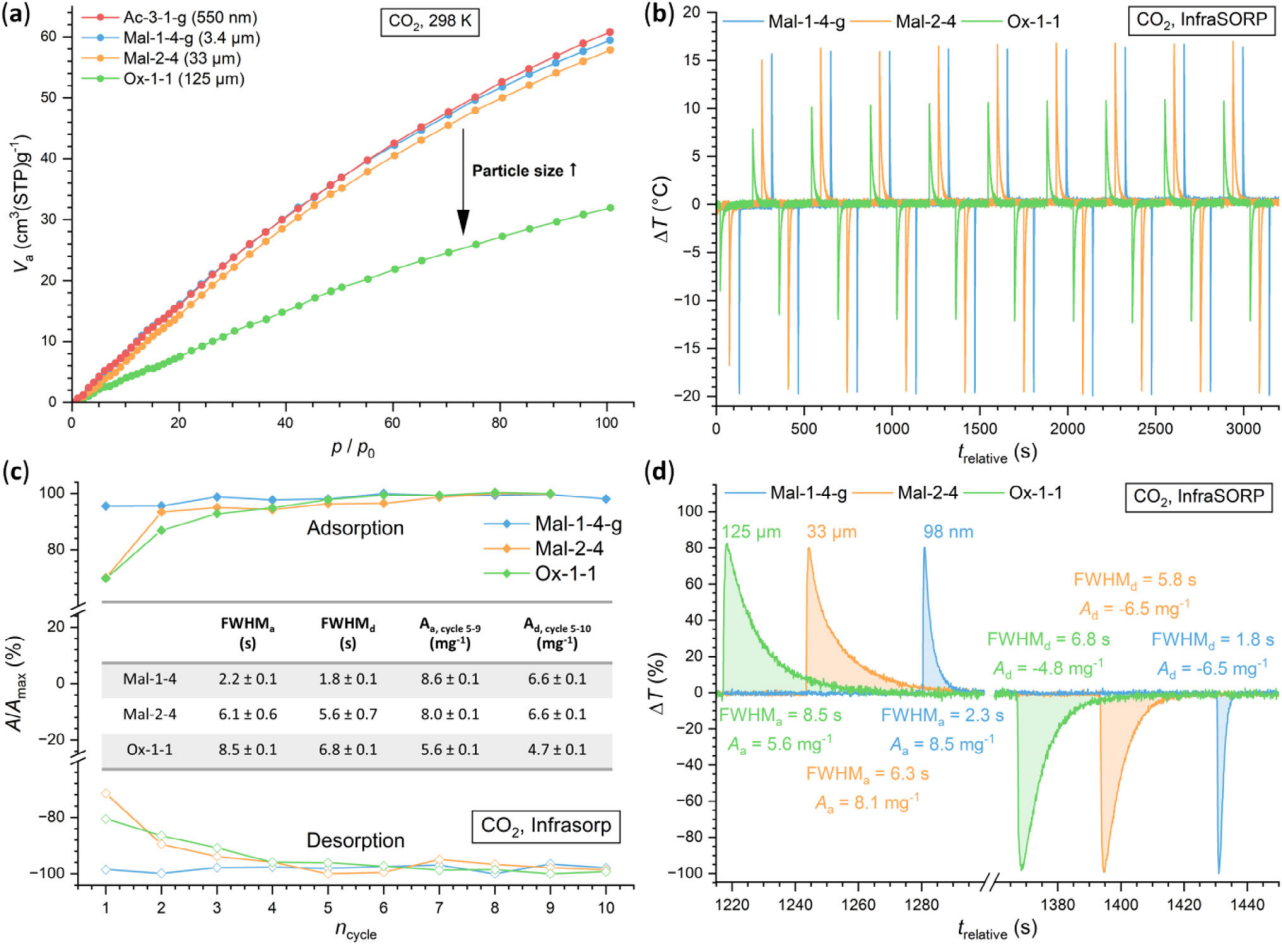
(**a**) CO_2_ sorption isotherms of Al‐MIL‐53‐NO_2_ with different particle sizes ((Ac‐3–1‐g (550 nm) > Mal‐1–4‐g (3.5 µm) > Mal‐2–4 (33 µm) > Ox‐1–1 (125 µm). The sorption measurements were carried out at 298 K (CO_2_) and the samples were previously activated for 3 hours at 120 °C under reduced pressure (p < 10^−2^ kPa). (**b**) – (**d**) Adsorption/desorption cycling data of Al‐MIL‐53‐NO_2_ with three different particle sizes (Mal‐1–4‐g (3.5 µm) > Mal‐2–4 (33 µm) > Ox‐1–1 (125 µm)) obtained by optical calorimetry (InfraSORP technology^[^ ^67^, ^68^ ^]^). (**b**) Temperature profiles during CO_2_ adsorption and desorption cycles (10 cycles). (**c**) Plot of the integrated peak areas *A*, normalized to the maximum area *A*_max_ and the mass *m* the of the sample, during the adsorption and desorption of CO_2_ over 10 sorption cycles. (**d**) Temperature profiles with integrated peak areas *A*, normalized to the maximum change in temperature and the mass *m* of the sample and FWHM (Full Width at Half Maximum) of the adsorption and desorption at cycle number four, when a stable signal is observed for all samples.

The strong influence of crystal size on the breathing behavior of MOFs is well‐documented, especially for MIL‐53 type MOFs.^[^ ^31^, ^64^, ^65^ ^]^ Theoretical calculations suggested faster switching kinetics for smaller crystallites of Al‐MIL‐53‐H,^[^ ^66^ ^]^ which was subsequently verified by Bon et al. using synchrotron time‐resolved PXRD and adsorption rate analysis of n‐butane physisorption.^[^ ^31^ ^]^ In addition, Diaz et al. reported a rigidification of Al‐MIL‐53‐H with decreasing crystal size to < 20 nm, leading to a loss of the breathing behavior of the MOF.^[^ ^64^ ^]^ With four different particle sizes of Al‐MIL‐53‐NO₂ (Ac‐3–1‐*g* (∼550 nm), Mal‐1–4‐*g* (∼3.4 µm), Mal‐2–4 (∼33 µm), and Ox‐1–1 (∼125 µm)) available, we studied the CO₂ adsorption and desorption properties at 298 K (Figure 5, Section S6) to investigate the influence of particle size on sorption properties (that is, uptake capacity and sorption kinetics). For the three samples containing the smallest particles, similar CO_2_ adsorption capacities in the range of 61 cm^3^/g (11.4 wt%, literature: 10.8 wt%^[^ ^11^ ^]^) were observed. Only for the sample containing the largest particles (Ox‐1–1 (125 µm)) a lower adsorption capacity of 31 cm^3^/g (5.8 wt%) was observed, which can be attributed to the presence of noncoordinating linker molecules in the pores of Al‐MIL‐53‐NO_2_, leading to a reduced CO_2_ uptake capacity as proven by IR spectroscopy (Section S4.2–S4.3). To further investigate the cycle performance and to learn more about the kinetics of the CO_2_ adsorption and desorption process, we performed additional cyclic (10 cycles) sorption experiments at 298 K for Mal‐1–4‐g (3.4 µm), Mal‐2–4 (33 µm), and Ox‐1–1 (125 µm) by optical calorimetry using the InfraSORP technology, first presented by Kaskel et al in 2011.^[^ ^67^, ^68^, ^69^ ^]^ The InfraSORP uses an IR sensor to record the temperature changes of a sample during adsorption or desorption. An increase in temperature due to CO_2_ adsorption and a temperature decrease due to desorption under N_2_ flow takes place, which can be qualitatively and semi‐quantitatively evaluated. The shape and integral of the signal directly correlate with the adsorption capacity,^[^ ^67^, ^70^ ^]^ and the temperature profile can be used to extract information about the kinetics of the sorption process.^[^ ^67^, ^68^ ^]^

The results of the InfraSORP measurements using ∼ 22 mg of the respective sample are shown in Figure 5. During adsorption, a maximum increase in temperature of Δ*T* ≈ 15.5 ± 0.1 K (*T*_max_ = 41.5 °C, observed for Mal‐2–4) and during desorption, an endothermic cooling of the samples to Δ*T* ≈ −19.5 K± 0.2 K (*T*_min_ = 0.9 °C, observed for Mal‐1–4‐g) was observed (Figures 5b, S6.6–S6.8). For the smaller particles (Mal‐1–4‐g (3.4 µm)), the full adsorption capacity was already reached in the first adsorption/desorption cycle, while for Mal‐2–4 (33 µm) and Ox‐1–1 (125 µm) the full adsorption capacity was reached after the fourth cycle (Figure 5c). This can be explained by the fact that the samples cannot be thermally activated in the InfraSORP prior to the measurement. Water molecules in the pores are only successively remove from the pores and a stable signal indicates full CO_2_ adsorption capacity. Once the H_2_O molecules are desorbed, only minor fluctuations in the peak integrals with a standard deviation of Δ*A*_a_ = 0.1 mg^−1^ (Figure 5c) demonstrating the cyclic stability of the CO_2_ sorption of all three Al‐MIL‐53‐NO_2_ samples. In contrast, the temperature profiles differ with particle size. For the smallest particles (Mal‐1–4‐g), the base temperature (Δ*T* = 0 °C) is reached within 14 seconds after adsorption (FWHM_a_ = 2.2 ± 0.1 s). With increasing particle size (Mal‐2–4 and Ox‐1–1) the base temperature is reached after 50 seconds (FWHM_a_ = 6.1 ± 0.6 s) and 80 seconds (FWHM_a_ = 8.5 ± 0.1 s), respectively, (Figure 5d) and the same trend is also observed for the desorption processes. The difference in the absolute values between the adsorption and desorption rate (FWHM_adsorption_ > FWHM_desorption_) can be attributed to the difference in the CO_2_ and N_2_ gas flow rate and the pore‐opening and ‐closing behavior, as the contraction of the structure is faster compared to the reopening as previously described for MIL‐53 type MOFs.^[^ ^31^ ^]^ The correlation of the particle size with the adsorption kinetics highlights the fast sorption kinetics assessment using InfraSORP technology as a proof of concept. The determination of the details on the sorption mechanism would necessitate the use of in situ measurements in dedicated instruments together with PXRD or IR spectroscopy and will be part of upcoming studies.

## 3 Conclusion

In conclusion, HT investigations varying the type and amount of mono‐ and dicarboxylic acids as coordination modulators (acetic acid, malonic acid, oxalic acid) and NaOH as pH modulator, a synergistic effect on the crystal size of eight Al‐MOFs, particularly Al‐MIL‐53‐NO_2_, was demonstrated. The ability to vary both the type and concentration of coordination modulators, in combination with pH modulation, allowed the range of particle sizes to be extended compared to the use of only coordinating modulators. Thus, both large crystals (*d* ≤ 800 µm) and nanoparticles (*d* ≈ 100 nm) of Al‐MIL‐53‐NO_2_ were successfully synthesized by fine‐tuning the molar ratios of the modulators, demonstrating the flexibility and versatility of this approach. In the case of the oxalic acid modulated synthesis, relatively high reaction temperatures (*T* > 170 °C) were required to obtain highly crystalline products. In contrast, low reaction temperatures (*T* = 100 °C) resulted in both large crystals (≈ 100 µm) and nano‐ to microcrystalline powders (*d* ≥ 100 nm) when using acetic acid or malonic acid. A high modulator concentration in combination with higher NaOH concentrations resulted in the largest crystals of the Al‐MOFs, whereas high pH values (high NaOH concentration) in combination with low modulator concentrations and high heating rates resulted in micro‐ or nanocrystalline powders. Thus, the composition of the synthesis mixture has a significant influence on the crystallization kinetics and the final crystal size of the Al‐MOFs. The coordination modulator and the pH modulator affect nucleation and crystal growth. Adding a stoichiometric amount of NaOH leads to the rapid deprotonation of the linker, which promotes fast nucleation and results in small particles. The coordination modulator stabilizes these small crystals while gradually increasing the pH and protonating the remaining linker molecules. In contrast, an excess of the pH modulator (NaOH) leads a decrease in deprotonation rate and is thus limiting the nucleation and allows for faster crystal growth, leading to larger crystals. Further investigation to confirm the role of the pH and coordination modulator will be part of our future work employing time‐resolved in situ methods to follow the evolution of both the liquid and solid phases. The influence of the particle size on CO_2_ sorption properties was demonstrated for Al‐MIL‐53‐NO_2_ with four different particle sizes (100 nm – 125 µm) using InfraSORP technology. While only minor fluctuations in the adsorption capacity were found, large differences in the adsorption rates correlate with the different particle sizes (increasing adsorption rate with decreasing particle size: Mal‐1–4‐*g* (3.4 µm) > Mal‐2–4 (33 µm) > Ox‐1–1 (125 µm)).

## 4 Experimental Section

### HT Investigations

HT studies were performed using steel autoclaves with 24 Teflon inserts with a total volume of 2 mL each.^[^ ^71^, ^72^ ^]^ The molar ratios of the coordination modulators and the pH modulator NaOH were varied, while keeping the amount of AlCl_3_ and linker constant. Aqueous solutions of AlCl_3_, modulator, and NaOH were added to the Teflon insert containing the linker as a solid, and the reaction volume was kept constant at 0.8 mL by the addition of water. After homogenization of the reaction mixture, the autoclaves were sealed and the reaction vessels were placed in a Memmert UNB 500 oven with forced ventilation and a programmable temperature‐time program. The reaction products were separated by filtration using a HT filtration block, washed with 1 mL water and 1 mL ethanol each and dried at room temperature. Detailed information on the temperature‐time programs, the exact amounts or reactants employed and the characterization data of the reaction products obtained (optical micrographs, PXRD data) are given in Section S2.

### Optimized Synthesis Conditions

The following sections describe the general procedures for the synthesis of large crystals using oxalic acid as a coordination modulator and NaOH as a pH modulator for various Al‐MOFs (Al‐MIL‐53‐X (with X = ‐H, ‐NO_2_, ‐OH, ‐Br, ‐COOH, ‐(COOH)_2_ (MIL‐121)), Al‐MIL‐96, Al‐MIL‐118A, and [Al(OH) (1,4‐NDC)]) and the synthesis of small crystals of Al‐MIL‐53‐NO_2_. Detailed information on the temperature‐time programs, the exact amounts or reactants employed and reaction products obtained are given in Section S2 of the Supporting Information.

**Synthesis of large crystals**: In a 2 mL Teflon vial, the linker was mixed with of diluted oxalic acid (*c* = 0.72 mol/L), water, an aqueous solution of AlCl_3_ (*c* = 1.44 mol/L), and diluted NaOH solution (*c* = 6 mol/L). Subsequently the autoclave was sealed and the reaction was carried out in an oven using a temperature‐time program of 6hours‐30hours‐6 hours at elevated temperature. The reaction product was separated by filtration, washed with water (1 mL), and ethanol (1 mL) and dried at room temperature.

**Synthesis of small crystals**: In a 14 mL glass vial, 2‐nitroterephthalic acid (H_2_BDC‐NO_2_) was mixed with the coordination modulator (HAc, H_2_Mal), water, diluted NaOH solution (*c* = 2 mol/L), and an aqueous solution of AlCl_3_ (*c* = 1.44 mol/L). Subsequently the reaction vial was sealed and placed in preheated (100 °C) aluminum block. The reaction was carried out at 100 °C for 20 hours under stirring using a heating plate with magnetic stirrer. The reaction product was separated by centrifugation, washed with water (8 mL), and ethanol (8 mL) and dried at room temperature.

### Characterization Methods

**Powder and SCXRD**: The powder X‐ray diffraction data (PXRD) were collected in transmission geometry using a STOE Stadi MP or a STOE Stadi P‐Combi diffractometer equipped both with a MYTHEN 1 K detector and using monochromatic Cu‐Kα1 radiation. The HT PXRD measurements were performed using a xy‐stage. Topas Academic^[^ ^73^, ^74^ ^]^ was used for indexing and refinements.

The single crystal X‐ray data for Al‐MIL‐53‐COOH_*lp*_H_2_O were collected on a STOE IPDS‐2 diffractometer using Mo‐Kα radiation. The structure was solved using the program SHELXT^[^ ^75^ ^]^ and refined with SHELXL^[^ ^75^ ^]^ using Olex2^[^ ^76^ ^]^ as the graphical interface.

CCDC number 2432421–2432422 contain the supplementary crystallographic data of Al‐MIL‐53‐COOH_*lp*_H_2_O obtained from SCXRD (CCDC‐2432421) and a Rietveld refinement against PXRD data (CCDC‐2432422). These data can be obtained free charge from the Cambridge Crystallographic Data Centre via http://www.ccdc.cam.ac.uk/data_request/cif.

**Optical and Scanning Electron Microscopy**: For the optical microscopy, the samples obtained were dispersed in silicon oil and characterized using an Olympus BX50 microscope. The crystal size and morphology of the products were determined by scanning electron microscopy using a Hitachi SU8700.

**Dynamic Light Scattering**: Particles size measurements were carried out by dynamic light scattering (DLS) using a Delsa Nano C (Beckman Coulter) particle analyzer. Prior to the DLS measurements the samples were dispersed in ethanol under ultrasonication (5 minutes, VWR ultrasonic cleaner USC600D).

**IR and ^1^H‐NMR spectroscopy**: IR‐spectra of the title compounds were collected using a Bruker ALPHA‐FT‐IR A220/D‐01 with an ATR‐unit. ^1^H‐ and ^13^C‐NMR spectra were recorded at 298 K using a Bruker AvanceNeo 500‐MHz spectrometer.

**Thermogravimetric measurements**: Thermogravimetric measurements were performed on a Linseis STA PT 1000 (airflow = 6 dm^3^/h, heating rate = 8 K/min). The sample amount was approximately 25 mg for each sample.

**CO_2_ Gas Sorption Measurements**: CO_2_ sorption measurements at 298 K were carried out using a BEL Japan Inc. BELSORP MiniX. Prior to the measurements the samples were treated for 3 hours at elevated temperatures under reduced pressure (p < 10^−2^ kPa). To confirm the integrity and the long‐range order of the structures after thermal activation, PXRD patterns of the samples were collected after the sorption measurements.

**InfraSORP Technology**: Optical calorimetric measurements were performed using the InfraSORP technology^[^ ^67^, ^68^, ^69^ ^]^ with an attached sample changer. CO_2_ (Air Liquide, ≥ 99.9995 %) was used for adsorption and nitrogen (Linde, ≥ 99.9999 %)) for desorption. The flow rate was calibrated to nitrogen. The samples were placed in the sample holders at ambient conditions. Cyclic measurements (10 cycles per sample) were carried out with the following steps per cycle: 90 seconds of adsorption (200 cm^3^min^−1^ CO_2_ gas stream); 60 seconds bypass (changing the gas and purging the whole system with the new gas); 120 seconds of desorption (200 cm^3^min^−1^ N_2_ gas stream); 60 seconds bypass.

## Supporting Information

The Supporting Information includes further information on materials and methods used, synthesis details, HT investigations, crystal structure determination, and refinement, Le Bail fits, IR spectroscopy, DLS, ^1^H‐ and ^13^C‐NMR spectroscopy, thermogravimetric measurements, and sorption properties. The authors have cited additional references within the Supporting Information.^[^ ^39^, ^61^, ^62^, ^73^, ^74^, ^77^, ^78^, ^79^, ^80^, ^81^, ^82^, ^83^ ^]^

## Conflict of Interest

The authors declare no conflict of interest.

## Supporting information



Supporting Information

## Data Availability

The data that support the findings of this study are available in the supplementary material of this article.
