# Supplementary material for: From Nanoparticles to Single Crystals of Al‐MOFs: Synergistic Coordination and pH Modulation, and Rapid Sorption Kinetics Assessment by Optical Calorimetry
Source: Chemistry. 2025 Jun 10;31(38):e202501110. doi: 10.1002/chem.202501110 (PMC12238924; doi:10.1002/chem.202501110)
Supplement: Supplementary file 1 — Supporting Information [file CHEM-31-e202501110-s001.pdf]

Supporting Information

From Nanoparticles to Single Crystals of Al-MOFs: Synergistic Coordination and pH Modulation, and Rapid Sorption Kinetics Assessment by Optical Calorimetry

Bastian Achenbach,[a] Christoph Meier,[a] Norbert Stock*[a,b]

[a] B. Achenbach, C. Meier, Prof. Dr. N. Stock*
Institute of Inorganic Chemistry
Kiel University
Max-Eyth-Str. 2, 24118 Kiel, Germany
E-mail: Stock@ac.uni-kiel.de

[b] Prof. Dr. N. Stock
Kiel Nano, Surface and Interface Science KiNSIS
Kiel University
Christian-Albrechts-Platz 4, 24118 Kiel, Germany
E-mail: Stock@ac.uni-kiel.de

Table of Contents

Table of Contents 2
1. Materials and Optimized Synthesis Procedures 3
1.1. Chemicals 3
1.2. Optimized Synthesis Conditions 3
2. Synthesis Optimization 5
2.1. High-throughput (HT) Investigation 5
2.1.1. Screening of Different Mono- and Ditopic Modulators 6
2.1.2. Al-MIL-53-H (Linker: H_2BDC) 12
2.1.3. Al-MIL-53-OH (Linker: H_2BDC-OH) 14
2.1.4. Al-MIL-53-Br (Linker: H_2BDC-Br) 16
2.1.5. Al-MIL-53-COOH (Linker: H_2BDC-COOH) 18
2.1.6. Al-MIL-121, Al-MIL-118A, Al-MIL-120 (Linker: H_4BTEC) 20
2.1.7. Al-MIL-96 (Linker: H_3BTC) 22
2.1.8. Al(OH)(1,4-NDC) (Linker: 1,4-H_2NDC) 24
3. Crystal Structure Determination and Refinement 26
3.1. Single-Crystal X-ray Diffraction and Rietveld Refinement of Al-MIL-53-COOH 26
3.2. Le Bail plots and crystallographic data 29
4. Spectroscopic Characterization and Thermal Properties 37
4.1. IR Spectroscopy 37
4.2. NMR Spectroscopy 39
4.3. Thermogravimetric Measurements 41
5. Dynamic Light Scattering (DLS) 43
6. Sorption Properties 44
6.1. Volumetric CO_2 Sorption Measurements 44
6.2. InfraSORP Measurements (Optical Calorimetry) 47
7. References 49

1. Materials and Optimized Synthesis Procedures

1.1. Chemicals

Aluminum chloride hexahydrate ($AlCl_3 \cdot 6\,H_2O$, Alfa Aesar, 99 %), acetic acid (CH_3COOH, VWR, > 99%), 1,2,4,5-benzenetetracarboxylic acid (H_4BTEC, pyromellitic acid, $C_6H_2(CO_2H)_4$, Alfa Aesar, 96 %), 2-bromoterephthalic acid (H_2BDC-Br, $C_8H_5BrO_4$, Sigma-Aldrich, 95 %), ethanol (C_2H_5OH, Walter-CMP, > 98 %), 2-hydroxyterephthalic acid (H_2BDC-OH, $C_8H_6O_5$, TCI, > 98 %), malonic acid ($C_3H_4O_4$, Merck, 99 %), 1,4-naphthalenedicarboxylic acid (1,4-H_2NDC, $C_{12}H_8O_4$, TCI, > 95 %), 2-nitroterephthalic acid (H_2BDC-NO_2, $C_8H_7NO_6$, Sigma-Aldrich, > 99 %), oxalic acid dihydrate ($C_2H_2O_2 \cdot 2\,H_2O$, Merck, 99.5 %), sodium hydroxide (NaOH, Grüssing GmbH, 99 %), terephthalic acid (H_2BDC, $C_8H_6O_4$, abcr, 99 %), trimellitic anhydride ($C_9H_4O_5$, TCI, > 95%), trimesic acid (H_3BTC, $C_6H_3(CO_2H)_3$, Sigma-Aldrich, 95 %), were commercially obtained and used without further purification.

1.2. Optimized Synthesis Conditions

The following paragraphs describe the optimized synthesis conditions (temperature-time programs, reaction temperature and the exact amounts or reactants) for the synthesis of large crystals using oxalic acid as a coordination modulator and NaOH as a pH modulator for the following Al-MOFs: Al-MIL-53-X (with X = -H, -NO_2, -OH, -Br, -COOH, -$(COOH)_2$ (MIL-121)), Al-MIL-96, Al-MIL-118A and [Al(OH)(1,4-NDC)] and the synthesis of small crystals of Al-MIL-53-NO_2. The optimized synthesis conditions were obtained by the high-throughput (HT) investigations described in Section S2.

Optimized Synthesis Conditions for Large Crystals

Al-MIL-53 (Ox-1.5-4.0): In a 2 mL Teflon® vial, 23.9 mg (1 eq., 144 µmol) of terephthalic acid (H_2BDC) were mixed with 300 µL (1.5 eq., *c* = 0.72 mol/L, 216 µmol) of diluted oxalic acid, 304 µL of water, 100 µL of an aqueous solution of $AlCl_3$ (1 eq., *c* = 1.44 mol/L, 144 µmol) and 96 µL of diluted NaOH solution (4.0 eq., *c* = 6 mol/L, 576 µmol). Subsequently the autoclave was sealed and the reaction was carried out in an oven using a temperature-time program of 6h-30h-6h and a reaction temperature of 200 °C. The reaction product was separated by filtration, washed with water (1 mL) and ethanol (1 mL) and dried at room temperature.

Al-MIL-53-OH (Ox-3.0-2.0): In a 2 mL Teflon® vial, 26.2 mg (1 eq., 144 µmol) of 2-hydroxyterephthalic acid (H_2BDC-OH) were mixed with 600 µL (3.0 eq., *c* = 0.72 mol/L, 432 µmol) of diluted oxalic acid, µL of water, 100 µL of an aqueous solution of $AlCl_3$ (1 eq., *c* = 1.44 mol/L, 144 µmol) and 48 µL of diluted NaOH solution (2 eq., *c* = 6 mol/L, 288 µmol). Subsequently the autoclave was sealed and the reaction was carried out in an oven using a temperature-time program of 6h-30h-6h and a reaction temperature of 170 °C. The reaction product was separated by filtration, washed with water (1 mL) and ethanol (1 mL) and dried at room temperature.

Al-MIL-53-Br (Ox-3.0-4.0): In a 2 mL Teflon® vial, 35.3 mg (1 eq., 144 µmol) of 2-bromoterephthalic acid (H_2BDC-Br) were mixed with 600 µL (3 eq., *c* = 0.72 mol/L, 432 µmol) of diluted oxalic acid, 4 µL of water, 100 µL of an aqueous solution of $AlCl_3$ (1 eq., *c* = 1.44 mol/L, 144 µmol) and 96 µL of diluted NaOH solution (4 eq., *c* = 6 mol/L, 576 µmol). Subsequently the autoclave was sealed and the reaction was carried out in an oven using a temperature-time program of 6h-30h-6h and a reaction temperature of 210 °C. The reaction product was separated by filtration, washed with water (1 mL) and ethanol (1 mL) and dried at room temperature.

Al-MIL-53-NO_2 (Ox-3.0-6.0): In a 2 mL Teflon® vial, 15.2 mg (1.0 eq., 72 µmol) of 2-nitroterephthalic acid (H_2BDC-NO_2) were mixed with 300 µL (3 eq., *c* = 0.72 mol/L, 216 µmol) of diluted oxalic acid, 234 µL of water, 50 µL of an aqueous solution of $AlCl_3$ (1 eq., *c* = 1.44 mol/L, 72 µmol) and 216 µL of diluted NaOH solution (6 eq., *c* = 2 mol/L, 432 µmol). Subsequently the autoclave was sealed and the reaction was carried out in an oven using a temperature-time program of 6h-30h-6h and a reaction temperature of 170 °C. The reaction product was separated by filtration, washed with water (1 mL) and ethanol (1 mL) and dried at room temperature.

Al-MIL-53-COOH (Ox-2.5-2.0): In a 2 mL Teflon® vial, 27.7 mg (1 eq., 144 µmol) of trimellitic anhydride (H_2BDC-COOH) were mixed with 500 µL (2.5 eq., *c* = 0.72 mol/L, 360 µmol) of diluted oxalic acid, 152 µL of water, 100 µL of an aqueous solution of $AlCl_3$ (1 eq., *c* = 1.44 mol/L, 144 µmol) and 48 µL of diluted NaOH solution (2 eq., *c* = 6 mol/L, 288 µmol). Subsequently the autoclave was sealed and the reaction was carried out in an oven using a temperature-time program

of 6h-30h-6h and a reaction temperature of 170 °C. The reaction product was separated by filtration, washed with water (1 mL) and ethanol (1 mL) and dried at room temperature.

Al-MIL-121 (Ox-3.0-0.0): In a 2 mL Teflon® vial, 65.0 mg (0.5 eq., 256 µmol) of 1,2,4,5-benzenetetracarboxylic acid (H_4BTEC) were mixed with 193.6 mg (3 eq., 1.54 mmol) of diluted oxalic acid, 443 µL of water and 357 µL of an aqueous solution of $AlCl_3$ (1 eq., c = 1.44 mol/L, 512 µmol). Subsequently the autoclave was sealed and the reaction was carried out in an oven using a temperature-time program of 6h-30h-6h and a reaction temperature of 200 °C. The reaction product was separated by filtration, washed with water (1 mL) and ethanol (1 mL) and dried at room temperature.

Al-MIL-118A (Ox-2.5-2.0): In a 2 mL Teflon® vial, 65.0 mg (0.5 eq., 256 µmol) of 1,2,4,5-benzenetetracarboxylic acid (H_4BTEC) were mixed with 161.4 mg (2.5 eq., c = 0.72 mol/L, 1.28 mmol) of diluted oxalic acid, 273 µL of water, 357 µL of an aqueous solution of $AlCl_3$ (1 eq., c = 1.44 mol/L, 512 µmol) and 171 µL of diluted NaOH solution (2 eq., c = 6 mol/L, 1.02 mmol). Subsequently the autoclave was sealed and the reaction was carried out in an oven using a temperature-time program of 6h-30h-6h and a reaction temperature of 200 °C. The reaction product was separated by filtration, washed with water (1 mL) and ethanol (1 mL) and dried at room temperature.

Al-MIL-96 (Ox-1.5-0.0): In a 2 mL Teflon® vial, 15.1 mg (0.5 eq., 72.0 µmol) of trimesic acid (H_3BTC) were mixed with 300 µL (1.5 eq., c = 0.72 mol/L, 216 µmol) of diluted oxalic acid, 400 µL of water and 100 µL of an aqueous solution of $AlCl_3$ (1 eq., c = 1.44 mol/L, 144 µmol). Subsequently the autoclave was sealed and the reaction was carried out in an oven using a temperature-time program of 6h-30h-6h and a reaction temperature of 200 °C. The reaction product was separated by filtration, washed with water (1 mL) and ethanol (1 mL) and dried at room temperature.

Al(OH)(1,4-NDC) (Ox-3.0-2.0): In a 2 mL Teflon® vial, 7.8 mg (0.5 eq., 36.0 µmol) of 1,4-naphthalenedicarboxylic acid (1,4-H_2NDC) were mixed with 300 µL (3 eq., c = 0.72 mol/L, 216 µmol) of diluted oxalic acid, 378 µL of water, 50 µL of an aqueous solution of $AlCl_3$ (1 eq., c = 1.44 mol/L, 72 µmol) and 72 µL of diluted NaOH solution (2 eq., c = 2 mol/L, 144 µmol). Subsequently the autoclave was sealed and the reaction was carried out in an oven using a temperature-time program of 6h-30h-6h and a reaction temperature of 170 °C. The reaction product was separated by filtration, washed with water (1 mL) and ethanol (1 mL) and dried at room temperature.

Optimized Synthesis Conditions for Small Crystals

Al-MIL-53-NO_2 (Ac-1-1-g): In a 14 mL glass vial, 304 mg (1.0 eq., 1.44 mmol) of 2-nitroterephthalic acid (H_2BDC-NO_2) were mixed with 250 µL (1.0 eq., c = 5.76 mol/L, 1.44 mmol) of diluted acetic acid, 6030 µL of water, 720 µL of diluted NaOH solution (1.0 eq., c = 2 mol/L, 1.44 mmol) and 1000 µL of an aqueous solution of $AlCl_3$ (1 eq., c = 1.44 mol/L, 1.44 mmol). Subsequently the reaction vial was sealed and placed in preheated (100 °C) aluminum block. The reaction was carried out at 100 °C for 20 h under stirring using a heating plate with magnetic stirrer. The reaction product was separated by centrifugation, washed with water (8 mL) and ethanol (8 mL) and dried at room temperature.

Al-MIL-53-NO_2 (Ac-1-3-g): In a 14 mL glass vial, 304 mg (1.0 eq., 1.44 mmol) of 2-nitroterephthalic acid (H_2BDC-NO_2) were mixed with 250 µL (1.0 eq., c = 5.76 mol/L, 1.44 mmol) of diluted acetic acid, 4590 µL of water, 2160 µL of diluted NaOH solution (3.0 eq., c = 2 mol/L, 4.32 mmol) and 1000 µL of an aqueous solution of $AlCl_3$ (1 eq., c = 1.44 mol/L, 1.44 mmol). Subsequently the reaction vial was sealed and placed in preheated (100 °C) aluminum block. The reaction was carried out at 100 °C for 20 h under stirring using a heating plate with magnetic stirrer. The reaction product was separated by centrifugation, washed with water (8 mL) and ethanol (8 mL) and dried at room temperature.

Al-MIL-53-NO_2 (Ac-3-1-g): In a 14 mL glass vial, 304 mg (1.0 eq., 1.44 mmol) of 2-nitroterephthalic acid (H_2BDC-NO_2) were mixed with 750 µL (1.0 eq., c = 5.76 mol/L, 4.32 mmol) of diluted acetic acid, 5530 µL of water, 720 µL of diluted NaOH solution (1.0 eq., c = 2 mol/L, 1.44 mmol) and 1000 µL of an aqueous solution of $AlCl_3$ (1 eq., c = 1.44 mol/L, 1.44 mmol). Subsequently the reaction vial was sealed and placed in preheated (100 °C) aluminum block. The reaction was carried out at 100 °C for 20 h under stirring using a heating plate with magnetic stirrer. The reaction product was separated by centrifugation, washed with water (8 mL) and ethanol (8 mL) and dried at room temperature.

Al-MIL-53-NO_2 (Mal-1-4-g): In a 14 mL glass vial, 304 mg (1.0 eq., 1.44 mmol) of 2-nitroterephthalic acid (H_2BDC-NO_2) were mixed with 1000 µL (1.0 eq., c = 1.44 mol/L, 1.44 mmol) of diluted malonic acid, 3120 µL of water, 2880 µL of diluted NaOH solution (1.0 eq., c = 2 mol/L, 5.76 mmol) and 1000 µL of an aqueous solution of $AlCl_3$ (1 eq., c = 1.44 mol/L, 1.44 mmol). Subsequently the reaction vial was sealed and placed in preheated (100 °C) aluminum block.

The reaction was carried out at 100 °C for 20 h under stirring using a heating plate with magnetic stirrer. The reaction product was separated by centrifugation, washed with water (8 mL) and ethanol (8 mL) and dried at room temperature.

2. Synthesis Optimization

2.1. High-throughput (HT) Investigation

The high-throughput investigation to study the effects of different mono- and ditopic coordination modulators (acetic acid, malonic acid and oxalic acid) on the crystal size and morphology of different Al-MOFs was carried using custom-made steel autoclaves[1] with 24 Teflon® inserts with a total volume of 2 mL each. The molar ratios were varied between 1.0 – 3.0 equivalents for the coordination modulators and 0.0 - 6.0 for NaOH as the pH modulator, respectively, while keeping the amounts and molar ratio of $AlCl_3$ and linker constant, i.e. 1 : 1 (1 equivalent corresponds to 0.144 mmol). Aqueous solutions of $AlCl_3$, modulator and NaOH were mixed with the linker, and the reaction volume was kept constant at 0.8 mL by the addition of water. The reactant solutions and solids were added to the reaction vessels in the following order:

1) Linker
2) Modulator (c_{HAc} = 5.76 mol/L, c_{H2Mal} = 1.44 mol/L, c_{H2Ox} = 0.72 mol/L)
3) $AlCl_3$ (c = 1.44 mol/L)
4) H_2O
5) NaOH (c_1 = 2 mol/L , c_2 = 6 mol/L, c_3 = 12 mol/L)

After sealing the autoclaves, the reaction vessels were placed in a Memmert UNB 500 oven with forced ventilation and a programmable temperature-time-program. The reaction products were separated by filtration using a HT filtration block, washed with 1 mL water and 1 mL ethanol and dried at room temperature. The reaction products were characterized using High-Throughput PXRD (Figure S2.1 – S2.18) and crystalline products were identified using literature data.

2.1.1. Screening of Different Mono- and Ditopic Modulators

The effect of the mono- and ditopic coordination modulators acetic acid (Table S2.1, Figure S2.1), malonic acid (Table S2.2, Figure S2.2) and oxalic acid (Table S2.3, Figure S2.3) on the crystal size and morphology of Al-MIL-53-NO_2 was investigated by varying the molar ratios of the different coordination modulators and NaOH while keeping the $AlCl_3$ and linker ($H_2BDC-NO_2$) concentrations constant.

Table S2.1. List of starting materials employed in the systematic HT study of the chemical system $AlCl_3$ / **$H_2BDC-NO_2$ / acetic acid (HAc)** / NaOH in water by varying the molar ratios of acetic acid and NaOH. The reaction temperature was set to 100 °C and a heating program of 1-18-1 h (t_1-t_2-t_3) was used. One equivalent corresponds to 0.144 mmol.

Synthesis-No.	Molar ratios (eq.)				$V_{solution}$ (µL) / ***m*** (mg)					Reaction product(s)
	$AlCl_3$	$H_2BDC-NO_2$	HAc	NaOH	$AlCl_3$[a] (µL)	$H_2BDC-NO_2$ (mg)	HAc[b] (µL)	NaOH (µL)	H_2O (µL)	
**Al-MIL-53-NO_2** (Ac-1.0-0.0)			1.0				25		675	$H_2BDC-NO_2$, Al-MIL-53-NO_2
(Ac-2.0-0.0)			2.0	0.0			50	/	650	$H_2BDC-NO_2$, Al-MIL-53-NO_2
(Ac-3.0-0.0)			3.0				75		625	$H_2BDC-NO_2$, Al-MIL-53-NO_2
(Ac-1.0-1.0)			1.0				25		603	Al-MIL-53-NO_2
(Ac-2.0-1.0)			2.0	1.0			50	72[d]	578	Al-MIL-53-NO_2
(Ac -3.0-1.0)			3.0				75		553	Al-MIL-53-NO_2
(Ac-1.0-2.0)			1.0				25		531	Al-MIL-53-NO_2
(Ac-2.0-2.0)			2.0	2.0			50	144[d]	506	Al-MIL-53-NO_2
(Ac-3.0-2.0)			3.0				75		481	Al-MIL-53-NO_2
(Ac-1.0-3.0)			1.0				25		459	Al-MIL-53-NO_2
(Ac-2.0-3.0)	1	1.0	2.0	3.0	100	30.4	50	216[d]	434	Al-MIL-53-NO_2
(Ac-3.0-3.0)			3.0				75		409	Al-MIL-53-NO_2
(Ac-1.0-4.0)			1.0				25		387	Al-MIL-53-NO_2, $AlO_x(O)_y$
(Ac-2.0-4.0)			2.0	4.0			50	288[d]	362	Al-MIL-53-NO_2
(Ac-3.0-4.0)			3.0				75		337	Al-MIL-53-NO_2
(Ac-1.0-5.0)			1.0				25		315	Al-MIL-53-NO_2, CAU-55-Cl[2]
(Ac-2.0-5.0)			2.0	5.0			50	360[d]	290	Al-MIL-53-NO_2, $AlO_x(O)_y$
(Ac-3.0-5.0)			3.0				75		265	Al-MIL-53-NO_2, $AlO_x(O)_y$
(Ac-1.0-6.0)			1.0				25		243	$AlO_x(O)_y$
(Ac-2.0-6.0)			2.0	6.0			50	432[d]	218	$AlO_x(O)_y$
(Ac -3.0-6.0)			3.0				75		193	$AlO_x(O)_y$

[a] c_{AlCl3} = 1.44 mol/L; [b] c_{HAc} = 5.76 mol/L; [c] c_{NaOH} = 6.0 mol/L; [d] c_{NaOH} = 2.0 mol/L

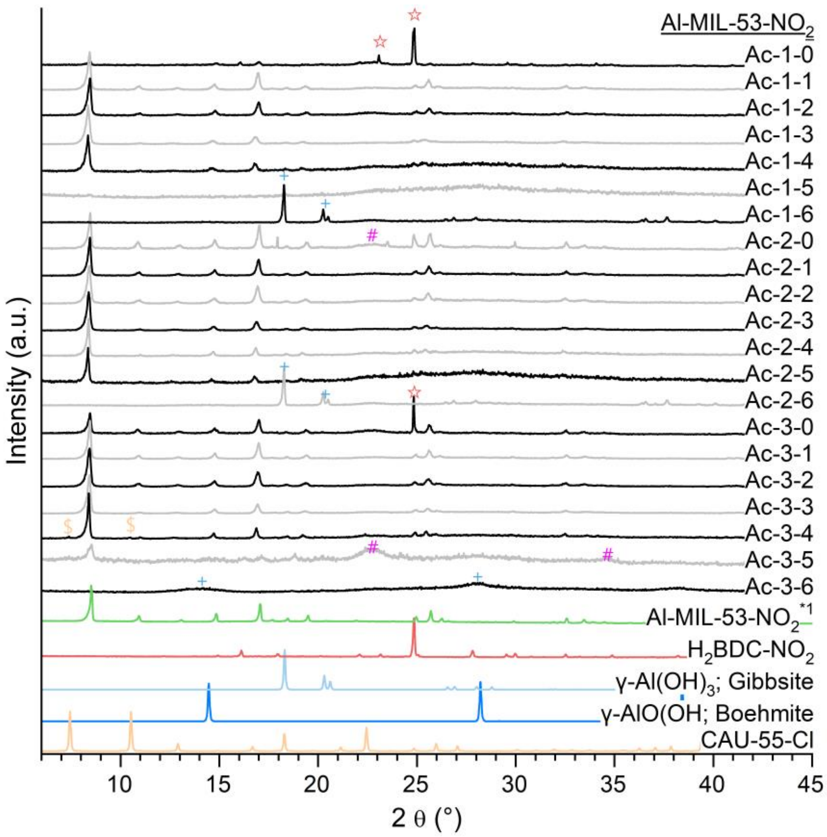

Figure S2.1. Measured PXRD patterns of the samples obtained in the HT-screening using nitroterephthalic acid as the linker, acetic acid (HAc) as the coordination modulator and NaOH as the pH modulator. To increase reproducibility and comparability, all samples were soaked with water prior to measurement to obtain the water-rich, *large-pore* form of Al-MIL-53-NO_2_*lp*_H_2O. For comparison, a measured PXRD pattern of the linker (H_2BDC-NO_2) and calculated PXRD patterns of different polymorphs of $AlO_x(OH)_y$ (Boehmite (ICSD: 27865) and Gibbsite (ICSD: 36233))[3] are shown. *1In Addition, the PXRD data of Al-MIL-53-NO_2_*lP*_H_2O used to confirm the phase purity (Section S3) is shown, which was ground prior to measurement to avoid additional scattering effects due to the presence of large crystals. *The broad band at 21 – 24 °2θ is related to the presence of filtration paper used to prepare the samples, ☆ highlight reflections corresponding to recrystallized linker, $ highlight reflections corresponding to CAU-55-Cl, a porous salt of composition $[Al_{24}(OH)_{56}(O_2CCH_3)_{12}]Cl_4$[2], and + mark reflections corresponding to different polymorphs of $AlO_x(OH)_y$.

Table S2.2. List of starting materials employed in the systematic HT study of the chemical system $AlCl_3$ / **H_2BDC-NO_2** / **malonic acid (H_2Mal)** / NaOH in water by varying the molar ratios of malonic acid and NaOH. The reaction temperature was set to 100 °C and a heating program of 1-18-1 h (*t*$_1$-*t*$_2$-*t*$_3$) was used. One equivalent corresponds to 0.144 mmol.

**Synthesis-No.**	**Molar ratios (eq.)**				**$V_{solution}$ (µL) / *m* (mg)**					**Reaction product(s)**
	**$AlCl_3$**	**H_2BDC-NO_2**	**H_2Mal**	**NaOH**	**$AlCl_3$[a] (µL)**	**H_2BDC-NO_2 (mg)**	**H_2Mal[b] (µL)**	**NaOH (µL)**	**H_2O (µL)**	
**Al-MIL-53-NO_2** (Mal-1.0-0.0)			1.0				100		528	no precipitate
(Mal-2.0-0.0)			2.0	0.0			200	/	428	no precipitate
(Mal-3.0-0.0)			3.0				300		328	no precipitate
(Mal-1.0-1.0)			1.0				100		528	Al-MIL-53-NO_2
(Mal-2.0-1.0)			2.0	1.0			200	72[d]	428	no precipitate
(Mal -3.0-1.0)			3.0				300		328	no precipitate
(Mal-1.0-2.0)			1.0				100		456	Al-MIL-53-NO_2
(Mal-2.0-2.0)			2.0	2.0			200	144[d]	356	Al-MIL-53-NO_2
(Mal-3.0-2.0)			3.0				300		256	no precipitate
(Mal-1.0-3.0)			1.0				100		384	Al-MIL-53-NO_2
(Mal-2.0-3.0)	1	1.0	2.0	3.0	100	30.4	200	216[d]	284	Al-MIL-53-NO_2
(Mal-3.0-3.0)			3.0				300		184	Al-MIL-53-NO_2
(Mal-1.0-4.0)			1.0				100		312	Al-MIL-53-NO_2
(Mal-2.0-4.0)			2.0	4.0			200	288[d]	212	Al-MIL-53-NO_2
(Mal-3.0-4.0)			3.0				300		112	no precipitate
(Mal-1.0-5.0)			1.0				100		240	Al-MIL-53-NO_2
(Mal-2.0-5.0)			2.0	5.0			200	360[d]	140	Al-MIL-53-NO_2, $AlO_x(O)_y$
(Mal-3.0-5.0)			3.0				300		40	no precipitate
(Mal-1.0-6.0)			1.0				100	432[d]	168	$AlO_x(O)_y$
(Mal-2.0-6.0)			2.0	6.0			200		68	$AlO_x(O)_y$
(Mal-3.0-6.0)			3.0				300	144[c]	256	no precipitate

[a] c_{AlCl3} = 1.44 mol/L; [b] c_{H2Mal} = 1.44 mol/L; [c] c_{NaOH} = 6.0 mol/L; [d] c_{NaOH} = 2.0 mol/L

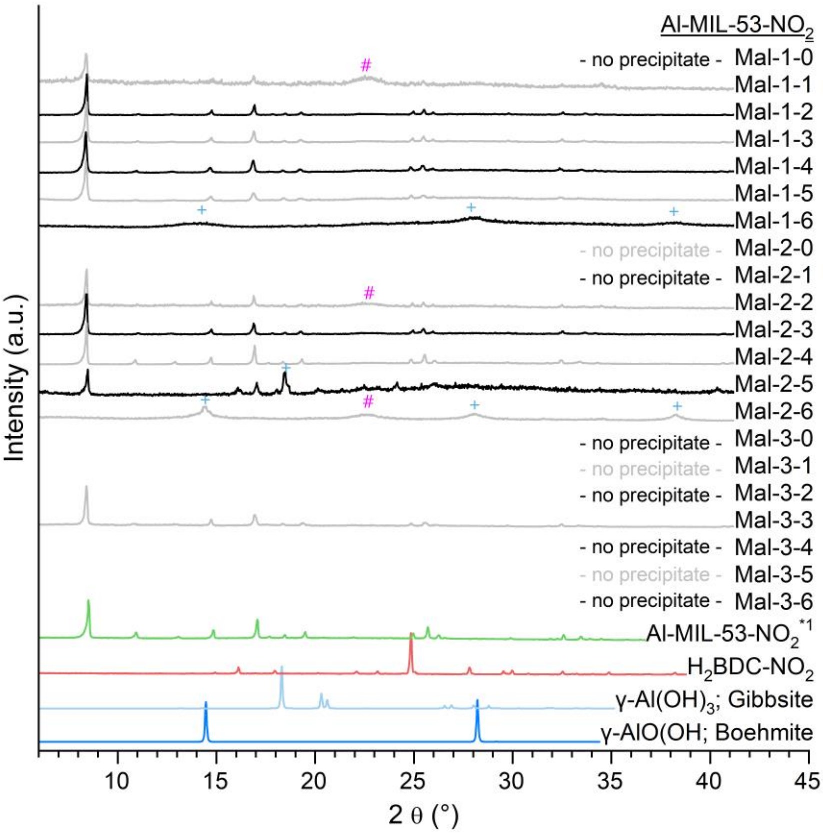

Figure S2.2. Measured PXRD patterns of the samples obtained in the HT-screening using nitroterephthalic acid as the linker, malonic acid (HAc) as the coordination modulator and NaOH as the pH modulator. To increase reproducibility and comparability, all samples were soaked with water prior to measurement to obtain the water-rich, *large-pore* form of Al-MIL-53-NO_2_*lp*_H_2O. For comparison, a measured PXRD pattern of the linker (H_2BDC-NO_2) and calculated PXRD patterns of different polymorphs of $AlO_x(OH)_y$ (Boehmite (ICSD: 27865) and Gibbsite (ICSD: 36233))[3] are shown. *1In Addition, the PXRD data of Al-MIL-53-NO_2_*lP*_H_2O used to confirm the phase purity (Section S3) is shown, which was ground prior to measurement to avoid additional scattering effects due to the presence of large crystals. ☆The broad band at 21 – 24 °2θ is related to the presence of filtration paper used to prepare the samples, ☆ highlight reflections corresponding to recrystallized linker and + mark reflections corresponding to different polymorphs of $AlO_x(OH)_y$.

Table S2.3. List of starting materials employed in the systematic HT study of the chemical system $AlCl_3$ / **H_2BDC-NO_2** / **oxalic acid (H_2Ox)** / NaOH in water by varying the molar ratios of oxalic acid and NaOH. The reaction temperature was set to 170 °C and a heating program of 6-30-6 h (t_1-t_2-t_3) was used. One equivalent corresponds to 0.072 mmol.

**Synthesis-No.**	**Molar ratios (eq.)**				**$V_{solution}$ (µL) / *m* (mg)**					**Reaction product(s)**
	**$AlCl_3$**	**H_2BDC-NO_2**	**H_2Ox**	**NaOH**	**$AlCl_3$[a] (µL)**	**H_2BDC-NO_2 (mg)**	**H_2Ox[b] (µL / mg)**	**NaOH (µL)**	**H_2O (µL)**	
**Al-MIL-53-NO_2** (Ox-1.0-0.0)			1.0				100 µL		650	H_2BDC-NO_2
(Ox-1.5-0.0)			1.5				150 µL		600	H_2BDC-NO_2
(Ox-2.0-0.0)			2.0	0.0			200 µL	/	550	no precipitate
(Ox-2.5-0.0)			2.5				250 µL		500	no precipitate
(Ox-3.0-0.0)			3.0				300 µL		450	no precipitate
(Ox-1.0-2.0)			1.0				100 µL		614	Al-MIL-53-NO_2
(Ox-2.0-1.0)			2.0	1.0			100 µL	36 µL	564	Al-MIL-53-NO_2
(Ox-3.0-1.0)			3.0				200 µL		514	Al-MIL-53-NO_2
(Ox-1.0-2.0)			1.0				300 µL		578	Al-MIL-53-NO_2
(Ox-1.5-2.0)			1.5				150 µL		528	Al-MIL-53-NO_2
(Ox-2.0-2.0)			2.0	2.0			200 µL	72[d]	478	Al-MIL-53-NO_2
(Ox-2.5-2.0)	1	1.0	2.5		50	15.2	250 µL		428	Al-MIL-53-NO_2
(Ox-3.0-2.0)			3.0				300 µL		378	Al-MIL-53-NO_2
(Ox-1.0-4.0)			1.0				100 µL		506	Al-MIL-53-NO_2
(Ox-1.5-4.0)			1.5				150 µL		456	Al-MIL-53-NO_2
(Ox-2.0-4.0)			2.0	4.0			200 µL	144[d]	406	Al-MIL-53-NO_2
(Ox-2.5-4.0)			2.5				250 µL		356	Al-MIL-53-NO_2
(Ox-3.0-4.0)			3.0				300 µL		306	Al-MIL-53-NO_2
(Ox-1.0-6.0)			1.0				100 µL		444	no precipitate
(Ox-1.5-6.0)			1.5				150 µL		384	Al-MIL-53-NO_2
(Ox-2.0-6.0)			2.0	6.0			200 µL	216[d]	334	Al-MIL-53-NO_2
(Ox-2.5-6.0)			2.5				250 µL		284	Al-MIL-53-NO_2
(Ox-3.0-6.0)			3.0				300 µL		234	Al-MIL-53-NO_2

[a] c_{AlCl3} = 1.44 mol/L; [b] c_{H2Ox} = 0.72 mol/L; [c] c_{NaOH} = 6.0 mol/L; [d] c_{NaOH} = 2.0 mol/L

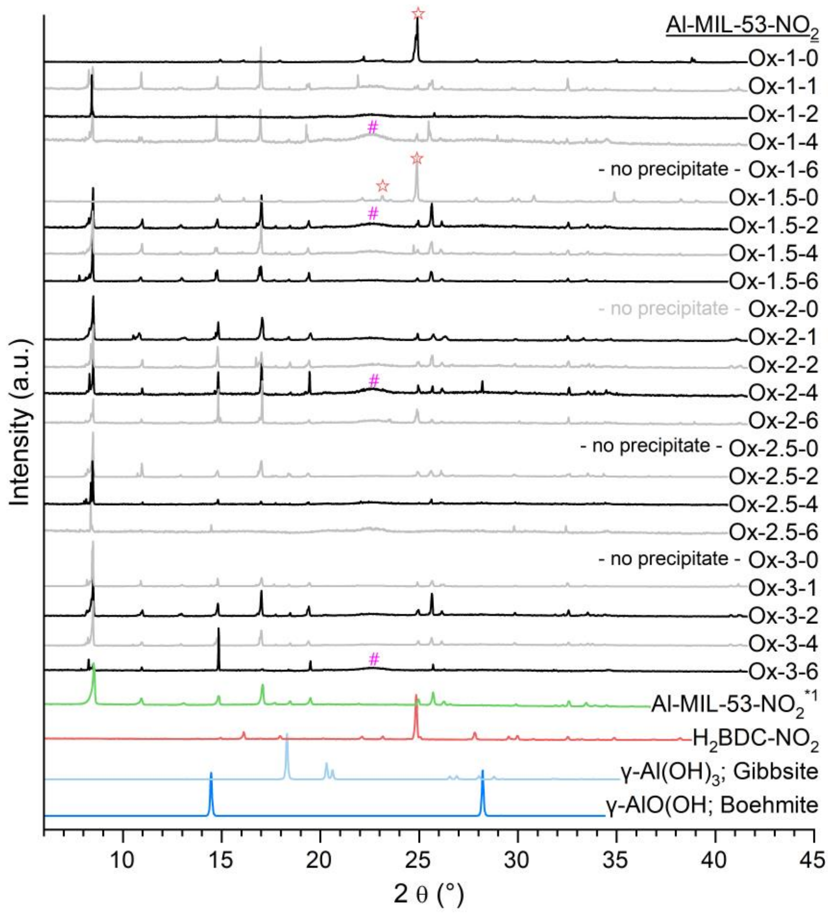

Figure S2.3. Measured PXRD patterns of the samples obtained in the HT-screening using nitroterephthalic acid as the linker, oxalic acid (HOx) as the coordination modulator and NaOH as the pH modulator. To increase reproducibility and comparability, all samples were soaked with water prior to measurement to obtain the water-rich, *large-pore* form of Al-MIL-53-NO_2_*lp*_H_2O. For comparison, a measured PXRD pattern of the linker (H_2BDC-NO_2) and calculated PXRD patterns of different polymorphs of $AlO_x(OH)_y$ (Boehmite (ICSD: 27865) and Gibbsite (ICSD: 36233))[3] are shown. *1In Addition, the PXRD data of Al-MIL-53-NO_2_*lP*_H_2O used to confirm the phase purity (Section S3) is shown, which was ground prior to measurement to avoid additional scattering effects due to the presence of large crystals. ☆The broad band at 21 – 24 °2θ is related to the presence of filtration paper used to prepare the samples, ☆ highlight reflections corresponding to recrystallized linker and + mark reflections corresponding to different polymorphs of $AlO_x(OH)_y$.

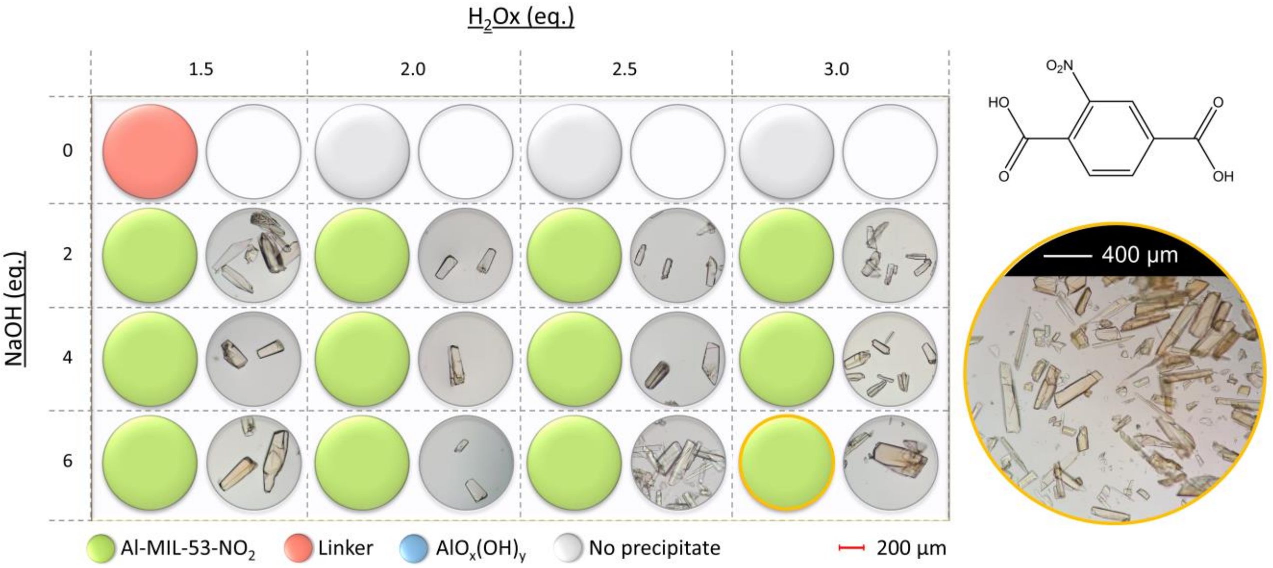

Figure S2.4. Crystallization diagram of the crystalline phases observed in the chemical system $AlCl_3$ / H_2BDC-NO_2 / oxalic acid / NaOH in H_2O for reactions carried out at 210 °C with different molar ratios of oxalic acid and NaOH, keeping the amount of $AlCl_3$ and H_2BDC-NO_2 constant. The crystalline phases observed are color-coded and the light grey circles mark the reactions where no precipitate was obtained. For each reaction that led to Al-MIL-53-NO_2, a microscopic image of the crystals is shown. In addition, for the optimized reaction condition (yellow circle), an optical micrograph with a higher resolution and a larger number of crystals is shown.

2.1.2. Al-MIL-53-H (Linker: H_2BDC)

Table S2.4. List of starting materials employed in the systematic HT study of the chemical system $AlCl_3$ / **H_2BDC** / oxalic acid (H_2Ox) / NaOH in water by varying the molar ratios of oxalic acid and NaOH. The reaction temperature was set to 200 °C and a heating program of 6-30-6 h (t_1-t_2-t_3) was used. One equivalent corresponds to 0.144 mmol.

Synthesis-No.	Molar ratios (eq.)				$V_{solution}$ (µL) / *m* (mg)					Reaction product(s)
	$AlCl_3$	H_2BDC	H_2Ox	NaOH	$AlCl_3$[a] (µL)	H_2BDC (mg)	H_2Ox[b] (µL / mg)	NaOH (µL)	H_2O (µL)	
**Al-MIL-53** (Ox-1.5-0.0)	1	1	1.5	0.0	100	23.9	300 µL	/	400	Al-MIL-53
(Ox-2.0-0.0)			2.0				400 µL		300	Al-MIL-53
(Ox-2.5-0.0)			2.5				500 µL		200	Al-MIL-53
(Ox-3.0-0.0)			3.0				600 µL		100	Al-MIL-53
(Ox-1.5-2.0)			1.5	2.0			300 µL	48[c]	352	Al-MIL-53
(Ox-2.0-2.0)			2.0				400 µL		252	Al-MIL-53
(Ox-2.5-2.0)			2.5				500 µL		152	Al-MIL-53
(Ox-3.0-2.0)			3.0				600 µL		52	Al-MIL-53
(Ox-1.5-4.0)			1.5	4.0			300 µL	96[c]	304	Al-MIL-53
(Ox-2.0-4.0)			2.0				400 µL		204	Al-MIL-53
(Ox-2.5-4.0)			2.5				500 µL		104	Al-MIL-53
(Ox-3.0-4.0)			3.0				600 µL		4	Al-MIL-53
(Ox-1.5-6.0)			1.5	6.0			300 µL	144[c]	256	Al-MIL-53, $AlO_x(OH)_y$
(Ox-2.0-6.0)			2.0				400 µL		156	Al-MIL-53, $AlO_x(OH)_y$
(Ox-2.5-6.0)			2.5				500 µL		56	Al-MIL-53
(Ox-3.0-6.0)			3.0				54.5 mg		556	Al-MIL-53

[a] c_{AlCl3} = 1.44 mol/L; [b] c_{H2Ox} = 0.72 mol/L; [c] c_{NaOH} = 6.0 mol/L

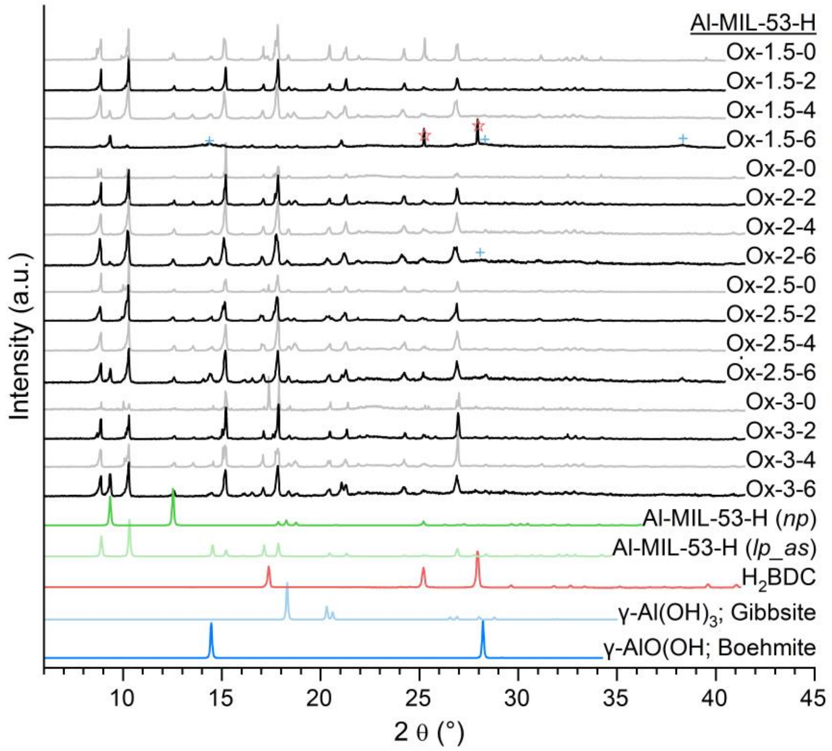

Figure S2.5. Measured PXRD patterns of the samples obtained in the HT-screening using terephthalic acid as the linker, oxalic acid (HOx) as the coordination modulator and NaOH as the pH modulator. For comparison, a measured PXRD pattern of the linker (H_2BDC) and calculated PXRD patterns of different polymorphs of $AlO_x(OH)_y$ (Boehmite (ICSD: 27865) and Gibbsite (ICSD: 36233))[3] are shown. In Addition, the PXRD data of Al-MIL-53-H_*lp* and Al-MIL-53-H_*np* is shown.[4] ☆ highlight reflections corresponding to recrystallized linker and + mark reflections corresponding to different polymorphs of $AlO_x(OH)_y$.

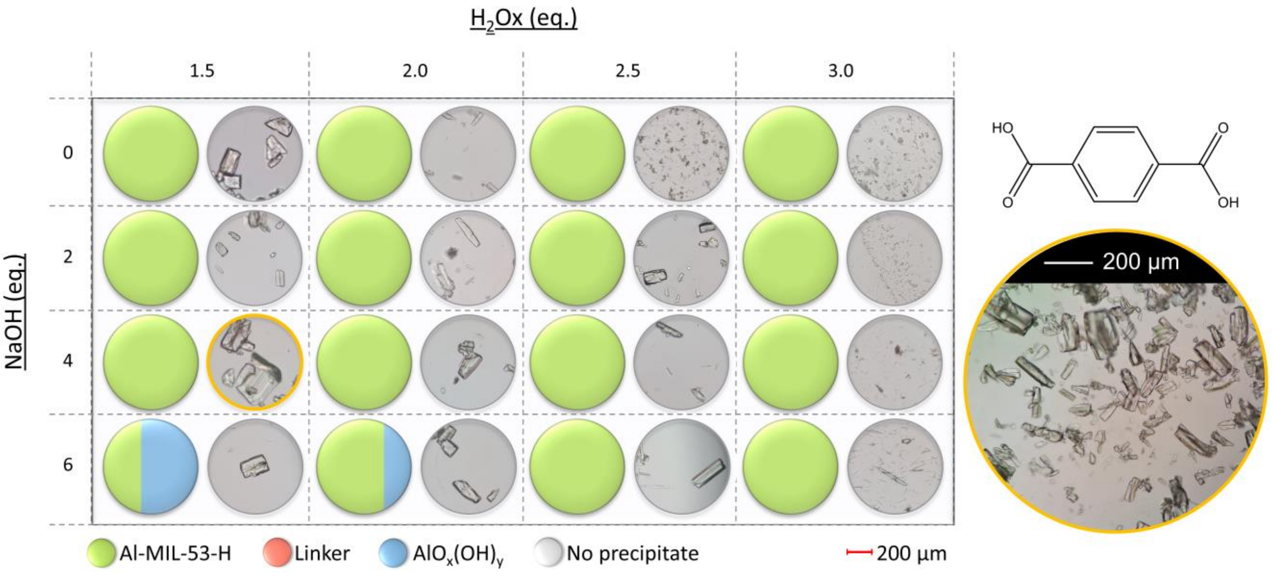

Figure S2.6. Crystallization diagram of the crystalline phases observed in the chemical system $AlCl_3$ / H_2BDC / oxalic acid / NaOH in H_2O for reactions carried out at 200 °C with different molar ratios of oxalic acid and NaOH, keeping the amount of $AlCl_3$ and H_2BDC constant. The crystalline phases observed are color-coded and for each reaction that led to Al-MIL-53-H, an optical micrograph of the crystals is shown. In addition, for the optimized reaction condition (yellow circle), an optical micrograph with a higher resolution and a larger number of crystals is shown.

2.1.3. Al-MIL-53-OH (Linker: H_2BDC-OH)

Table S2.5. List of starting materials employed in the systematic HT study of the chemical system $AlCl_3$ / **H_2BDC-OH** / oxalic acid (H_2Ox) / NaOH in water by varying the molar ratios of oxalic acid and NaOH. The reaction temperature was set to 170 °C and a heating program of 6-30-6 h (t_1-t_2-t_3) was used. One equivalent corresponds to 0.144 mmol.

**Synthesis-No.**	**Molar ratios (eq.)**				***V***					**Reaction product(s)**
	**$AlCl_3$**	**H_2BDC-OH**	**H_2Ox**	**NaOH**	**$AlCl_3$[a]** (µL)	**H_2BDC-OH** (mg)	**H_2Ox[b]** (µL / mg)	**NaOH** (µL)	**H_2O** (µL)	
**Al-MIL-53-OH** (Ox-1.5-0.0)	1	1	1.5	0.0	100	26.2	300 µL	/	400	no precipitate
(Ox-2.0-0.0)			2.0				400 µL		300	no precipitate
(Ox-2.5-0.0)			2.5				500 µL		200	no precipitate
(Ox-3.0-0.0)			3.0				600 µL		100	no precipitate
(Ox-1.5-2.0)			1.5	2.0			300 µL	48[c]	352	Al-MIL-53-OH
(Ox-2.0-2.0)			2.0				400 µL		252	Al-MIL-53-OH
(Ox-2.5-2.0)			2.5				500 µL		152	Al-MIL-53-OH
(Ox-3.0-2.0)			3.0				600 µL		52	Al-MIL-53-OH
(Ox-1.5-4.0)			1.5	4.0			300 µL	96[c]	304	Al-MIL-53-OH
(Ox-2.0-4.0)			2.0				400 µL		204	Al-MIL-53-OH
(Ox-2.5-4.0)			2.5				500 µL		104	Al-MIL-53-OH
(Ox-3.0-4.0)			3.0				600 µL		4	Al-MIL-53-OH
(Ox-1.5-6.0)			1.5	6.0			300 µL	144[c]	256	Al-MIL-53-OH
(Ox-2.0-6.0)			2.0				400 µL		156	Al-MIL-53-OH
(Ox-2.5-6.0)			2.5				500 µL		56	no precipitate
(Ox-3.0-6.0)			3.0				54.5 mg		556	no precipitate

[a] c_{AlCl3} = 1.44 mol/L; [b] c_{H2Ox} = 0.72 mol/L; [c] c_{NaOH} = 6.0 mol/L

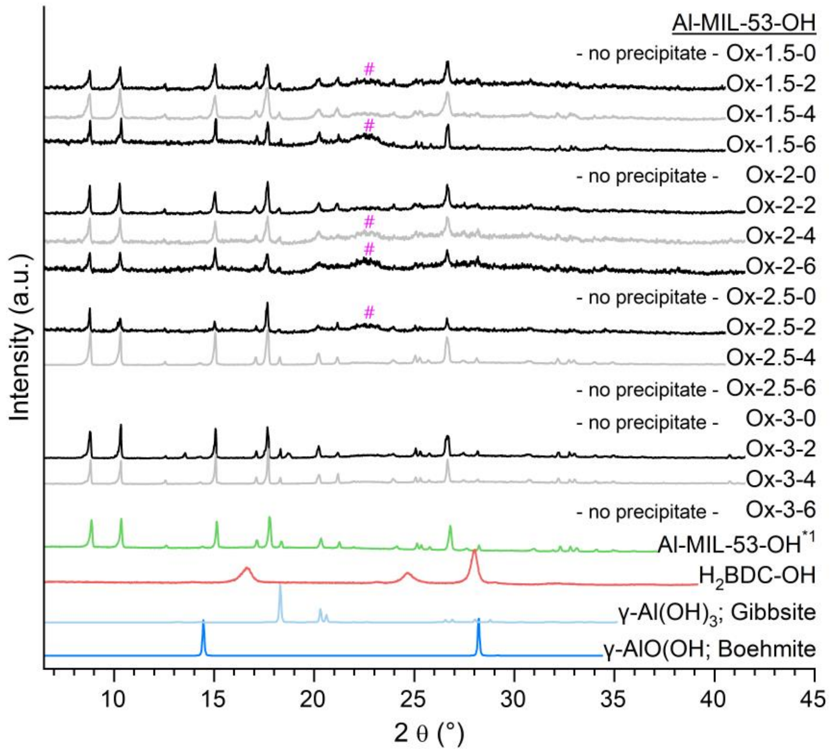

Figure S2.6. Measured PXRD patterns of the samples obtained in the HT-screening using hydroxyterephthalic acid as the linker, oxalic acid (HOx) as the coordination modulator and NaOH as the pH modulator. For comparison, a measured PXRD pattern of the linker (H_2BDC-OH) and calculated PXRD patterns of different polymorphs of $AlO_x(OH)_y$ (Boehmite (ICSD: 27865) and Gibbsite (ICSD: 36233))[3] are shown. *1In Addition, the PXRD data of Al-MIL-53-OH used to confirm the phase purity (Section S3) is shown, which was ground prior to measurement to avoid additional scattering effects due to the presence of large crystals. ☆The broad band at 21 – 24 °2θ is related to the presence of filtration paper used to prepare the samples.

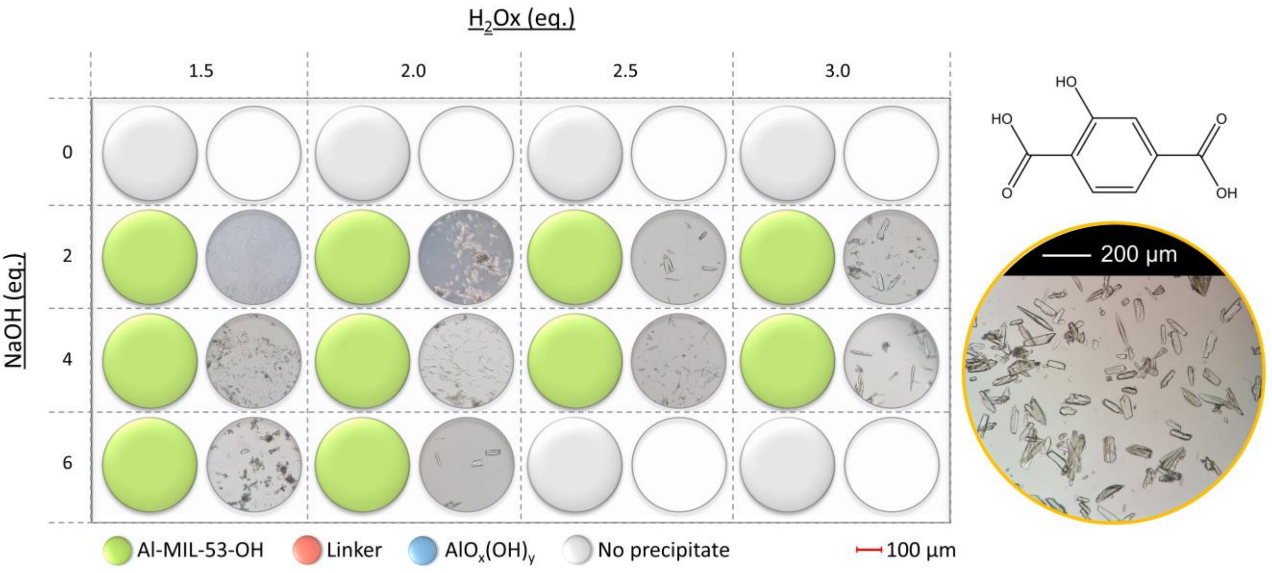

Figure S2.8. Crystallization diagram of the crystalline phases observed in the chemical system $AlCl_3$ / H_2BDC-OH / oxalic acid / NaOH in H_2O for reactions carried out at 170 °C with different molar ratios of oxalic acid and NaOH, keeping the amount of $AlCl_3$ and H_2BDC-OH constant. The crystalline phases observed are color-coded and the light grey circles mark the reactions where no precipitate was obtained. For each reaction that led to Al-MIL-53-OH, an optical micrograph of the crystals is shown. In addition, for the optimized reaction condition (yellow circle), an optical micrograph with a higher resolution and a larger number of crystals is shown.

2.1.4. Al-MIL-53-Br (Linker: H_2BDC-Br)

Table S2.6. List of starting materials employed in the systematic HT study of the chemical system $AlCl_3$ / **H_2BDC-Br** / oxalic acid (H_2Ox) / NaOH in water by varying the molar ratios of oxalic acid and NaOH. The reaction temperature was set to 210 °C and a heating program of 6-30-6 h (t_1-t_2-t_3) was used. One equivalent corresponds to 0.144 mmol.

Synthesis-No.	Molar ratios (eq.)				$V_{solution}$ (µL) / m (mg)					Reaction product(s)
	$AlCl_3$	H_2BDC-Br	H_2Ox	NaOH	$AlCl_3$[a] (µL)	H_2BDC-Br (mg)	H_2Ox[b] (µL / mg)	NaOH (µL)	H_2O (µL)	
**Al-MIL-53-Br** (Ox-1.5-0.0)	1	1	1.5	0.0	100	35.3	300 µL	/	400	Al-MIL-53-Br
(Ox-2.0-0.0)	1	1	2.0	0.0	100	35.3	400 µL	/	300	Al-MIL-53-Br
(Ox-2.5-0.0)	1	1	2.5	0.0	100	35.3	500 µL	/	200	Al-MIL-53-Br
(Ox-3.0-0.0)	1	1	3.0	0.0	100	35.3	600 µL	/	100	Al-MIL-53-Br
(Ox-1.5-2.0)	1	1	1.5	2.0	100	35.3	300 µL	48[c]	352	Al-MIL-53-Br
(Ox-2.0-2.0)	1	1	2.0	2.0	100	35.3	400 µL	48[c]	252	Al-MIL-53-Br
(Ox-2.5-2.0)	1	1	2.5	2.0	100	35.3	500 µL	48[c]	152	Al-MIL-53-Br
(Ox-3.0-2.0)	1	1	3.0	2.0	100	35.3	600 µL	48[c]	52	Al-MIL-53-Br
(Ox-1.5-4.0)	1	1	1.5	4.0	100	35.3	300 µL	96[c]	304	Al-MIL-53-Br
(Ox-2.0-4.0)	1	1	2.0	4.0	100	35.3	400 µL	96[c]	204	Al-MIL-53-Br
(Ox-2.5-4.0)	1	1	2.5	4.0	100	35.3	500 µL	96[c]	104	Al-MIL-53-Br
(Ox-3.0-4.0)	1	1	3.0	4.0	100	35.3	600 µL	96[c]	4	Al-MIL-53-Br
(Ox-1.5-6.0)	1	1	1.5	6.0	100	35.3	300 µL	144[c]	256	$AlO_x(OH)_y$
(Ox-2.0-6.0)	1	1	2.0	6.0	100	35.3	400 µL	144[c]	156	$AlO_x(OH)_y$
(Ox-2.5-6.0)	1	1	2.5	6.0	100	35.3	500 µL	144[c]	56	Al-MIL-53-Br, $AlO_x(OH)_y$
(Ox-3.0-6.0)	1	1	3.0	6.0	100	35.3	54.5 mg	144[c]	556	Al-MIL-53-Br, $AlO_x(OH)_y$

[a] c_{AlCl3} = 1.44 mol/L; [b] c_{H2Ox} = 0.72 mol/L; [c] c_{NaOH} = 6.0 mol/L

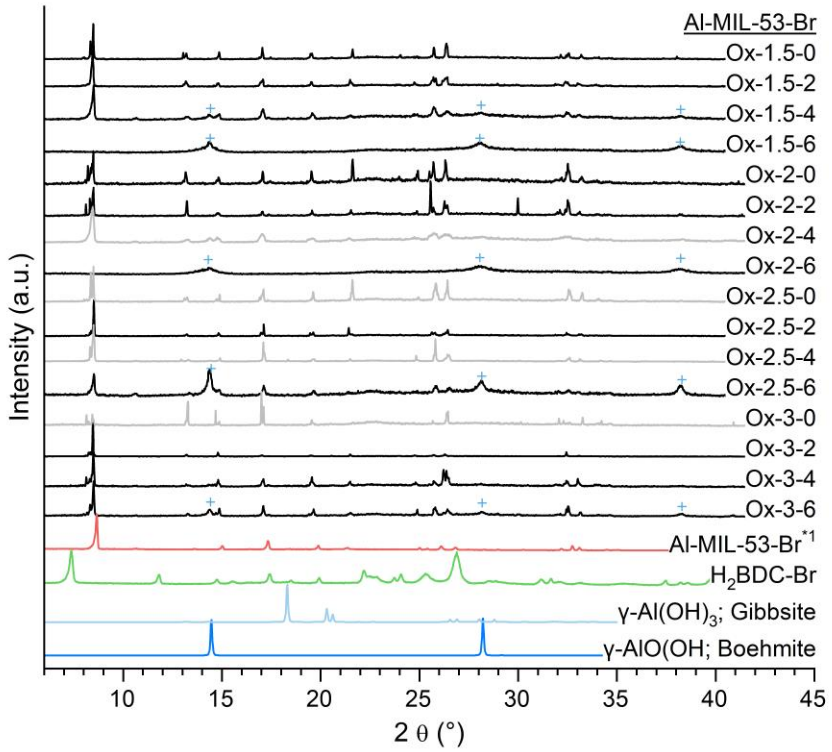

Figure S2.9. Measured PXRD patterns of the samples obtained in the HT-screening using bromoterephthalic acid as the linker, oxalic acid (HOx) as the coordination modulator and NaOH as the pH modulator. For comparison, a measured PXRD pattern of the linker (H_2BDC-Br) and calculated PXRD patterns of different polymorphs of $AlO_x(OH)_y$ (Boehmite (ICSD: 27865) and Gibbsite (ICSD: 36233))[3] are shown. *1In Addition, the PXRD data of Al-MIL-53-Br used to confirm the phase purity (Section S3) is shown, which was ground prior to measurement to avoid additional scattering effects due to the presence of large crystals. + highlight reflections corresponding to different polymorphs of $AlO_x(OH)_y$.

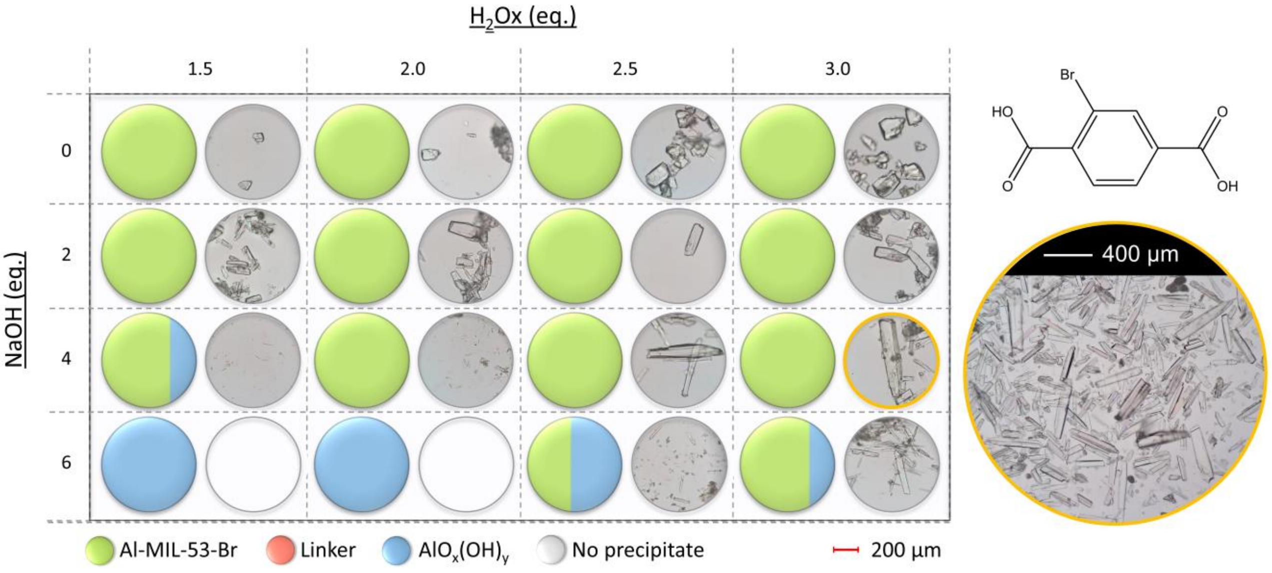

Figure S2.10. Crystallization diagram of the crystalline phases observed in the chemical system $AlCl_3$ / H_2BDC-Br / oxalic acid / NaOH in H_2O for reactions carried out at 210 °C with different molar ratios of oxalic acid and NaOH, keeping the amount of $AlCl_3$ and H_2BDC-Br constant. The crystalline phases observed are color-coded and for each reaction that led to Al-MIL-53-Br, an optical micrograph of the crystals is shown. In addition, for the optimized reaction condition (yellow circle), an optical micrograph with a higher resolution and a larger number of crystals is shown.

2.1.5. Al-MIL-53-COOH (Linker: H_2BDC-COOH)

Table S2.7. List of starting materials employed in the systematic HT study of the chemical system $AlCl_3$ / **H_2BDC-COOH** / oxalic acid (H_2Ox) / NaOH in water by varying the molar ratios of oxalic acid and NaOH. The reaction temperature was set to 170 °C and a heating program of 6-30-6 h (t_1-t_2-t_3) was used. One equivalent corresponds to 0.144 mmol.

**Synthesis-No.**	**Molar ratios (eq.)**				**$V_{solution}$ (µL) / *m* (mg)**					**Reaction product(s)**
	**$AlCl_3$**	**H_2BDC-COOH**	**H_2Ox**	**NaOH**	**$AlCl_3$[a]** (µL)	**H_2BDC-COOH** (mg)	**H_2Ox[b]** (µL / mg)	**NaOH** (µL)	**H_2O** (µL)	
**Al-MIL-53-COOH** (Ox-1.5-0.0)	1	1	1.5	0.0	100	27.7	300 µL	/	400	no precipitate
(Ox-2.0-0.0)			2.0				400 µL		300	no precipitate
(Ox-2.5-0.0)			2.5				500 µL		200	no precipitate
(Ox-3.0-0.0)			3.0				600 µL		100	no precipitate
(Ox-1.5-2.0)			1.5	2.0			300 µL	48[c]	352	Al-MIL-53-COOH
(Ox-2.0-2.0)			2.0				400 µL		252	Al-MIL-53-COOH
(Ox-2.5-2.0)			2.5				500 µL		152	Al-MIL-53-COOH
(Ox-3.0-2.0)			3.0				600 µL		52	Al-MIL-53-COOH
(Ox-1.5-4.0)			1.5	4.0			300 µL	96[c]	304	Al-MIL-53-COOH
(Ox-2.0-4.0)			2.0				400 µL		204	Al-MIL-53-COOH
(Ox-2.5-4.0)			2.5				500 µL		104	Al-MIL-53-COOH
(Ox-3.0-4.0)			3.0				600 µL		4	Al-MIL-53-COOH
(Ox-1.5-6.0)			1.5	6.0			300 µL	144[c]	256	Al-MIL-53-COOH
(Ox-2.0-6.0)			2.0				400 µL		156	Al-MIL-53-COOH
(Ox-2.5-6.0)			2.5				500 µL		56	Al-MIL-53-COOH
(Ox-3.0-6.0)			3.0				54.5 mg		556	no precipitate

[a] c_{AlCl3} = 1.44 mol/L; [b] c_{H2Ox} = 0.72 mol/L; [c] c_{NaOH} = 6.0 mol/L

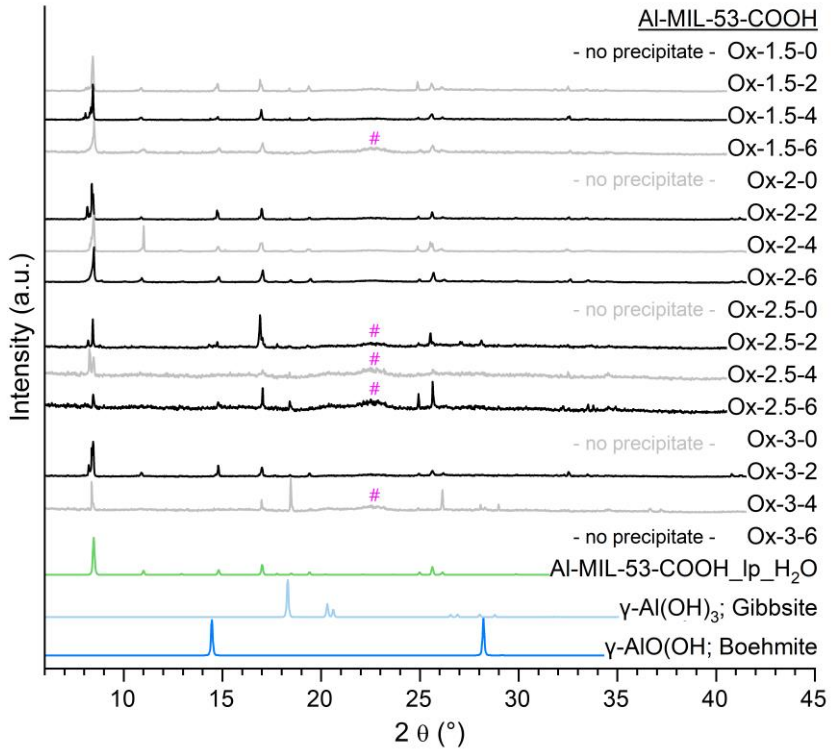

Figure S2.11. Measured PXRD patterns of the samples obtained in the HT-screening using trimellitic acid as the linker, oxalic acid (HOx) as the coordination modulator and NaOH as the pH modulator. To increase reproducibility and comparability, all samples were soaked with water prior to measurement to obtain the water-rich, *large-pore* form of Al-MIL-53-COOH_*lp*_H_2O. For comparison, the measured PXRD pattern of the linker (H_2BDC-COOH) and a calculated PXRD pattern of different polymorphs of $AlO_x(OH)_y$ (Boehmite (ICSD: 27865) and Gibbsite (ICSD: 36233))[3] and Al-MIL-53-COOH_*lp*_H_2O obtained from Rietveld refinement against PXRD data, are shown. *The broad band at 21 – 24 °2θ is related to the presence of filtration paper used to prepare the samples.

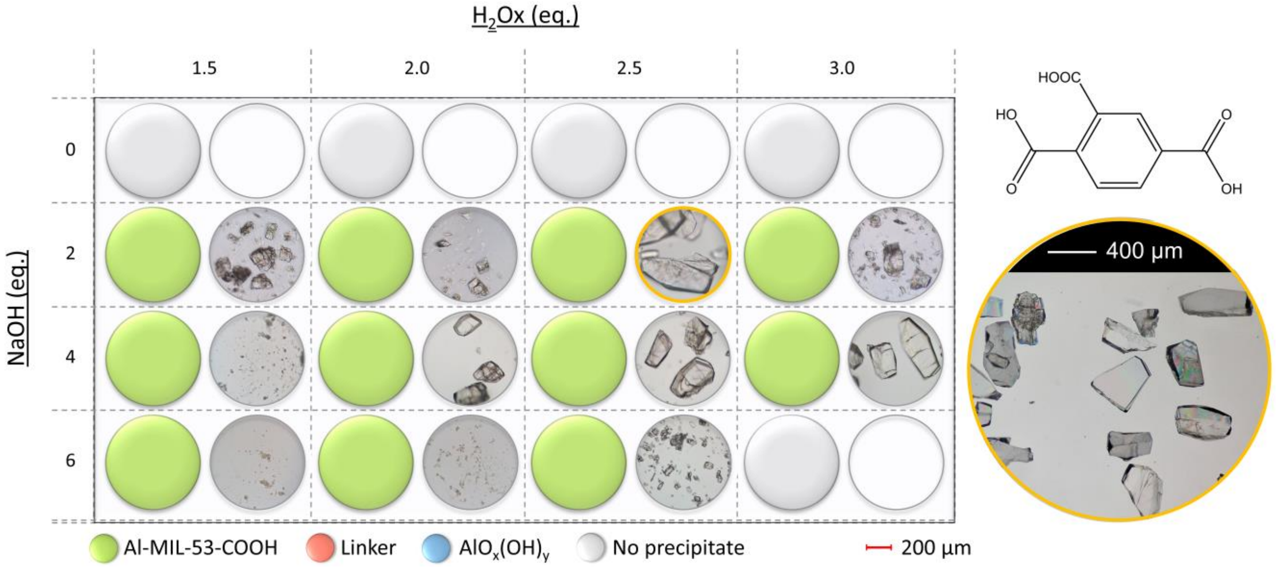

Figure S2.12. Crystallization diagram of the crystalline phases observed in the chemical system $AlCl_3$ / H_2BDC-COOH / oxalic acid / NaOH in H_2O for reactions carried out at 170 °C with different molar ratios of oxalic acid and NaOH, keeping the amount of $AlCl_3$ and H_2BDC-COOH constant. The crystalline phases observed are color-coded and the light grey circles mark the reactions where no precipitate was obtained. For each reaction that led to Al-MIL-53-COOH, an optical micrograph of the crystals is shown. In addition, for the optimized reaction condition (yellow circle), an optical micrograph with a higher resolution and a larger number of crystals is shown.

2.1.6. Al-MIL-121, Al-MIL-118A, Al-MIL-120 (Linker: H_4BTEC)

Table S2.8. List of starting materials employed in the systematic HT study of the chemical system $AlCl_3$ / **H_4BTEC** / oxalic acid (H_2Ox) / NaOH in water by varying the molar ratios of oxalic acid and NaOH. The reaction temperature was set to 200 °C and a heating program of 6-30-6 h (t_1-t_2-t_3) was used. One equivalent corresponds to 0.512 mmol.

**Synthesis-No.**	**Molar ratios (eq.)**				**$V_{solution}$ (µL) / *m* (mg)**					**Reaction product(s)**
	**$AlCl_3$**	**H_4BTEC**	**H_2Ox**	**NaOH**	**$AlCl_3$[a] (µL)**	**H_4BTEC (mg)**	**H_2Ox[b] (µL / mg)**	**NaOH (µL)**	**H_2O (µL)**	
**Al-MIL-121** (Ox-1.5-0.0)	1	0.5	1.5	0.0	357	65.0	96.8 mg	/	443	Al-MIL-121
**Al-MIL-121** (Ox-2.0-0.0)			2.0				129.1 mg		443	Al-MIL-121
**Al-MIL-121** (Ox-2.5-0.0)			2.5				161.4 mg		443	Al-MIL-121
**Al-MIL-121** (Ox-3.0-0.0)			3.0				193.6 mg		443	Al-MIL-121
**Al-MIL-118A** (Ox-1.5-2.0)			1.5	2.0			96.8 mg	171[c]	273	Al-MIL-118A
**Al-MIL-118A** (Ox-2.0-2.0)			2.0				129.1 mg		273	Al-MIL-118A
**Al-MIL-118A** (Ox-2.5-2.0)			2.5				161.4 mg		273	Al-MIL-118A
**Al-MIL-118A** (Ox-3.0-2.0)			3.0				193.6 mg		273	Al-MIL-118A
**Al-MIL-118A** (Ox-1.5-4.0)			1.5	4.0			96.8 mg	341[c]	102	Al-MIL-118A, Al-MIL-120
**Al-MIL-118A** (Ox-2.0-4.0)			2.0				129.1 mg		102	Al-MIL-118A
**Al-MIL-118A** (Ox-2.5-4.0)			2.5				161.4 mg		102	Al-MIL-118A, Al-MIL-120
**Al-MIL-118A** (Ox-3.0-4.0)			3.0				193.6 mg		102	Al-MIL-118A, Al-MIL-120
**Al-MIL-120** (Ox-1.5-6.0)			1.5	6.0			96.8 mg	256[e]	187	Al-MIL-120, $AlO_x(OH)_y$
**Al-MIL-120** (Ox-2.0-6.0)			2.0				129.1 mg		187	Al-MIL-120
**Al-MIL-118A** (Ox-2.5-6.0)			2.5				161.4 mg		187	Al-MIL-118A, Al-MIL-120
**Al-MIL-118A** (Ox-3.0-6.0)			3.0				193.6 mg		187	Al-MIL-118A

[a] c_{AlCl3} = 1.44 mol/L; [b] c_{H2Ox} = 0.72 mol/L; [c] c_{NaOH} = 6.0 mol/L; [d] c_{NaOH} = 2.0 mol/L; [e] c_{NaOH} = 12.0 mol/L

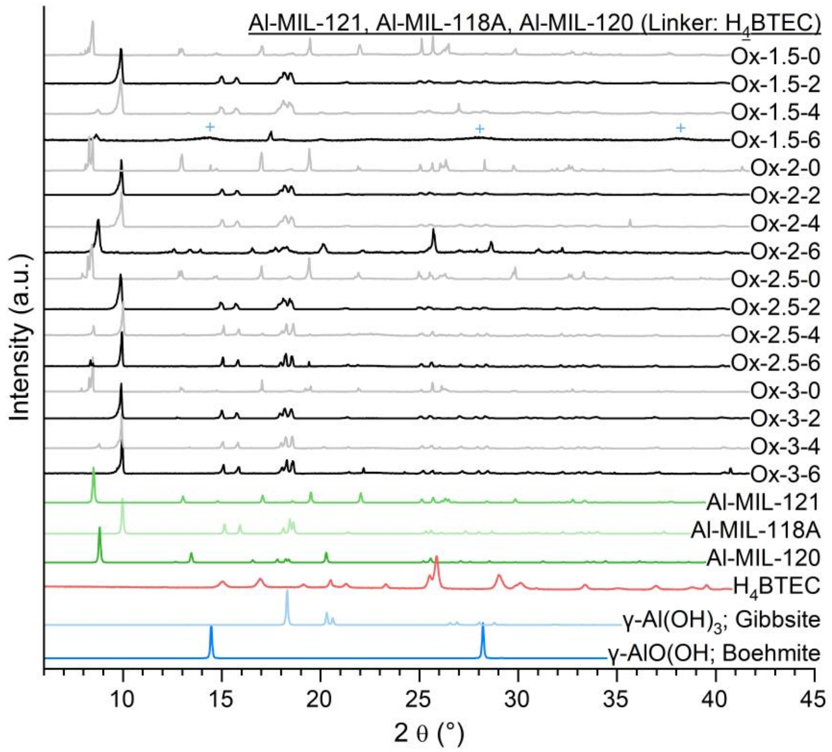

Figure S2.13. Measured PXRD patterns of the samples obtained in the HT-screening using pyromellitic acid as the linker, oxalic acid (HOx) as the coordination modulator and NaOH as the pH modulator. For comparison, a measured PXRD pattern of the linker (H_4BTEC) and a calculated PXRD pattern of Al-MIL-121, Al-MIL-118A, Al-MIL-120[5,6] and different polymorphs of $AlO_x(OH)_y$ (Boehmite (ICSD: 27865) and Gibbsite (ICSD: 36233))[3] are shown. + highlight reflections corresponding to different polymorphs of $AlO_x(OH)_y$.

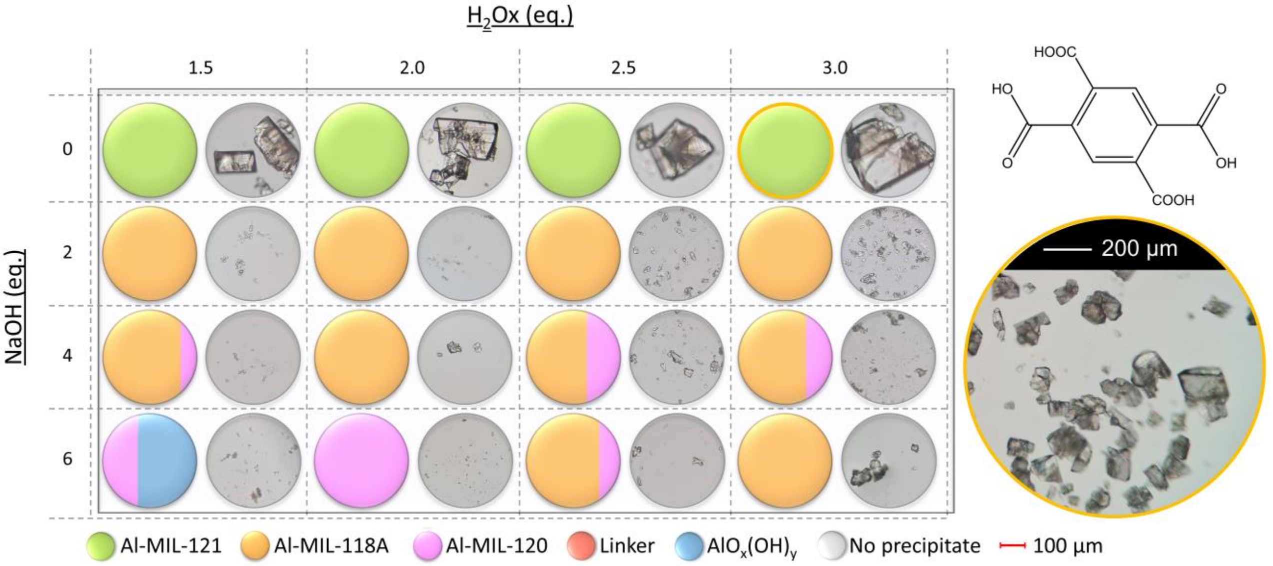

Figure S2.14. Crystallization diagram of the crystalline phases observed in the chemical system $AlCl_3$ / H_4BTEC / oxalic acid / NaOH in H_2O for reactions carried out at 200 °C with different molar ratios of oxalic acid and NaOH, keeping the amount of $AlCl_3$ and H_4BTEC constant. The crystalline phases observed are color-coded and for each reaction that led to Al-MIL-121, Al-MIL-118A or Al-MIL-120, an optical micrograph of the crystals is shown. In addition, for the optimized reaction condition for Al-MIL-121 (yellow circle), an optical micrograph with a higher resolution and a larger number of crystals is shown.

2.1.7. Al-MIL-96 (Linker: H_3BTC)

Table S2.9. List of starting materials employed in the systematic HT study of the chemical system $AlCl_3$ / **H_3BTC** / oxalic acid (H_2Ox) / NaOH in water by varying the molar ratios of oxalic acid and NaOH. The reaction temperature was set to 200 °C and a heating program of 6-30-6 h (t_1-t_2-t_3) was used. One equivalent corresponds to 0.144 mmol.

Synthesis-No.	Molar ratios (eq.)				$V_{solution}$ (µL) / *m* (mg)					Reaction product(s)
	$AlCl_3$	H_3BTC	H_2Ox	NaOH	$AlCl_3$[a] (µL)	H_3BTC(mg)	H_2Ox[b] (µL / mg)	NaOH (µL)	H_2O (µL)	
**Al-MIL-96** (Ox-1.5-0.0)	1	0.5	1.5	0.0	100	15.1	300 µL	/	400	Al-MIL-96
(Ox-2.0-0.0)			2.0				400 µL		300	Al-MIL-96
(Ox-2.5-0.0)			2.5				500 µL		200	Al-MIL-96
(Ox-3.0-0.0)			3.0				600 µL		100	Al-MIL-96
(Ox-1.5-2.0)			1.5	2.0			300 µL	48[c]	352	Al-MIL-96
(Ox-2.0-2.0)			2.0				400 µL		252	Al-MIL-96
(Ox-2.5-2.0)			2.5				500 µL		152	Al-MIL-96
(Ox-3.0-2.0)			3.0				600 µL		52	Al-MIL-96
(Ox-1.5-4.0)			1.5	4.0			300 µL	96[c]	304	Al-MIL-96
(Ox-2.0-4.0)			2.0				400 µL		204	Al-MIL-96
(Ox-2.5-4.0)			2.5				500 µL		104	Al-MIL-96
(Ox-3.0-4.0)			3.0				600 µL		4	Al-MIL-96
(Ox-1.5-6.0)			1.5	6.0			300 µL	144[c]	256	$AlO_x(OH)_y$
(Ox-2.0-6.0)			2.0				400 µL		156	$AlO_x(OH)_y$
(Ox-2.5-6.0)			2.5				500 µL		56	Al-MIL-96
(Ox-3.0-6.0)			3.0				54.5 mg		556	Al-MIL-96

[a] c_{AlCl3} = 1.44 mol/L; [b] c_{H2Ox} = 0.72 mol/L; [c] c_{NaOH} = 6.0 mol/L

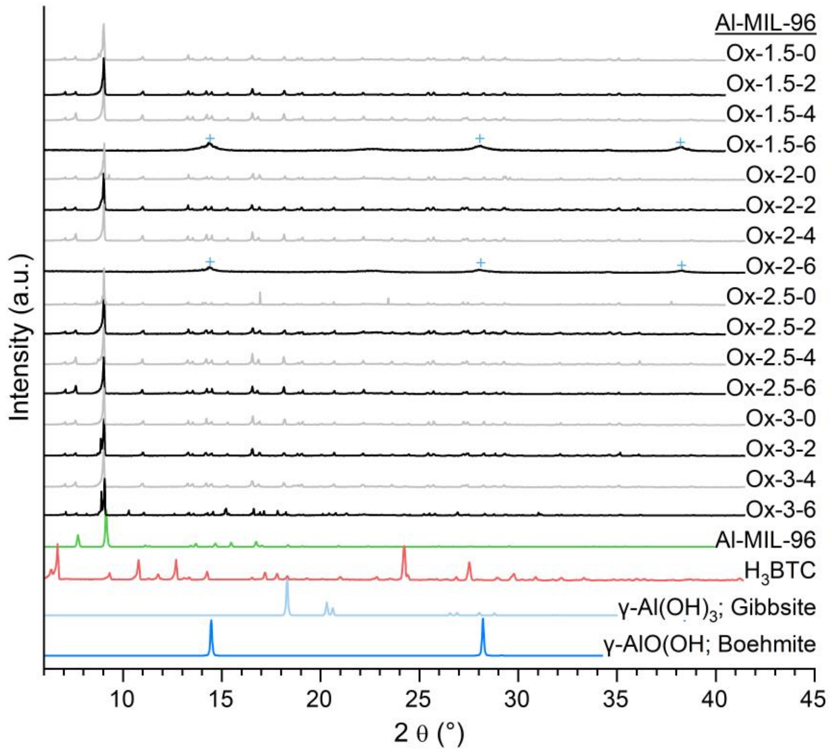

Figure S2.15. Measured PXRD patterns of the samples obtained in the HT-screening using trimesic acid as the linker, oxalic acid (HAc) as the coordination modulator and NaOH as the pH modulator. For comparison, a measured PXRD pattern of the linker (H_3BTC) and a calculated PXRD pattern of Al-MIL-96[7] and different polymorphs of $AlO_x(OH)_y$ (Boehmite (ICSD: 27865) and Gibbsite (ICSD: 36233))[3] are shown. + highlight reflections corresponding to different polymorphs of $AlO_x(OH)_y$.

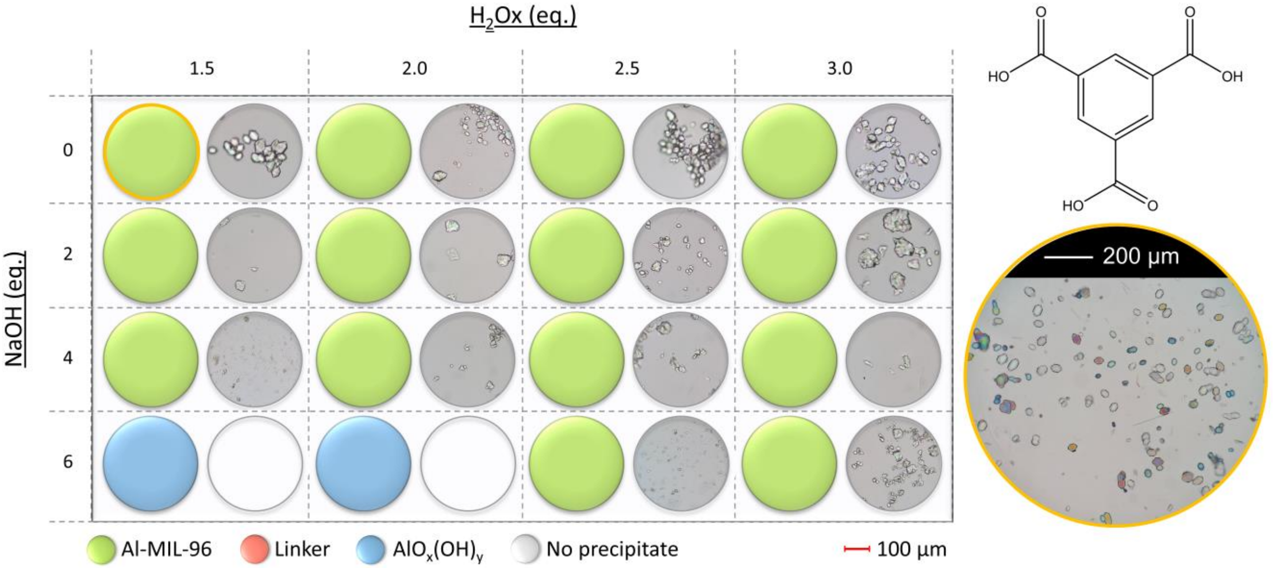

Figure S2.16. Crystallization diagram of the crystalline phases observed in the chemical system $AlCl_3$ / H_3BTC / oxalic acid / NaOH in H_2O for reactions carried out at 200 °C with different molar ratios of oxalic acid and NaOH, keeping the amount of $AlCl_3$ and H_3BTC constant. The crystalline phases observed are color-coded and for each reaction that led to Al-MIL-96, an optical micrograph of the crystals is shown. In addition, for the optimized reaction condition (yellow circle), an optical micrograph with a higher resolution and a larger number of crystals is shown.

2.1.8. Al(OH)(1,4-NDC) (Linker: 1,4-H_2NDC)

Table S2.10. List of starting materials employed in the systematic HT study of the chemical system $AlCl_3$ / **1,4-H_2NDC** / oxalic acid (H_2Ox) / NaOH in water by varying the molar ratios of oxalic acid and NaOH. The reaction temperature was set to 170 °C and a heating program of 6-30-6 h (t_1-t_2-t_3) was used. One equivalent corresponds to 0.072 mmol.

<table>
<tr><th rowspan="2">Synthesis-No.</th><th colspan="4">Molar ratios (eq.)</th><th colspan="5">V$V_{solution}$ (µL) / m (mg)</th><th rowspan="2">Reaction product(s)</th></tr>
<tr><th>$AlCl_3$</th><th>1,4-H_2NDC</th><th>H_2Ox</th><th>NaOH</th><th>$AlCl_3$[a] (µL)</th><th>1,4-H_2NDC (mg)</th><th>H_2Ox[b] (µL / mg)</th><th>NaOH (µL)</th><th>H_2O (µL)</th></tr>
<tr><td>Al(OH)(1,4-NDC)
(Ox-1.5-0.0)</td><td rowspan="16">1</td><td rowspan="16">0.5</td><td>1.5</td><td rowspan="4">0.0</td><td rowspan="16">50</td><td rowspan="16">7.8</td><td>150 µL</td><td rowspan="4">/</td><td>600</td><td>Al(OH)(1,4-NDC)</td></tr>
<tr><td>(Ox-2.0-0.0)</td><td>2.0</td><td>200 µL</td><td>550</td><td>Al(OH)(1,4-NDC),
1,4-H_2NDC</td></tr>
<tr><td>(Ox-2.5-0.0)</td><td>2.5</td><td>250 µL</td><td>500</td><td>Al(OH)(1,4-NDC),
1,4-H_2NDC</td></tr>
<tr><td>(Ox-3.0-0.0)</td><td>3.0</td><td>300 µL</td><td>450</td><td>Al(OH)(1,4-NDC),
1,4-H_2NDC</td></tr>
<tr><td>(Ox-1.5-2.0)</td><td>1.5</td><td rowspan="4">2.0</td><td>150 µL</td><td rowspan="4">72[d]</td><td>528</td><td>Al(OH)(1,4-NDC)</td></tr>
<tr><td>(Ox-2.0-2.0)</td><td>2.0</td><td>200 µL</td><td>478</td><td>Al(OH)(1,4-NDC)</td></tr>
<tr><td>(Ox-2.5-2.0)</td><td>2.5</td><td>250 µL</td><td>428</td><td>Al(OH)(1,4-NDC)</td></tr>
<tr><td>(Ox-3.0-2.0)</td><td>3.0</td><td>300 µL</td><td>378</td><td>Al(OH)(1,4-NDC)</td></tr>
<tr><td>(Ox-1.5-4.0)</td><td>1.5</td><td rowspan="4">4.0</td><td>150 µL</td><td rowspan="4">144[d]</td><td>456</td><td>Al(OH)(1,4-NDC)</td></tr>
<tr><td>(Ox-2.0-4.0)</td><td>2.0</td><td>200 µL</td><td>406</td><td>Al(OH)(1,4-NDC)</td></tr>
<tr><td>(Ox-2.5-4.0)</td><td>2.5</td><td>250 µL</td><td>356</td><td>Al(OH)(1,4-NDC)</td></tr>
<tr><td>(Ox-3.0-4.0)</td><td>3.0</td><td>300 µL</td><td>306</td><td>Al(OH)(1,4-NDC)</td></tr>
<tr><td>(Ox-1.5-6.0)</td><td>1.5</td><td rowspan="4">6.0</td><td>150 µL</td><td rowspan="4">216[d]</td><td>384</td><td>$AlO_x(OH)_y$</td></tr>
<tr><td>(Ox-2.0-6.0)</td><td>2.0</td><td>200 µL</td><td>334</td><td>Al(OH)(1,4-NDC),
$AlO_x(OH)_y$</td></tr>
<tr><td>(Ox-2.5-6.0)</td><td>2.5</td><td>250 µL</td><td>284</td><td>Al(OH)(1,4-NDC)</td></tr>
<tr><td>(Ox-3.0-6.0)</td><td>3.0</td><td>300 µL</td><td>234</td><td>Al(OH)(1,4-NDC),
1,4-H_2NDC</td></tr>
</table>

[a] c_{AlCl3} = 1.44 mol/L; [b] c_{H2Ox} = 0.72 mol/L; [c] c_{NaOH} = 6.0 mol/L, [d] c_{NaOH} = 2.0 mol/L

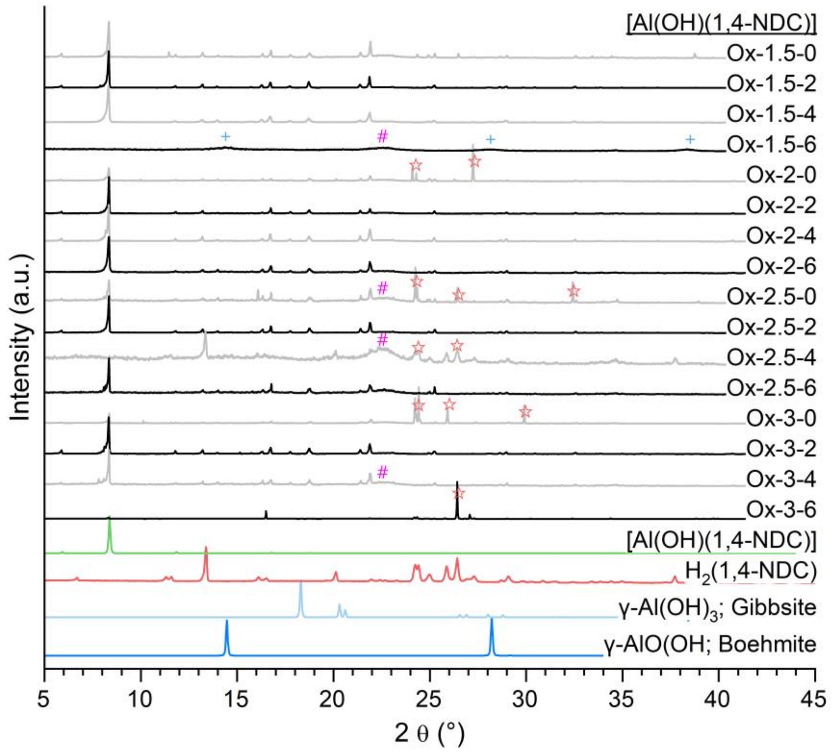

Figure S2.17. Measured PXRD patterns of the samples obtained in the HT-screening using 1,4-naphthalenedicarboxylic acid as the linker, oxalic acid (HOx) as the coordination modulator and NaOH as the pH modulator. For comparison, a measured PXRD pattern of the linker (H_2(1,4-NDC)) and a calculated PXRD pattern of [Al(OH)(1,4-NDC)][8] and different polymorphs of $AlO_x(OH)_y$ (Boehmite (ICSD: 27865) and Gibbsite (ICSD: 36233))[3] are shown.☆The broad band at 21 – 24 °2θ is related to the presence of filtration paper used to prepare the samples, ☆highlight reflections corresponding to recrystallized linker and + mark reflections corresponding to different polymorphs of $AlO_x(OH)_y$.

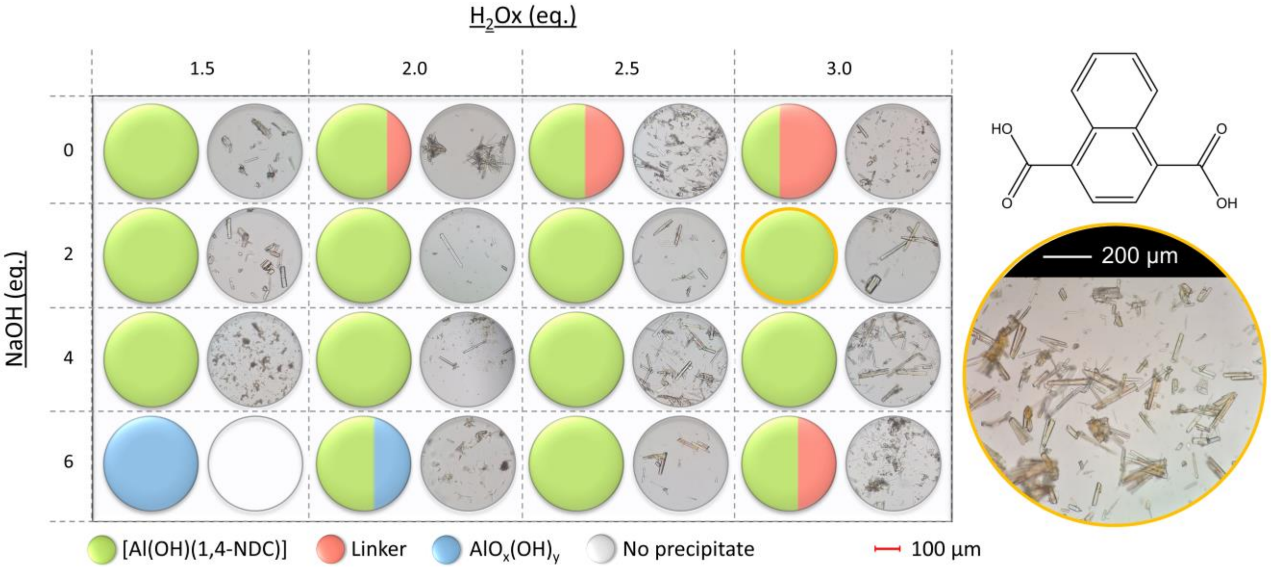

Figure S2.18. Crystallization diagram of the crystalline phases observed in the chemical system $AlCl_3$ / 1,4-H_2NDC / oxalic acid / NaOH in H_2O for reactions carried out at 170 °C with different molar ratios of oxalic acid and NaOH, keeping the amount of $AlCl_3$ and 1,4-H_2NDC constant. The crystalline phases observed are color-coded and for each reaction that led to Al(OH)(1,4-NDC), an optical micrograph of the crystals is shown. In addition, for the optimized reaction condition (yellow circle), an optical micrograph with a higher resolution and a larger number of crystals is shown.

3. Crystal Structure Determination and Refinement

All samples obtained using the optimized synthesis conditions (Section S2) were characterized by PXRD. Prior to measurements, all samples were homogenized by grinding and soaked in water to increase the reproducibility of the measurements. Single crystal X-ray diffraction (SCXRD) measurements were carried out on Al-MIL-53-NO_2 and Al-MIL-53-Br crystals that were suitable for this method. However, both compounds exhibited significant disorder. The phenyl ring was found disordered in three different orientations positions with an occupation factor of 1/3, resulting in the N-atom of the NO_2-group being highly disordered with an occupation factor of 1/12. Due to the high degree of disorder and the associated low occupation factors, it was not possible to locate the NO_2- and the Br-groups of the linker molecules in Al-MIL-53-NO_2 and Al-MIL-53-Br sufficiently. For Al-MIL-53-COOH, a lower degree of disorder of the phenyl ring and the free carboxylic acids was found, presumably due to the formation of hydrogen bonds between the free carboxylic acid groups of the linker and the μ-OH groups of the inorganic building units (see Figure S3.2d) exhibiting characteristic hydrogen donor–acceptor distances of 3.175 Å. This allowed us to elucidate the crystal structure using SCXRD. The crystal structure of the water-rich *large pore* form of Al-MIL-53-COOH containing water molecules (*lp*-H_2O) was elucidated by single crystal X-ray diffraction (SCXRD) and phase purity of the bulk material was confirmed by a Rietveld refinement (Section S3.1). For all other compounds obtained using the optimized synthesis conditions using oxalic acid as the modulator (Section S2), Le Bail fits against PXRD data (Figure S3.3 – S3.10) were carried out to confirm the phase purity and to determine the lattice parameters (Table S3.3 – S3.10).

3.1. Single-Crystal X-ray Diffraction and Rietveld Refinement of Al-MIL-53-COOH

The crystal structure of the water-rich *large pore* form of Al-MIL-53-COOH containing water molecules (*lp*-H_2O) was elucidated by single crystal X-ray diffraction (SCXRD) and phase purity of the bulk material was confirmed by a Rietveld refinement. The structural data (Table S3.1) for Al-MIL-53-COOH_*lp*-H_2O obtained from Rietveld refinement against powder X-ray diffraction data (Figure S3.1) and from single crystal X-ray diffraction data (Table S3.2) have been deposited with the Cambridge Crystallographic Data Center (CCDC-number 2432421 – 2432422).

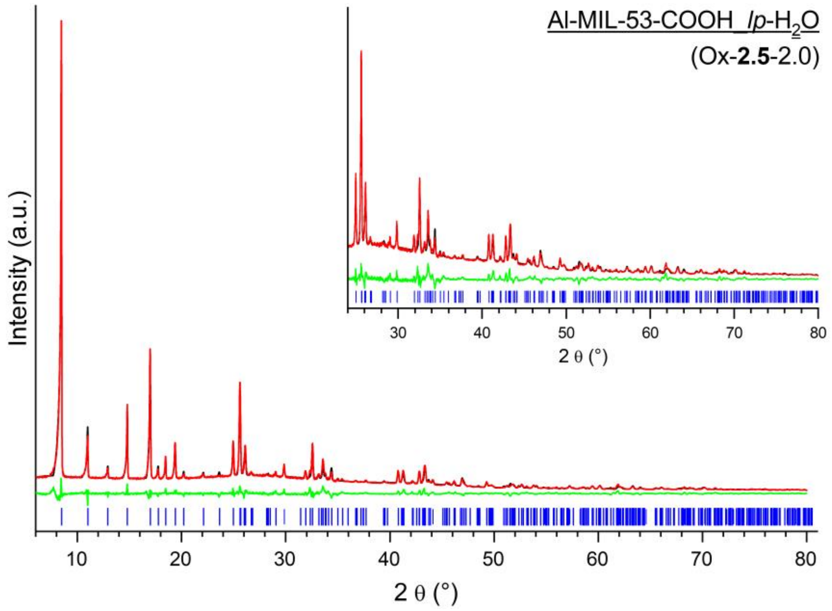

Figure S3.1. Final Rietveld plot of the structure refinement of Al-MIL-53-COOH_*lp*-H_2O (Ox-2.5-2.0). The experimental data (red line), the calculated diffractogram (black line), the resulting difference (green line) and the allowed Bragg reflection positions (blue ticks) are shown.

Table S3.1. Crystallographic data for Al-MIL-53-COOH_*lp*-H_2O (Ox-2.5-2.0) obtained from the Rietveld refinement (Figure S3.1). CCDC-2432422 contains the supplementary crystallographic data of Al-MIL-53-COOH_*lp*-H_2O (Ox-2.5-2.0). The crystallographic data of Al-MIL-53-COOH_*lp*-H_2O reported in the literature[9] are given for comparison.

	**Ox-2.5-2.0**	**Literature[9]**
**Sum formula**	$C_{18}Al_2O_{23.20}$	/
**Formula weight** (g/mol)	641.40	/
**CCDC number**	2432422	/
**Wavelength** (Å)	1.5406 Å	1.5406 Å
**Crystal system**	Orthorhombic	Orthorhombic
**Space group**	*Imma*	*Imcm*
a (Å)	16.0748(6)	16.006(6)
b (Å)	6.6459(2)	13.734(4)
c (Å)	13.6834(6)	6.641(1)
α (°)	90	90
β (°)	90	90
γ (°)	90	90
**Volume** (Å^3)	1461.82(10)	1460.0(6)
**Z**	2	/
R_{wp} (%)	5.91	/
**GoF** (%)	1.01	/

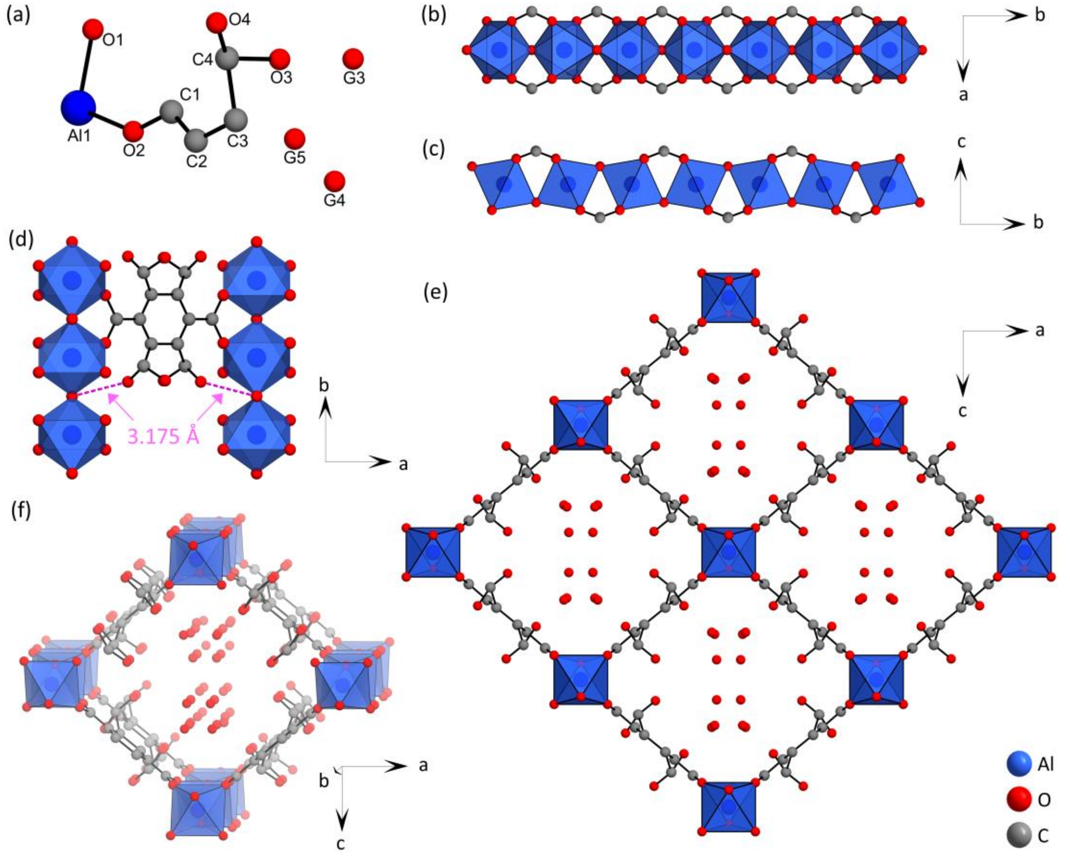

Figure S3.2. Crystal structure of Al-MIL-53-COOH_*lp*-H_2O (Ox-2.5-2.0) obtained from structure refinement against PXRD data. (**a**) Asymmetric unit. (**b**) and (**c**) Chains of corner-sharing [AlO_6]-polyhedra along the crystallographic *b*-axis. (**d**) Hydrogen bonds between the free carboxylic acid groups of the linker and the *μ*-OH groups of the inorganic building unit. The hydrogen donor-acceptor distance (3.175 Å) was calculated using structural data, determined from Rietveld-refinement against X-ray diffraction data. (**e**) and (**f**) Crystal structure of Al-MIL-53-COOH_*lp*_H_2O as seen along the pore channel along crystallographic *b*-axis occupied by water molecules.

Table S3.2. Selected crystallographic data and details of the structure refinements for Al-MIL-53-COOH_*lp*-H_2O obtained from single crystal X-ray diffraction data. CCDC-2432421 contains the supplementary crystallographic data of Al-MIL-53-COOH_*lp*-H_2O (Ox-2.5-2.0) obtained from single crystal X-ray diffraction data.

	Al-MIL-53-COOH_*lp*-H_2O
**Empirical formula**	AlC_9O_7
**Formula weight** (g/mol)	247.07
**Temperature** (K)	160.0
**Wavelength** (Å)	0.71073
**Crystal system**	Orthorhombic
**Space group**	*Imma*
a (Å)	16.470(3)
b (Å)	6.6300(13)
c (Å)	13.150(3)
α (°)	90
β (°)	90
γ (°)	90
**Volume** (Å^3)	1435.9(5)
**Z**	4
ρ_{calc} (g/cm^3)	1.143
**F(000)**	492
**Crystal size** (mm^3)	0.2 x 0.15 x 0.1
**2Θ range for data collection** (°)	3.098 to 27.505
**Index ranges**	-21<=h<=20, -8<=k<=8, -17<=l<=15
**Reflections collected**	5557
**Independent reflections**	913 [R_{int} = 0.0403]
**Parameters**	67
**Goodness-of-fit on F^2**	1.136
**Final R indexes [I>=2σ (I)]**	R_1 = 0.0812, wR_2 = 0.2338
**Final R indexes [all data]**	R_1 = 0.0867, wR_2 = 0.2427
**Largest diff. peak/hole** (e Å^{-3})	0.608 / -0.419

3.2. Le Bail plots and crystallographic data

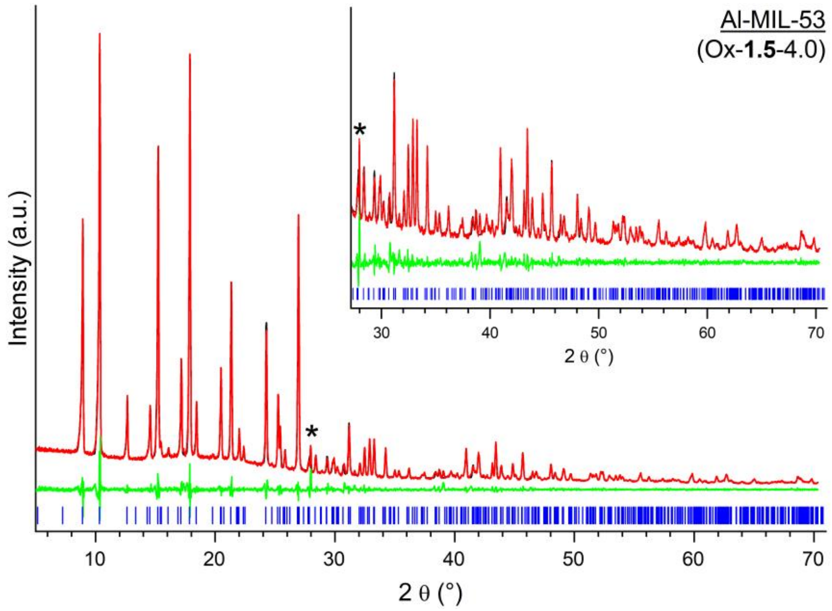

Figure S3.3. Final Le Bail-plot forAl-MIL-53_as (Ox-1.5-4.0) against PXRD data. The experimental data (red line), the calculated diffractogram (black line), the resulting difference (green line) and the allowed Bragg reflection positions (blue ticks) are shown. The '*' marks additional reflections that can be attributed to linker present as a crystalline side-product.

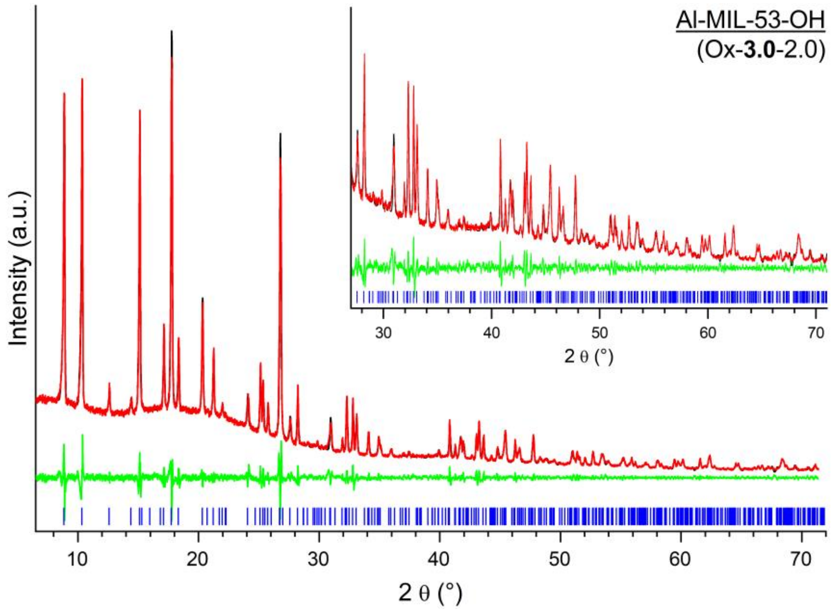

Figure S3.4. Final Le Bail-plot for Al-MIL-53-OH (Ox-3.0-2.0) against PXRD data. The experimental data (red line), the calculated diffractogram (black line), the resulting difference (green line) and the allowed Bragg reflection positions (blue ticks) are shown.

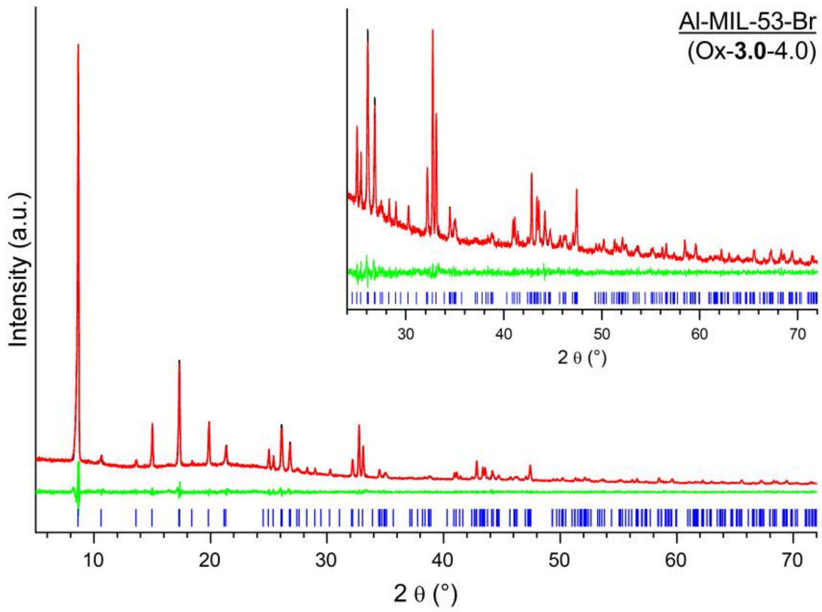

Figure S3.5. Final Le Bail-plot for Al-MIL-53-Br (Ox-3.0-4.0) against PXRD data. The experimental data (red line), the calculated diffractogram (black line), the resulting difference (green line) and the allowed Bragg reflection positions (blue ticks) are shown.

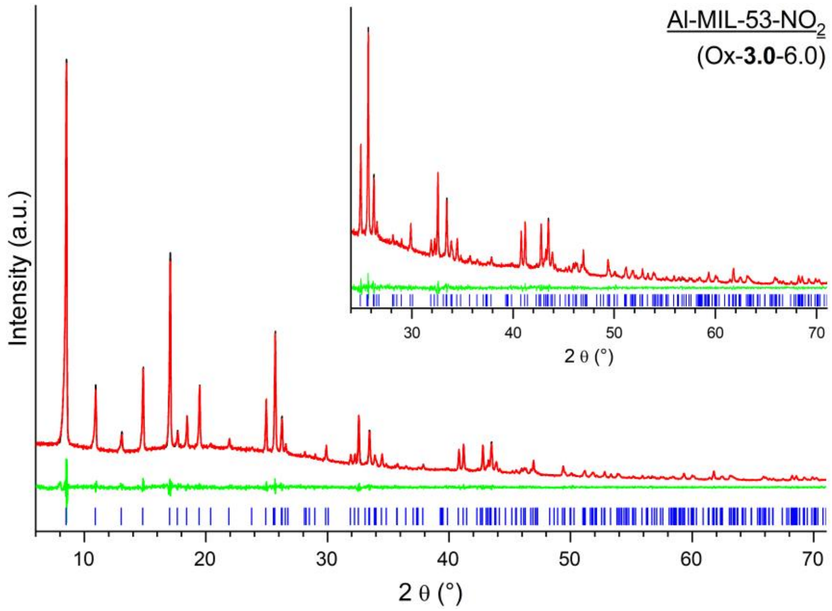

Figure S3.6. Final Le Bail-plot for Al-MIL-53-NO_2 (Ox-3.0-6.0) against PXRD data. The experimental data (red line), the calculated diffractogram (black line), the resulting difference (green line) and the allowed Bragg reflection positions (blue ticks) are shown.

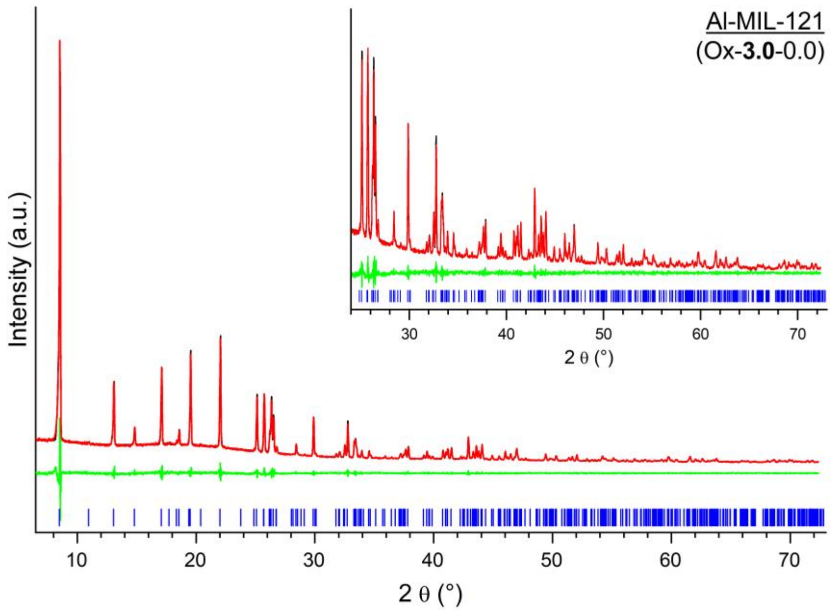

Figure S3.7. Final Le Bail-plot for Al-MIL-121 (Ox-3.0-0.0) against PXRD data. The experimental data (red line), the calculated diffractogram (black line), the resulting difference (green line) and the allowed Bragg reflection positions (blue ticks) are shown.

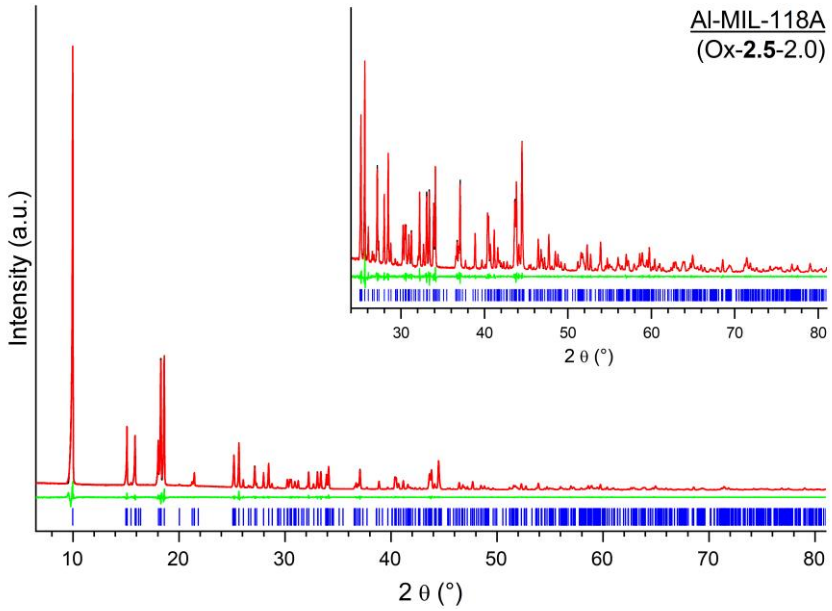

Figure S3.8. Final Le Bail-plot for Al-MIL-118A (Ox-2.5-2.0) against PXRD data. The experimental data (red line), the calculated diffractogram (black line), the resulting difference (green line) and the allowed Bragg reflection positions (blue ticks) are shown.

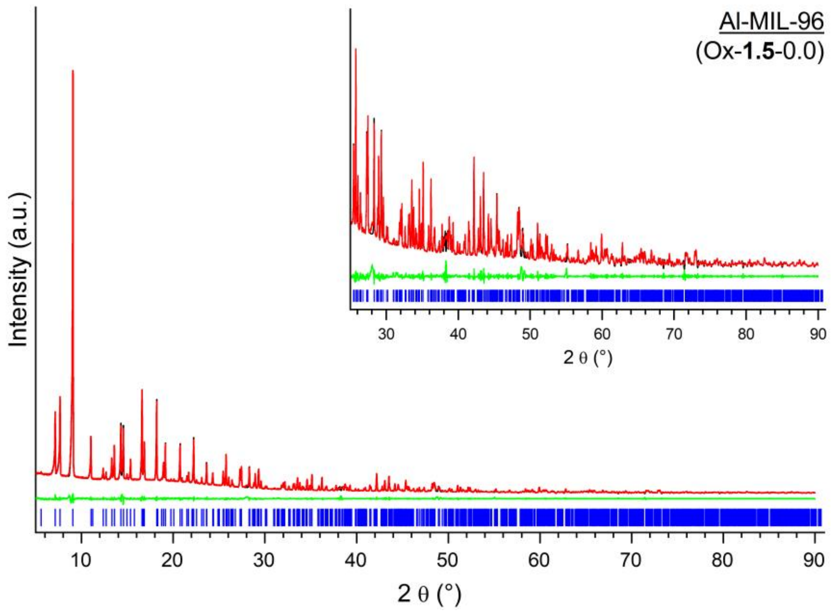

Figure S3.9. Final Le Bail-plot for Al-MIL-96 (Ox-1.5-0.0) against PXRD data. The experimental data (red line), the calculated diffractogram (black line), the resulting difference (green line) and the allowed Bragg reflection positions (blue ticks) are shown.

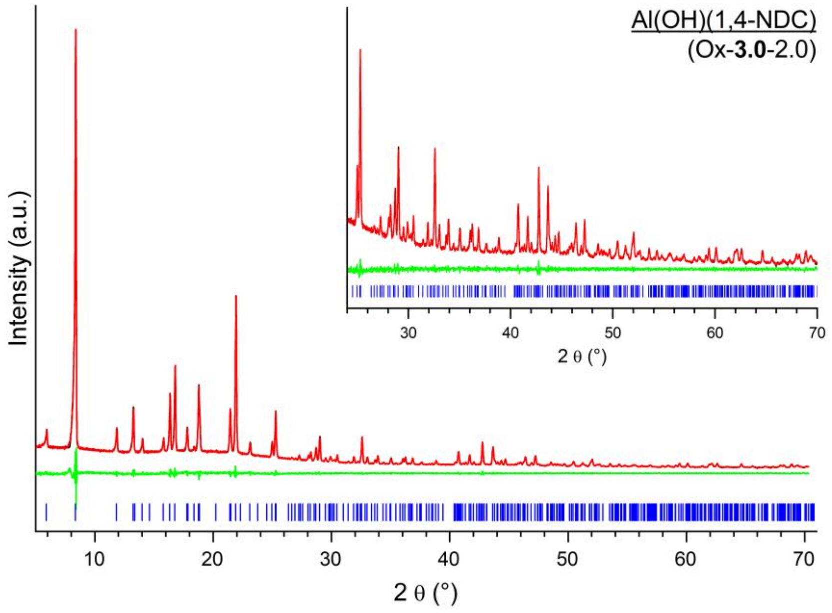

Figure S3.10. Final Le Bail-plot for [Al(OH)(1,4-NDC)] (Ox-3.0-2.0) against PXRD data. The experimental data (red line), the calculated diffractogram (black line), the resulting difference (green line) and the allowed Bragg reflection positions (blue ticks) are shown.

Table S3.3. Selected crystallographic data for Al-MIL-53_*as* (Ox-1.5-4.0) obtained from the Le Bail fit against PXRD data (Figure S3.3). The crystallographic data of Al-MIL-53_*as* reported in the literature[4,10] are given for comparison.

**Al-MIL-53_*as***		
	**Ox-1.5-4.0**	**Literature[4,10]**
**Wavelength** (Å)	1.5406 Å	/
**Crystal system**	Orthorhombic	Orthorhombic
**Space group**	*Pnma*	*Pnma*
a (Å)	17.1194(4)	17.129(2)
b (Å)	12.1788(3)	6.6284(6)
c (Å)	6.6216(13)	12.1816(8)
α (°)	90	90
β (°)	90	90
γ (°)	90	90
**Volume** ($Å^3$)	1380.57(6)	1383.1
R_{wp} (%)	4.17	/
**GoF** (%)	1.09	/

Table S3.4. Selected crystallographic data for Al-MIL-53-OH (Ox-3.0-2.0) obtained from the Le Bail fit against PXRD data (Figure S3.4).

**Al-MIL-53-OH**	
	**Ox-3.0-2.0**
**Wavelength** (Å)	1.5406 Å
**Crystal system**	Orthorhombic
**Space group**	*Pna*21
a (Å)	17.1335(5)
b (Å)	12.2945(3)
c (Å)	6.6683(16)
α (°)	90
β (°)	90
γ (°)	90
**Volume** ($Å^3$)	1404.66(7)
R_{wp} (%)	4.97
**GoF** (%)	1.61

Table S3.5. Selected crystallographic data for Al-MIL-53-Br (Ox-3.0-4.0) obtained from the Le Bail fit against PXRD data (Figure S3.5). The crystallographic data of Al-MIL-53-Br reported in the literature[11] are given for comparison.

Al-MIL-53-Br		
	**Ox-3.0-4.0**	**Literature[11]**
**Wavelength** (Å)	1.5406 Å	1.5406 Å
**Crystal system**	Orthorhombic	Orthorhombic
**Space group**	*Ima*2	/
a (Å)	16.6650(4)	16.580(7)
b (Å)	13.0038(5)	13.243(5)
c (Å)	6.6344(14)	6.666(6)
α (°)	90	90
β (°)	90	90
γ (°)	90	90
**Volume** ($Å^3$)	1437.72(7)	1453.2(12)
R_{wp} (%)	4.09	/
**GoF** (%)	0.80	/

Table S3.6. Selected crystallographic data for Al-MIL-53-NO_2 (Ox-3.0-6.0) obtained from the Le Bail fit against PXRD data (Figure S3.6). The crystallographic data of Al-MIL-53-NO_2 reported in the literature[9] are given for comparison.

Al-MIL-53-NO_2		
	**Ox-3.0-6.0**	**Literature[9]**
**Wavelength** (Å)	1.5406 Å	1.5406 Å
**Crystal system**	Orthorhombic	Orthorhombic
**Space group**	*Ima*2	*Imcm*
a (Å)	16.2250(4)	16.240(2)
b (Å)	13.5585(3)	13.886(2)
c (Å)	6.6610(12)	6.7026(9)
α (°)	90	90
β (°)	90	90
γ (°)	90	90
**Volume** ($Å^3$)	1465.3(6)	1511.5(4)
R_{wp} (%)	2.95	/
**GoF** (%)	0.75	/

Table S3.7. Selected crystallographic data for Al-MIL-121 (Ox-3.0-0.0) obtained from the Le Bail fit against PXRD data (Figure S3.7). The crystallographic data of Al-MIL-121 reported in the literature[6,12] are given for comparison.

**Al-MIL-121**		
	**Ox-3.0-0.0**	**Literature[6,12]**
**Wavelength** (Å)	1.5406 Å	0.7285 Å
**Crystal system**	Monoclinic	Monoclinic
**Space group**	*C*2/*c*	*C*2/*c*
a (Å)	17.5492(3)	17.54483(8)
b (Å)	13.5631(3)	13.57813(8)
c (Å)	6.6668(11)	6.66420(4)
α (°)	90	90
β (°)	113.059(14)	113.1956(5)
γ (°)	90	90
**Volume** ($Å^3$)	1460.03(5)	1459.25(2)
R_{wp} (%)	4.91	/
**GoF** (%)	0.87	/

Table S3.8. Selected crystallographic data for Al-MIL-118A (Ox-2.5-2.0) obtained from the Le Bail fit against PXRD data (Figure S3.8). The crystallographic data of Al-MIL-118A reported in the literature[5,13] are given for comparison.

**Al-MIL-118A**		
	**Ox-2.5-2.0**	**Literature[5,13]**
**Wavelength** (Å)	1.5406 Å	0.96130 Å
T (K)	298 K	100(2) K
**Crystal system**	Monoclinic	Monoclinic
**Space group**	*C*2/*c*	*C*2/*c*
a (Å)	11.43872(8)	11.365(3)
b (Å)	6.82675(5)	6.822(2)
c (Å)	17.7060(15)	17.733(2)
α (°)	90	90
β (°)	91.0597(4)	90.62(4)
γ (°)	90	90
**Volume** ($Å^3$)	1382.42(2)	1374.8(6)
R_{wp} (%)	4.63	/
**GoF** (%)	0.51	/

Table S3.9. Selected crystallographic data for Al-MIL-96 (Ox-1.5-0.0) obtained from the Le Bail fit against PXRD data (Figure S3.9). The crystallographic data of Al-MIL-96 reported in the literature[7,14] are given for comparison.

**Al-MIL-96**		
	**Ox-1.5-0.0**	**Literature[7,14]**
**Wavelength** (Å)	1.5406 Å	0.8266 Å
T (K)	298 K	100(2) K
**Crystal system**	Hexagonal	Hexagonal
**Space group**	*P*63	*P*63/*mmc*
a (Å)	14.30816(8)	14.290(2)
b (Å)	14.30816(8)	14.290(2)
c (Å)	31.5355(3)	31.300(6)
α (°)	90	90
β (°)	90	90
γ (°)	120	120
**Volume** ($Å^3$)	5591.10(8)	5535.3(19)
R_{wp} (%)	3.79	/
**GoF** (%)	0.56	/

Table S3.10. Selected crystallographic data for [Al(OH)(1,4-NDC)] (Ox-3.0-2.0) obtained from the Le Bail fit against PXRD data (Figure S3.10). The crystallographic data of [Al(OH)(1,4-NDC)] reported in the literature[8,15] are given for comparison.

**[Al(OH)(1,4-NDC)]**		
	**Ox-3.0-2.0**	**Literature[8,15]**
**Wavelength** (Å)	1.5406 Å	0.8007 Å
T (K)	298 K	220 K
**Crystal system**	Tetragonal	Tetragonal
**Space group**	*P*4/*nmm*	*P*4/*nmm*
a (Å)	21.1486(2)	21.1012(7)
b (Å)	21.1486(2)	21.1012(7)
c (Å)	6.6228(11)	6.6095(4)
α (°)	90	90
β (°)	90	90
γ (°)	90	90
**Volume** ($Å^3$)	2962.17(8)	2942.9(2)
R_{wp} (%)	3.95	/
**GoF** (%)	0.70	/

4. Spectroscopic Characterization and Thermal Properties

4.1. IR Spectroscopy

IR-spectra (Figure S4.1 – Figure S4.2) of the title compounds synthesized using the optimized synthesis conditions (Section S2) using acetic acid, malonic acid and oxalic acid as the coordination modulator were collected using a Bruker ALPHA-FT-IR A220/D-01 with an ATR-unit. The characteristic vibration bands are assigned in Table S4.1 and Table S4.2.[11,16,17]

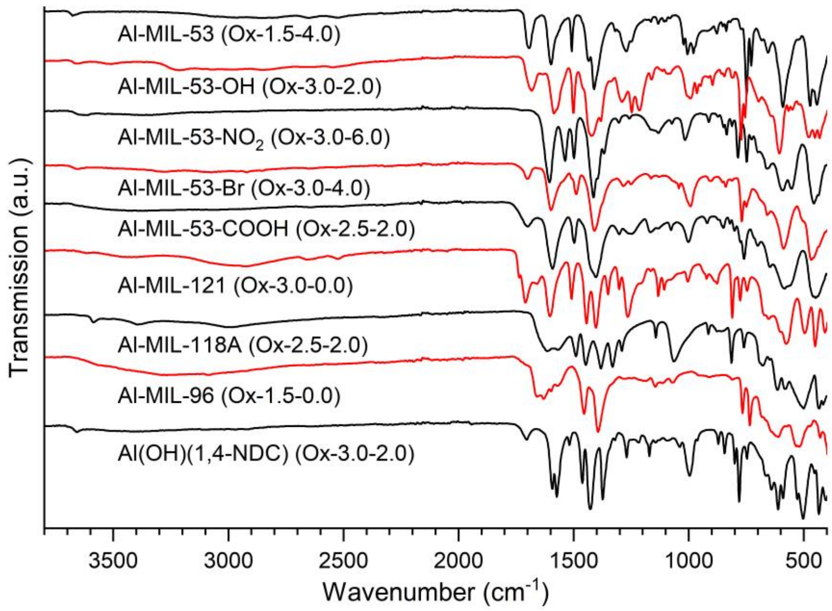

Figure S4.1. FT-IR spectra of the title compounds synthesized using the optimized synthesis conditions. The characteristic vibration bands are assigned in Table 4.1.

Table S4.1. Assignment of the vibrational bands of the title compounds identified in the IR spectra (Figure S4.1) using literature data.[11,16,17]

**Vibration** (cm^{-1})	**Al-MIL-53 (Ox-1.5-4.0)**	**Al-MIL-53-OH (Ox-3.0-2.0)**	**Al-MIL-53-NO$_2$ (Ox-3.0-6.0)**	**Al-MIL-53-Br (Ox-3.0-4.0)**	**Al-MIL-53-COOH (Ox-2.5-2.0)**	**Al-MIL-121 (Ox-3.0-0.0)**	**Al-MIL-118A (Ox-2.5-2.0)**	**Al-MIL-96 Ox-1.5-0.0)**	**Al(OH)(1,4-NDC) (Ox-3.0-2.0)**
ν_{as}(OH) [μ_2-OH]	3675	3660	3660	3660	3660	/		/	3660
ν_{as}(OH) [H_2O]	3100 – 3650								
ν_{as}(C=O) [CO_2H]	1692	1682	/	1698	1698	1737, 1709	/	1712	1702
ν_{as}(C=O) [CO_2^-]	1598	1590	1605	1599	1592	1603	1630-1578	1630-1578	1596, 1573
ν_s(C=C) [C=$C_{arom.}$]	1509	1502	1501	1488	1498	1509	1493	1457	1465
ν_s(C=O) [CO_2^-],[CO_2H]	1420, 1267	1425, 1257	1412, /	1409, 1277	1407, 1260	1440-1400, 1260	1447, 1382, 1285	1394	1423, 1269
δ(C-H) [C-$H_{arom.}$]	745 – 728	771 – 755	785 – 745	770 – 745	777 – 745	777 – 746	810 – 760	766 – 736	788 – 745
δ(C-C) [C-$C_{arom.}$]	475 – 445	485 – 431	490 – 415	480 – 420	482 – 429	495 – 435	550 – 460	550 – 498	502, 435

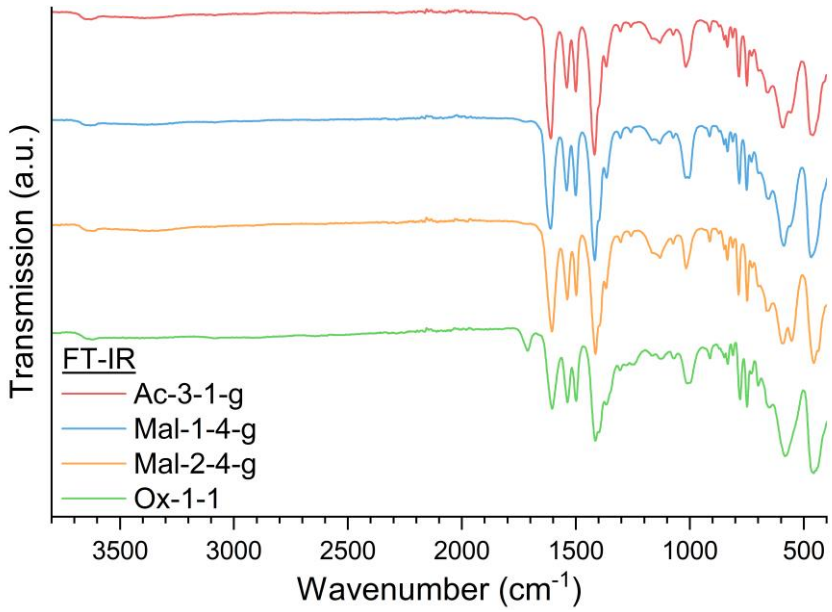

Figure S4.2. FT-IR spectra of Al-MIL-53-NO_2 obtained with four different particle sizes (d ≈ 550 nm – 125 µm) used for the sorption measurements (Section S5). The characteristic vibration bands are assigned in Table 4.2.

Table S4.2. Assignment of the vibrational bands of the title compounds identified in the IR spectra of Al-MIL-53-NO_2 (Figure S4.2).

**Vibration** (cm^{-1})	**Al-MIL-53-NO_2 (Ac-3-1-g)**	**Mal-1-4-g**	**Mal-2-4**	**Ox-1-1**
ν_{as}(OH) [μ_2-OH]	3660			
ν_{as}(OH) [H_2O]	3100 – 3650			
ν_{as}(C=O) [CO_2H]	/	/	/	1711
ν_{as}(C=O) [CO_2^-]	1610	1610	1605	1605
ν_s(C=C) [C=$C_{arom.}$]	1501			
ν_s(C=O) [CO_2^-],[CO_2H]	1420			
δ(C-H) [C-$H_{arom.}$]	785 – 745			
δ(C-C) [C-$C_{arom.}$]	490 – 415			

4.2. NMR Spectroscopy

For Al-MIL-53-NO_2, synthesized using different coordination modulators, ^{1}H-NMR spectra (Figure S4.3 – S4.5) were recorded to confirm the integrity of the linker molecule and the absence of the coordination modulators in the final product. For Al-MIL-53-NO_2 synthesized using oxalic acid as the coordination modulator, an additional ^{13}C-NMR spectra was recorded to confirm the absence of oxalic acid/oxalate ions (Figure S4.5b – c). The bands in the NMR spectra were assigned to the linker molecule, residual solvents (ethanol) and possible modulator residues using literature data.[18,19] The signal of low intensity at ~0.8 ppm in the ^{1}H NMR spectra could not be assigned to any of the reagents used, and no coupling with C-atoms could be observed in the 2D-^{1}H-^{13}C HSQC spectrum (Figure S4.5c). Therefore, the signal was assigned to an impurity in the Teflon® inlet used for the measurements. The additional signal at 7.65 ppm in all ^{1}H NMR spectra can be attributed to trace amounts of terephthalic acid (< 1%), which is present as an impurity in H_2BDC-NO_2 used as the starting materials, which was commercially obtained and used without further purification.

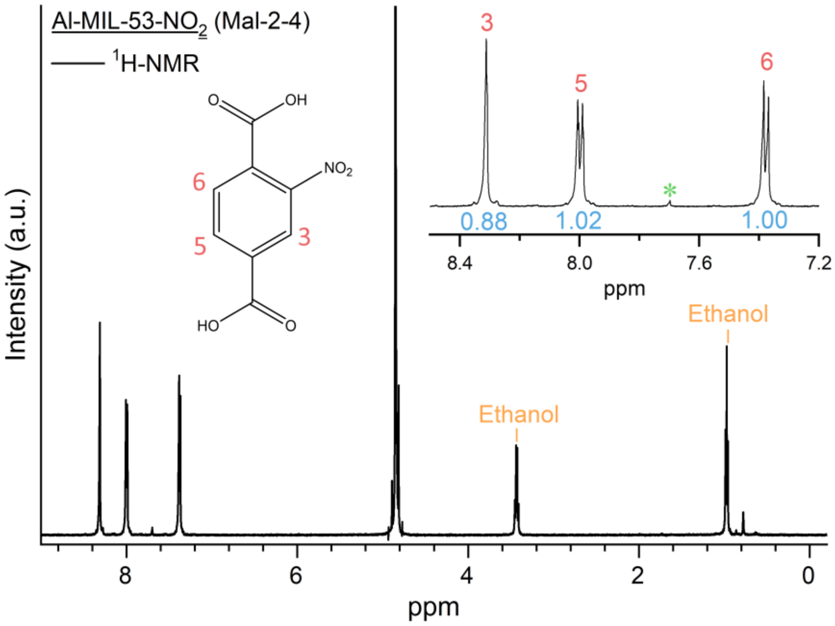

Figure S4.3. ^{1}H-NMR spectra of Al-MIL-53-NO_2 (Ac-3-1). Prior to the measurement, the sample was dissolved in NaOD/D_2O (5 %).

^{1}H-NMR (500 MHz, 298 K, NaOD/D_2O (5 %)): δ = 8.31 (s, 1 H, *H*-3), 7.99 (d, $^3J_{5\text{-}6}$ = 7.6 Hz, 1 H, *H*-5), 7.37 (d, $^3J_{6\text{-}5}$ = 7.2 Hz, 1 H, *H*-6) ppm.

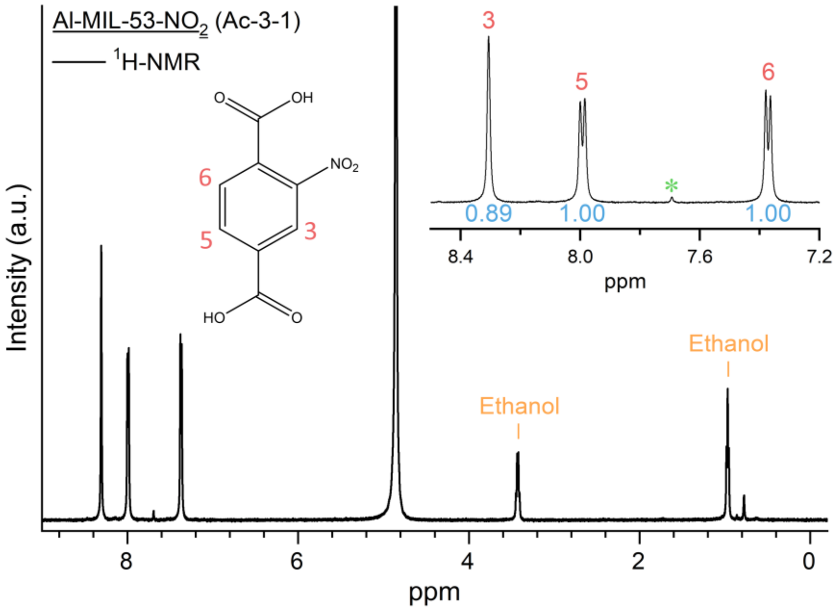

Figure S4.4. ^{1}H-NMR spectra of Al-MIL-53-NO_2 (Mal-2-4). Prior to the measurement, the sample was dissolved in NaOD/D_2O (5 %).

^{1}H-NMR (500 MHz, 298 K, NaOD/D_2O (5 %)): δ = 8.31 (s, 1 H, *H*-3), 8.00 (d, $^3J_{5\text{-}6}$ = 7.8 Hz, 1 H, *H*-5), 7.38 (d, $^3J_{6\text{-}5}$ = 7.8 Hz, 1 H, *H*-6) ppm.

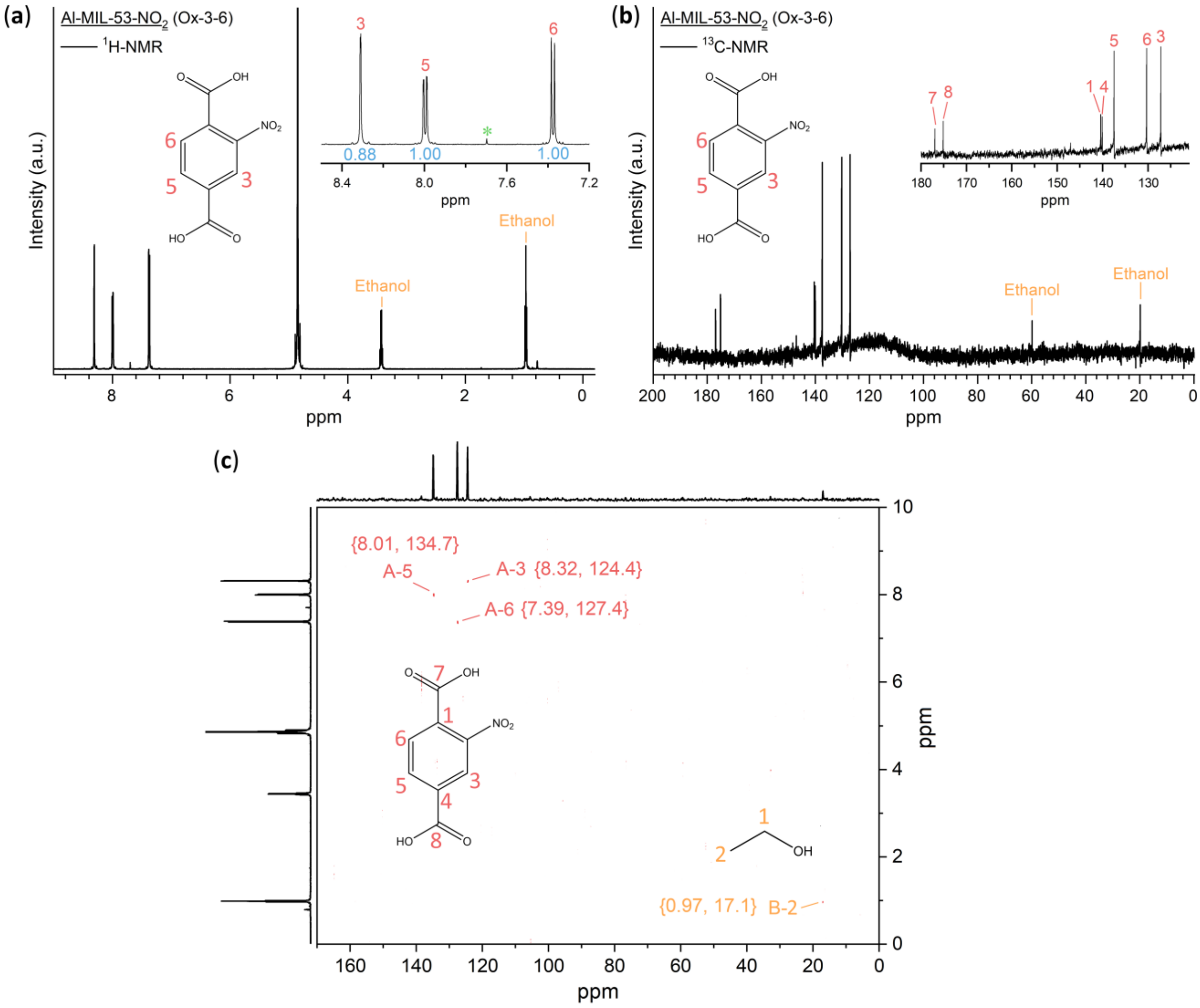

Figure S4.5. ^{1}H-NMR (**a**), 13C-NMR (**b**) spectra of Al-MIL-53-NO$_2$ (Ox-3-6). (**c**) 2D-^{1}H-^{13}C HSQC spectrum showing H-C resonances of the linker molecules and residues of ethanol in the sample. Prior to the measurement, the sample was dissolved in NaOD/D$_2$O (5 %).

^{1}H-NMR (500 MHz, 298 K, NaOD/D$_2$O (5 %)): δ = 8.32 (s, 1 H, *H*-3), 8.01 (d, $^{3}J_{5\text{-}6}$ = 8.0 Hz, 1 H, *H*-5), 7.39 (d, $^{3}J_{6\text{-}5}$ = 7.6 Hz, 1 H, *H*-6) ppm.

^{13}C-NMR (125 MHz, 298 K, NaOD/D$_2$O (5 %)): δ = 176.9 (s, 1 C, *C*-7),, 175.1 (s, 1 C, *C*-8), 140.4 (s, 1 C, *C*-1), 140.1 (s, 1 C, *C*-4), 137.5 (s, 1 C, *C*-5), 130.3 (s, 1 C, *C*-6), 127.2 (s, 1 C, *C*-3) ppm.

4.3. Thermogravimetric Measurements

In addition to NMR spectroscopy, thermogravimetric measurements (Figures S4.6 – S4.8) were performed for Al-MIL-53-NO_2, synthesized using different coordination modulators, to determine the possible incorporation of coordination modulators into the crystal structures, which could lead to linker defects. The thermogravimetric measurements were performed on a Linseis STA PT 1000 (airflow = 6 dm^3/h, heating rate = 8 K/min). The sample amount was approximately 25 mg for each sample and PXRD patterns collected after the measurements indicated the formation of a reaction product of low crystallinity, which can be assigned to Al_2O_3 for all compounds. The composition of the samples of the samples (Table S4.4.) were derived under consideration of the theoretical sum formula of Al-MIL-53-NO_2, IR as well as ^{1}H- and ^{13}C-NMR spectroscopy (Section S4.1 and Section S4.2) and the thermogravimetric measurements (Figure S4.6 – S4.8).

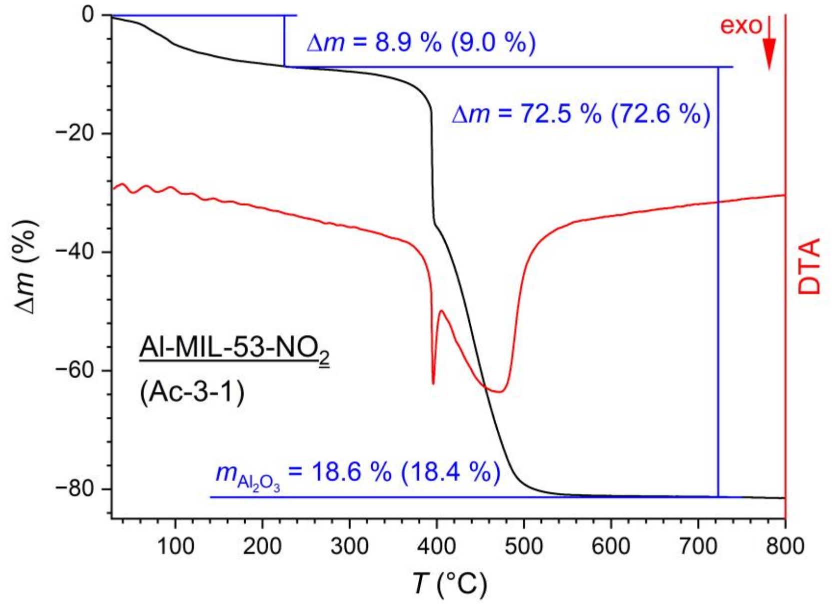

Figure S4.6. Thermogravimetric curve (black), DTA curve (red) and decomposition steps (blue) of Al-MIL-53-NO_2 (Ac-3-1) measured with a heating rate of 8 K/min in air atmosphere. The theoretical values calculated using the sum formula [Al(OH)($C_8H_3NO_6$)] · 1.4 H_2O are given in brackets.

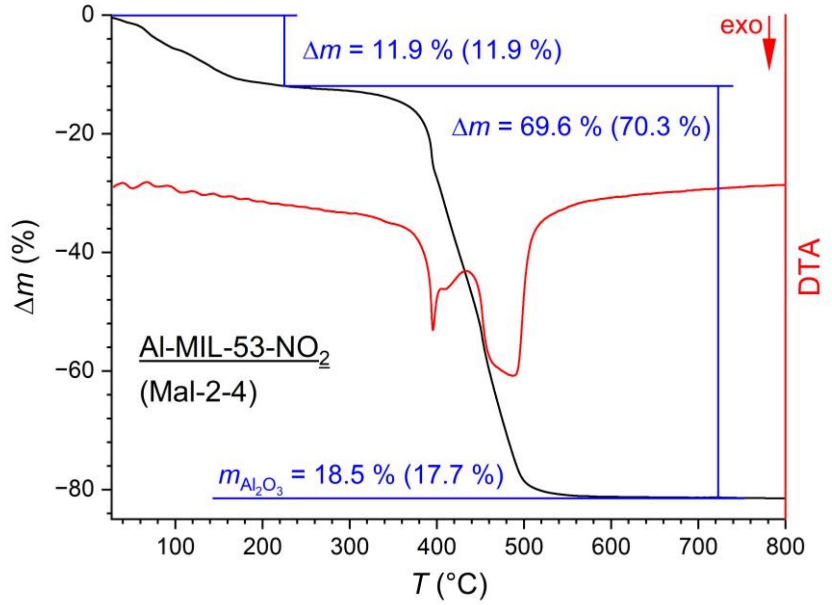

Figure S4.7. Thermogravimetric curve (black), DTA curve (red) and decomposition steps (blue) of Al-MIL-53-NO_2 (Mal-2-4) measured with a heating rate of 8 K/min in air atmosphere. The theoretical values calculated using the sum formula [Al(OH)($C_8H_3NO_6$)] · 1.9 H_2O are given in brackets.

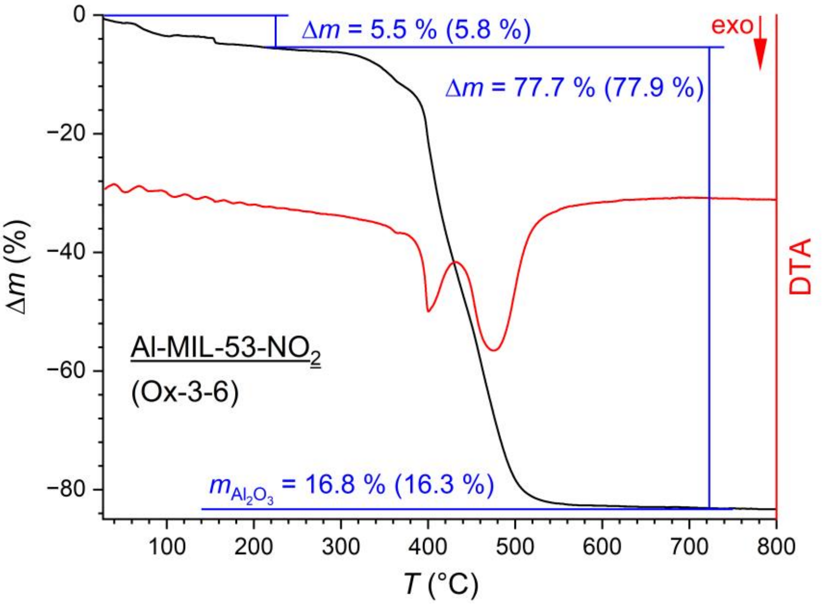

Figure S4.8. Thermogravimetric curve (black), DTA curve (red) and decomposition steps (blue) of Al-MIL-53-NO_2 (Ox-3-6) measured with a heating rate of 8 K/min in air atmosphere. The theoretical values calculated using the sum formula [Al(OH)($C_8H_3NO_6$)] · 0.2 $C_8H_5NO_6$ · 1 H_2O are given in brackets.

Table S4.3. Decomposition steps of the title compounds, determined by thermogravimetric measurements (Figure S4.2 – S4.5). The theoretical values assuming the sum formulas in Table S4.4 are given in square brackets.

	**Al-MIL-53-NO_2 (Ac-3-1)**	**Al-MIL-53-NO_2 (Mal-2-4)**	**Al-MIL-53-NO_2 (Ox-3-6)**
**$\Delta m_{solvent}$** (%)	8.9 [9.0]	11.9 [11.9]	5.5 [5.8]
**$\Delta m_{framework}$** (%)	72.5 [72.6]	69.6 [70.3]	77.7 [77.9]
**$m_{residue}$** (%)	18.6 [18.4]	18.5 [17.7]	16.8 [16.3]

Table S4.4. Derived compositions of the title compounds, determined under consideration of the theoretical sum formula of Al-MIL-53-NO_2, IR and ^{1}H- and ^{13}C-NMR spectroscopy and the thermogravimetric measurements SCXRD, thermogravimetric measurements, elemental analysis and EDX measurements.

**Compound**	**Proposed Sum Formula**	***M*** (g/mol)
**Al-MIL-53-NO_2 (Ac-3-1)**	[Al(OH)($C_8H_3NO_6$)] · 1.4 H_2O	278.3
**Al-MIL-53-NO_2 (Mal-2-4)**	[Al(OH)($C_8H_3NO_6$)] · 1.9 H_2O	287.3
**Al-MIL-53-NO_2 (Ox-3-6)**	[Al(OH)($C_8H_3NO_6$)] · 0.2 $C_8H_5NO_6$ · 1 H_2O	313.3

5. Dynamic Light Scattering (DLS)

For Al-MIL53-NO_2 with particle sizes < 1 µm, the particle size was determined dynamic light scattering (DLS, Figure S5.1). Prior to the DLS measurement the samples were dispersed in ethanol under ultrasonication (5 min, VWR ultrasonic cleaner USC600D).

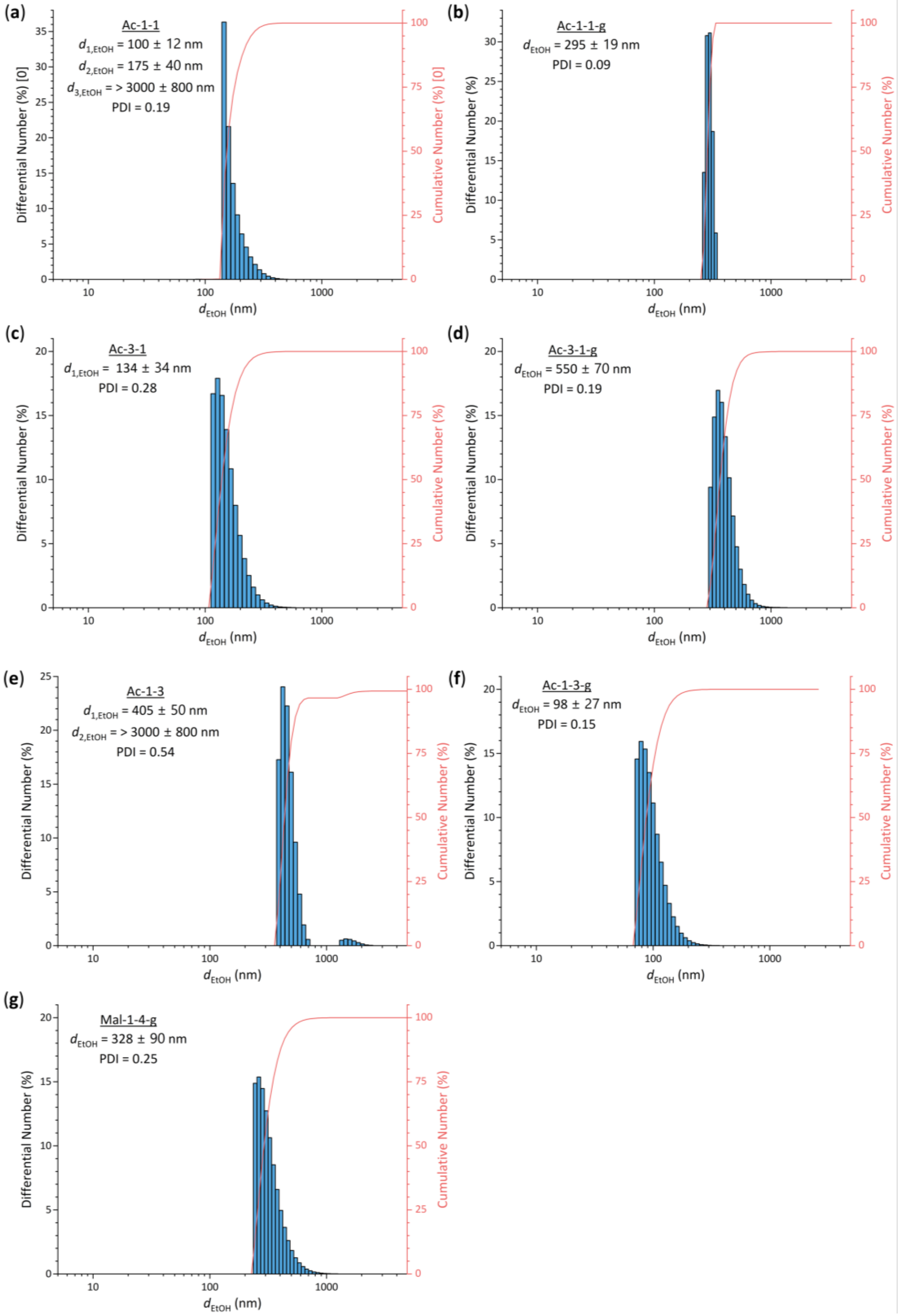

Figure S5.1. Particle size distribution (number distribution) and corresponding particle sizes and polydispersity indices (PDI), obtained using dynamic light scattering (DLS) of different samples of Al-MIL-53-NO_2 (Ac-1-1 (**a**), Ac-1-1-g (**b**), Ac-3-1 (**c**), Ac-3-1-g (**d**), Ac-1-3 (**e**), Ac-1-3-g (**f**), Mal-1-4-g (**g**). Prior to the measurements, the samples were washed with water and ethanol and redispersed using ultrasonication in ethanol. The sample numbers correspond to the reaction conditions listed in tables Table S2.1 and S2.2.

6. Sorption Properties

6.1. Volumetric CO_2 Sorption Measurements

For Al-MIL-53-NO_2, CO_2 sorption measurements (Figure S6.1 – S6.4) were carried out using a BEL Japan Inc. BELSORP-miniX with CO_2 gas at 298 K. Prior to the measurements, the samples were activated for 3 h at elevated temperatures (120 °C) under reduced pressure ($p < 10^{-2}$ kPa). Subsequently, the integrity and the long-range order of the structures after thermal activation was confirmed by PXRD measurements after the sorption measurements (Figure S6.5).

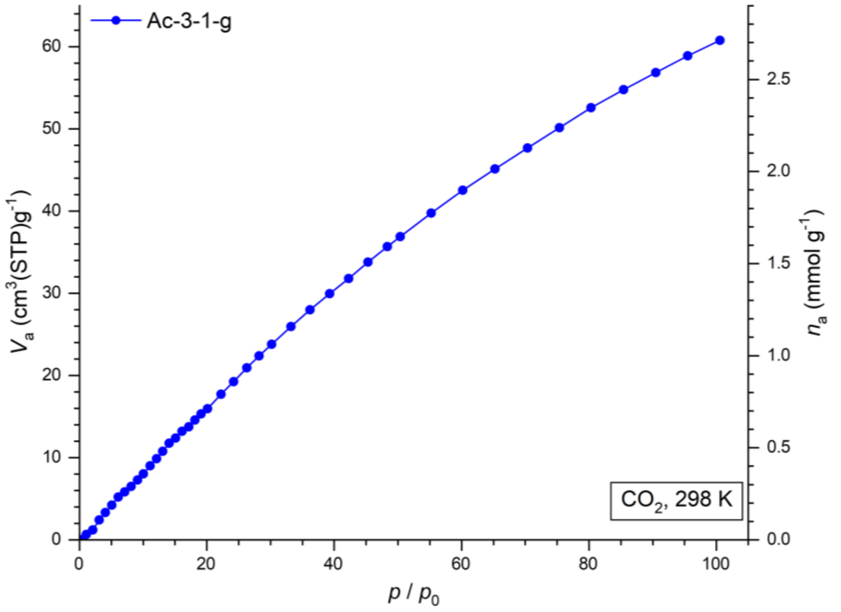

Figure S6.1. CO_2 sorption isotherm of Al-MIL-53-NO_2 obtained using acetic acid as the coordination modulator (Ac-3-1-g), collected at 298 K. Prior to the measurements the sample was activated for 3 h at 120 °C under reduced pressure ($p < 10^{-2}$ kPa).

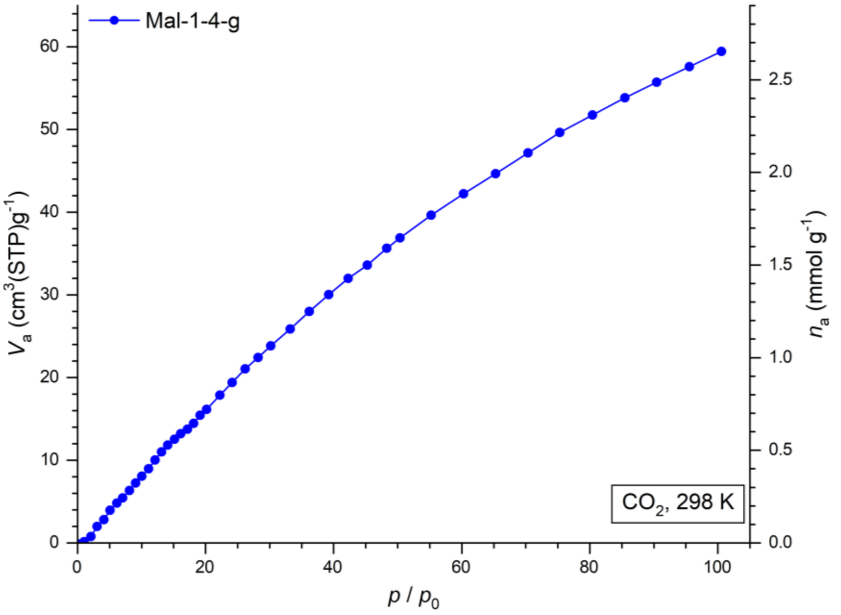

Figure S6.2. CO_2 sorption isotherm of Al-MIL-53-NO_2 obtained using malonic acid as the coordination modulator (Mal-1-4-g), collected at 298 K. Prior to the measurements the sample was activated for 3 h at 120 °C under reduced pressure ($p < 10^{-2}$ kPa).

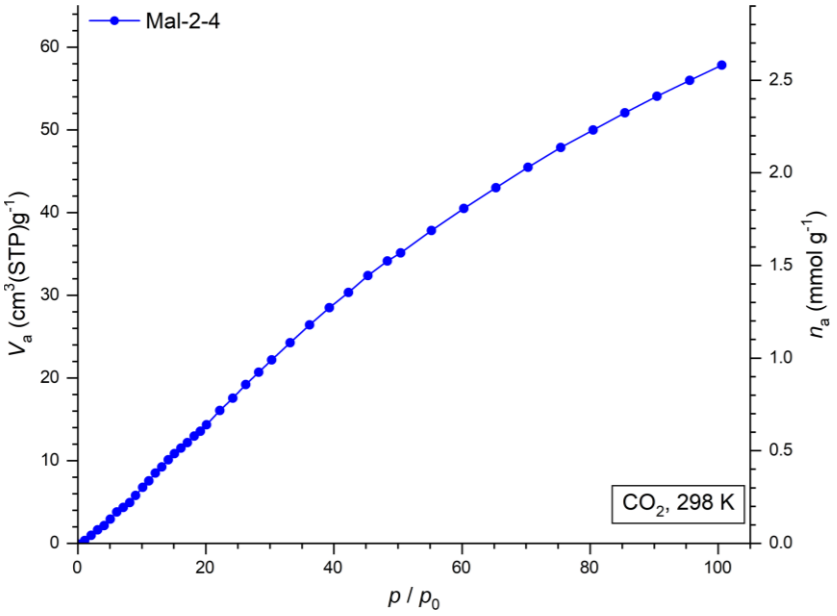

Figure S6.3. CO_2 sorption isotherm of Al-MIL-53-NO_2 obtained using malonic acid as the coordination modulator (Mal2-4), collected at 298 K. Prior to the measurements the sample was activated for 3 h at 120 °C under reduced pressure ($p < 10^{-2}$ kPa).

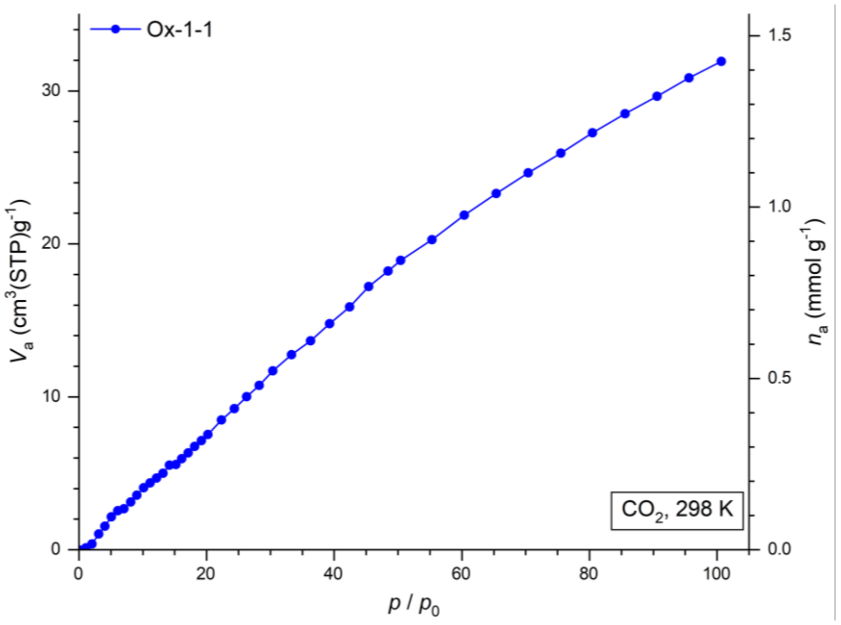

Figure S6.4. CO_2 sorption isotherm of Al-MIL-53-NO_2 obtained using oxalic acid as the coordination modulator (Ox-1-1-g), collected 298 K. Prior to the measurements the sample was activated for 3 h at 120 °C under reduced pressure ($p < 10^{-2}$ kPa).

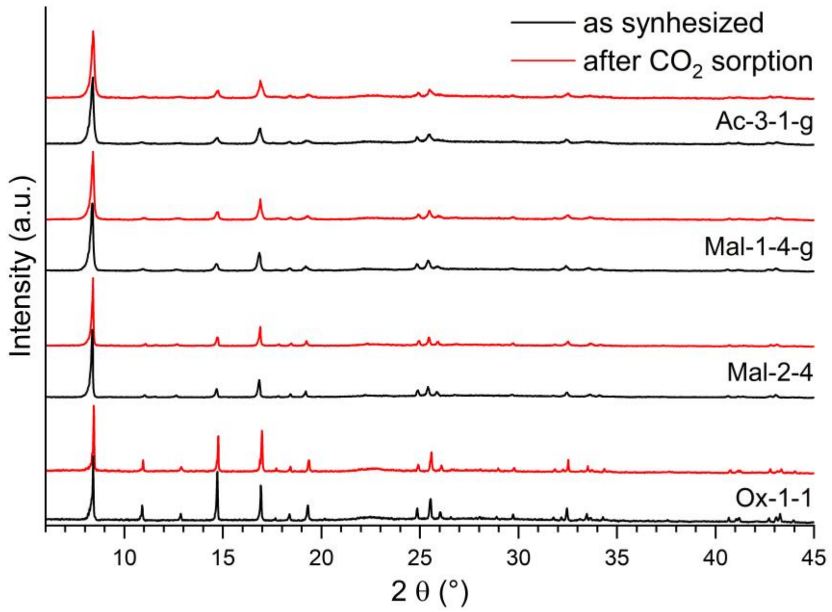

Figure S6.5. PXRD pattern of Al-MIL-53-NO_2 obtained under different synthesis conditions after CO_2 sorption experiments.

6.2. InfraSORP Measurements (Optical Calorimetry)

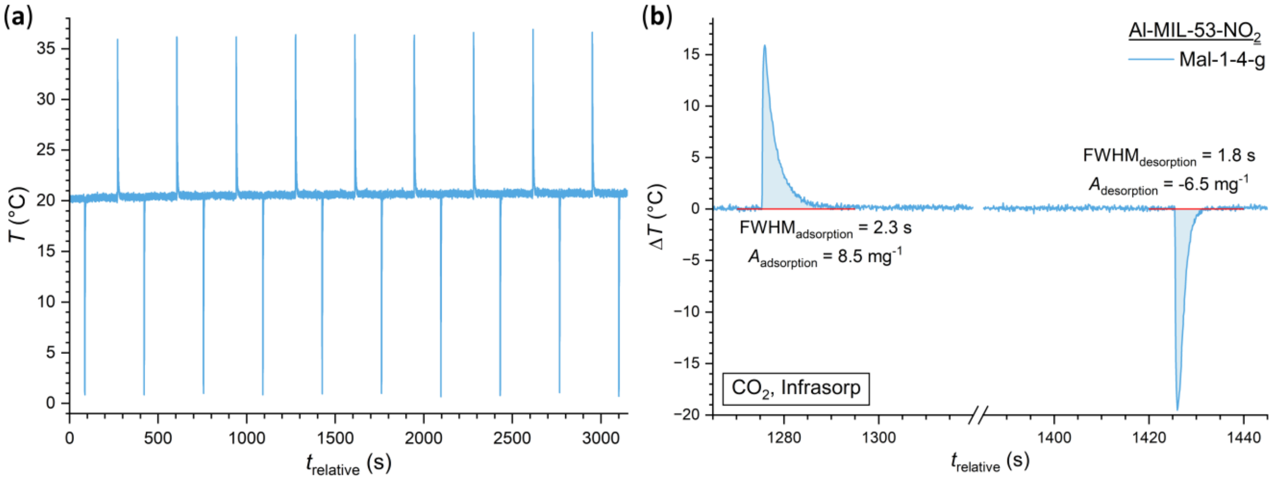

Figure S6.6. (**a**) Temperature profile during CO_2 adsorption and desorption cycling data (10 cycles) of Al-MIL-53-NO_2 (Mal-1-4-g) obtained by optical calorimetry (InfraSORP technology[20,21]) (**b**) Temperature profile with integrated peak areas *A* (normalized to *m*) and FWHM (Full Width at Half Maximum) of the adsorption and desorption at cycle 4.

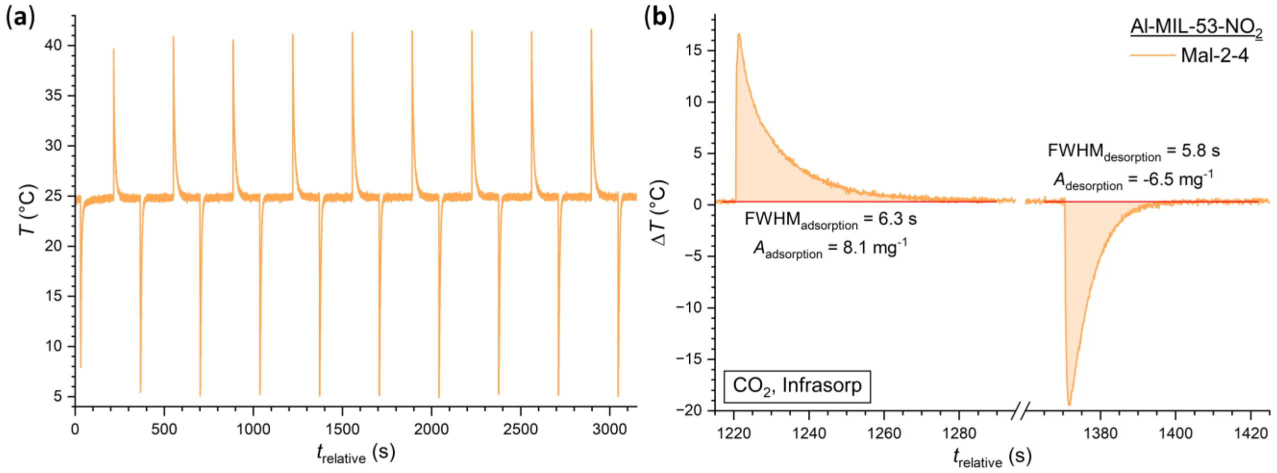

Figure S6.7. (**a**) Temperature profile during CO_2 adsorption and desorption cycling data (10 cycles) of Al-MIL-53-NO_2 (Mal-2-4) obtained by optical calorimetry (InfraSORP technology[20,21]) (**b**) Temperature profile with integrated peak areas *A* (normalized to *m*) and FWHM (Full Width at Half Maximum) of the adsorption and desorption at cycle 4.

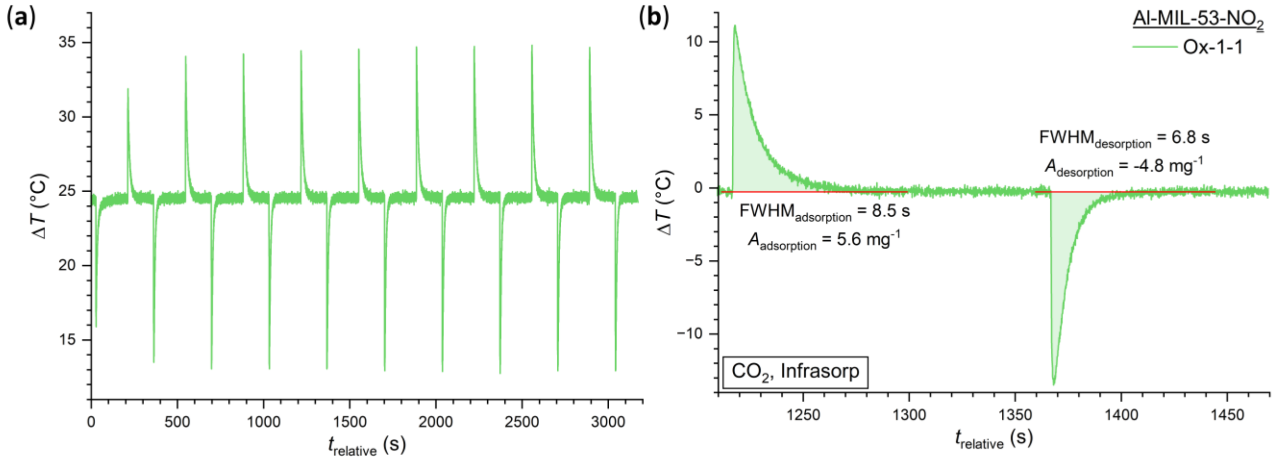

Figure S6.8. (**a**) Temperature profile during CO_2 adsorption and desorption cycling data (10 cycles) of Al-MIL-53-NO_2 (Ox-1-1) obtained by optical calorimetry (InfraSORP technology[20,21]) (**b**) Temperature profile with integrated peak areas *A* (normalized to *m*) and FWHM (Full Width at Half Maximum) of the adsorption and desorption at cycle 4.

7. References

[1] N. Stock, *Microporous Mesoporous Mater.* **2010**, 9.

[2] B. Achenbach, E. S. Grape, M. Wahiduzzaman, S. K. Pappler, M. Meinhart, R. Siegel, G. Maurin, J. Senker, A. K. Inge, N. Stock, *Angew. Chem. Int. Ed.* **2023**, *62*, e202218679.

[3] P. H. Hsu, *Aluminum Hydroxides and Oxyhydroxides*, Soil Science Society Of America, USA, **1989**.

[4] T. Loiseau, C. Serre, C. Huguenard, G. Fink, F. Taulelle, M. Henry, T. Bataille, G. Férey, *Chem. - Eur. J.* **2004**, *10*, 1373–1382.

[5] C. Volkringer, T. Loiseau, N. Guillou, G. Férey, M. Haouas, F. Taulelle, N. Audebrand, I. Margiolaki, D. Popov, M. Burghammer, C. Riekel, *Cryst. Growth Des.* **2009**, *9*, 2927–2936.

[6] C. Volkringer, T. Loiseau, N. Guillou, G. Férey, M. Haouas, F. Taulelle, E. Elkaim, N. Stock, *Inorg. Chem.* **2010**, *49*, 9852–9862.

[7] M. Benzaqui, R. S. Pillai, A. Sabetghadam, V. Benoit, P. Normand, J. Marrot, N. Menguy, D. Montero, W. Shepard, A. Tissot, C. Martineau-Corcos, C. Sicard, M. Mihaylov, F. Carn, I. Beurroies, P. L. Llewellyn, G. De Weireld, K. Hadjiivanov, J. Gascon, F. Kapteijn, G. Maurin, N. Steunou, C. Serre, *Chem. Mater.* **2017**, *29*, 10326–10338.

[8] A. Comotti, S. Bracco, P. Sozzani, S. Horike, R. Matsuda, J. Chen, M. Takata, Y. Kubota, S. Kitagawa, *J. Am. Chem. Soc.* **2008**, *130*, 13664–13672.

[9] A. S. Munn, R. S. Pillai, S. Biswas, N. Stock, G. Maurin, R. I. Walton, *Dalton Trans.* **2016**, *45*, 4162–4168.

[10] T. Loiseau, C. Serre, C. Huguenard, G. Fink, F. Taulelle, M. Henry, T. Bataille, G. Férey, **2004**, DOI 10.5517/CC7DF34.

[11] S. Biswas, T. Ahnfeldt, N. Stock, *Inorg. Chem.* **2011**, *50*, 9518–9526.

[12] Volkringer, C., Loiseau, T., Guillou, N., Ferey, G., Haouas, M., Taulelle, F., Elkaim, E., Stock, N., **2012**, DOI 10.5517/CCYNGHJ.

[13] C. Volkringer, T. Loiseau, N. Guillou, G. Ferey, M. Haouas, F. Taulelle, N. Audebrand, I. Margiolaki, D. Popov, M. Burghammer, C. Riekel, **2010**, DOI 10.5517/CCSYJZ6.

[14] M. Benzaqui, R. S. Pillai, A. Sabetghadam, V. Benoit, P. Normand, J. Marrot, N. Menguy, D. Montero, W. Shepard, A. Tissot, C. Martineau-Corcos, C. Sicard, M. Mihaylov, F. Carn, I. Beurroies, P. L. Llewellyn, G. De Weireld, K. Hadjiivanov, J. Gascon, F. Kapteijn, G. Maurin, N. Steunou, C. Serre, **2018**, DOI 10.5517/CCDC.CSD.CC1PB2Y1.

[15] A. Comotti, S. Bracco, P. Sozzani, S. Horike, R. Matsuda, J. Chen, M. Takata, Y. Kubota, S. Kitagawa, **2009**, DOI 10.5517/CCRTT7L.

[16] G. Socrates, *Infrared and Raman Characteristic Group Frequencies: Tables and Charts*, Wiley, Chichester, **2010**.

[17] N. Reimer, B. Gil, B. Marszalek, N. Stock, *CrystEngComm* **2012**, *14*, 4119.

[18] T. W. Lee, W. J. Lee, Y. S. Kim, T. Do, J.-E. Choi, Y. K. Han, C. Oh, C.-W. Lee, E. K. Yum, J. W. Yang, *Chem. – Eur. J.* **2023**, *29*, e202300903.

[19] G. Hoyez, C. Rousseau, J. Rousseau, S. Saitzek, A. Ponchel, E. Monflier, *Eur. J. Inorg. Chem.* **2022**, *2022*, e202100896.

[20] P. Wollmann, M. Leistner, W. Grählert, O. Throl, F. Dreisbach, S. Kaskel, *Microporous Mesoporous Mater.* **2012**, *149*, 86–94.

[21] F. Sandra, N. Klein, M. Leistner, M. R. Lohe, M. Benusch, M. Woellner, J. Grothe, S. Kaskel, *Ind. Eng. Chem. Res.* **2015**, *54*, 6677–6682.
